# Peptide/Peptoid Hybrid Oligomers: The Influence of Hydrophobicity and Relative Side-Chain Length on Antibacterial Activity and Cell Selectivity

**DOI:** 10.3390/molecules24244429

**Published:** 2019-12-04

**Authors:** Nicki Frederiksen, Paul R. Hansen, Fredrik Björkling, Henrik Franzyk

**Affiliations:** Department of Drug Design and Pharmacology, Faculty of Health and Medical Sciences, University of Copenhagen, Jagtvej 162, DK-2100 Copenhagen, Denmark; nicki.frederiksen@sund.ku.dk (N.F.); prh@sund.ku.dk (P.R.H.); fb@sund.ku.dk (F.B.)

**Keywords:** antimicrobial peptides, peptoids, peptidomimetics, solid-phase synthesis, antibacterial activity, structure-activity study, hemolysis, cell viability, cell selectivity, hydrophobicity

## Abstract

Previous optimisation studies of peptide/peptoid hybrids typically comprise comparison of structurally related analogues displaying different oligomer length and diverse side chains. The present work concerns a systematically constructed series of 16 closely related 12-mer oligomers with an alternating cationic/hydrophobic design, representing a wide range of hydrophobicity and differences in relative side-chain lengths. The aim was to explore and rationalise the structure–activity relationships within a subclass of oligomers displaying variation of three structural features: (i) cationic side-chain length, (ii) hydrophobic side-chain length, and (iii) type of residue that is of a flexible peptoid nature. Increased side-chain length of cationic residues led to reduced hydrophobicity till the side chains became more extended than the aromatic/hydrophobic side chains, at which point hydrophobicity increased slightly. Evaluation of antibacterial activity revealed that analogues with lowest hydrophobicity exhibited reduced activity against *E. coli*, while oligomers with the shortest cationic side chains were most potent against *P. aeruginosa*. Thus, membrane-disruptive interaction with *P. aeruginosa* appears to be promoted by a hydrophobic surface of the oligomers (comprised of the aromatic groups shielding the cationic side chains). Peptidomimetics with short cationic side chains exhibit increased hemolytic properties as well as give rise to decreased HepG2 (hepatoblastoma G2 cell line) cell viability. An optimal hydrophobicity window could be defined by a threshold of minimal hydrophobicity conferring activity toward *E. coli* and a threshold for maximal hydrophobicity, beyond which cell selectivity was lost.

## 1. Introduction

Increased emergence of bacterial strains resistant to antibiotics is a serious worldwide concern for human health. Major factors contributing to the selection for multidrug-resistant (MDR) strains comprise the widespread non-prescription sales of antibiotics in some countries [1] and lack of rapid methods for identification of the specific bacteria causing an infection—with the ensuing prescription of inefficacious antibiotics [2]. Together, these issues promote development of resistance, which ultimately may give rise to pan-resistant strains due to inappropriate use of “last-resort” antibiotics [3,4], and consequently, there is an urgent need for the continuous discovery of new antibiotics. Mechanisms of antimicrobial resistance (AMR) typically confer resistance toward a group of related drugs belonging to the same class of antibiotics. Hence, it is not a viable long-term approach merely to develop analogues of existing drugs, since only entirely new classes of antibiotics may retain a prolonged usage time without the development of severe resistance problems.

Antimicrobial peptides (AMPs) constitute a class of potential antibiotics with an alternative mode of action, and they have evolved to play important roles in the innate immune systems of nearly all living organisms [5], often exhibiting broad-spectrum antibacterial activity [6]. Structurally, AMPs are very diverse, but the majority of known AMPs adopt amphipathic structures and possess a characteristic high overall positive charge, which confers selectivity for bacterial cells over mammalian cells [7,8]. A major challenge in the development of drugs from AMP-based leads is their low proteolytic stability. Approaches that confer increased stability comprise incorporation of D-amino acids [9,10] or design of unnatural mimetics, e.g., α-peptoids (N-alkylated glycine oligomers) [11,12], β-peptoids (N-alkylated β-alanine oligomers) [13], β-peptides [14], α/β^3^-peptides [15], α-peptide/β-peptoid hybrids [16], α/γ N-acylated N-aminoethylpeptides (AApeptides) [17,18], and oligo-acyl-lysyls (OAKs) [19].

Many AMPs fold into α-helices or adopt secondary structures that are amphipathic and considered essential for high antibacterial activity [8,20]. In several studies a correlation between degree of helicity and undesired toxicity of AMPs toward mammalian cells was found [20,21,22,23,24,25,26,27,28,29]. Furthermore, α/β^3^-peptides and peptide/peptoid hybrids with a low degree of amphipathicity and/or secondary structure were shown to possess advantageous activity profiles [15,30]. Interestingly, CD (circular dichroism) spectroscopy proved to be of little value in the characterization of peptide/peptoid hybrid oligomers with an alternating cationic/hydrophobic design, since it was not feasible to assign a secondary structure (e.g., α-helical, β-sheet or random coil) based on their CD spectra, which did not correspond to any known type of folding [30,31]. In addition, crystallisation attempts for such hybrid oligomers have failed so far. Analogues displaying α-chiral side chains in the peptoid residues gave rise to the most distinct CD spectra inferring some degree of secondary structure in the presence of liposomes mimicking bacterial or mammalian membranes [30,31]. This in combination with the finding that oligomers devoid of α-chiral peptoid residues possess the most favourable activity profiles indicate that for these oligomers secondary structure is not a prerequisite for antibacterial activity—in fact it appears only to confer undesirable side effects in the presence of mammalian cells. Moreover, these hybrid oligomers did not exhibit a similar characteristic CD behaviour as reported for peptoid oligomers displaying α-chiral side chains [32].

Both for AMPs and helix-forming α/β^3^-peptide hybrids, increased hydrophobicity has been correlated with higher antibacterial potency and hemolytic properties [15,33,34,35,36,37]. For lysine-based peptide/β-peptoid hybrids and peptoid oligomers, it was found that, above a certain threshold for hydrophobicity, the compounds lost selectivity for bacteria over mammalian erythrocytes [32,38,39]. Various structural modifications that change the overall hydrophobicity of AMPs and peptidomimetics as well as their effects on the biological activity profiles of the resulting analogues have been reported in several studies. These modifications include single amino acid substitutions [40], multiple amino acid substitutions [41], alteration of net charge [34], incorporation of fluorinated residues [42], and appending end groups influencing polarity (i.e., end-tagging with oligopeptides, hydrophobic, polar, or cationic moieties) [43,44,45,46,47,48,49,50,51,52].

Inspired by the above-mentioned observations regarding the putative interplay between activity and hydrophobicity, we decided to perform a more comprehensive study also including the so far poorly explored influence of relative side-chain length as a potential determining factor for antibacterial activity, hemolysis, and effect on cell viability. Previous structure-activity studies of peptide/peptoid hybrids with an alternating cationic/hydrophobic design [53] have shown that such oligomers exert promising antibacterial activity while exhibiting weak hemolytic activity and low effect on mammalian cell viability as compared to the corresponding peptides [31], and that 12-mer oligomers display the optimal cell selectivity [30]. Thus, a series of related 12-mer peptide/peptoid hybrids, displaying elongated/truncated side chains via the incorporation of homologues of lysine/phenylalanine and the corresponding peptoid residues, was investigated in order to identify trends for how structural features influence their activity profile. These peptidomimetics were tested against a panel of seven bacterial strains comprising representatives of the ESKAPE bacteria (*E. faecalis*, *S. aureus*, *K. pneumoniae*, *A. baumannii*, *P. aeruginosa*, and *E. coli*) [54].

## 2. Results and Discussion

### 2.1. Selection and Synthesis of Peptidomimetics

**Figure 1:**
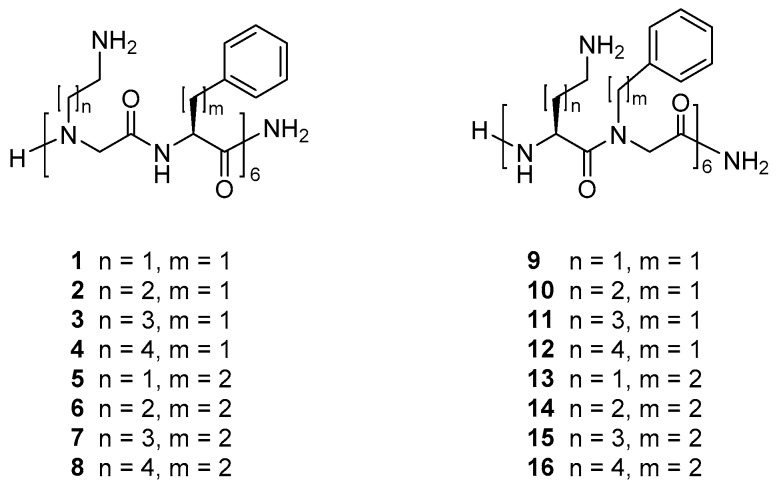
Structures of α-peptide/α-peptoid hybrid peptidomimetics.

Recently, we reported that a peptide/β-peptoid hybrid oligomer with elongated side chains display equal or slightly higher antibacterial activity against *S. pseudintermediu*s and *P. aeruginosa* as compared to the Lys/βNPhe template with identical oligomer length [42,55]. Intrigued by these findings we designed 16 similarly related peptide/α-peptoid oligomers **1**–**16**, see Figure 1, in order to determine whether hydrophobicity and/or relative side-chain length may be clearly correlated to antibacterial potency and/or effect on mammalian cell viability. Comparison with the corresponding peptide was omitted since, for a very closely related 16-mer, both the all-d- and all-l-peptide exhibited exceedingly high toxicity toward mammalian cell lines (i.e., IC_50_ values of ~20 µM) while exhibiting poor antibacterial activity (i.e., MICs of 128 µM or above) [31].

**Figure 2:**
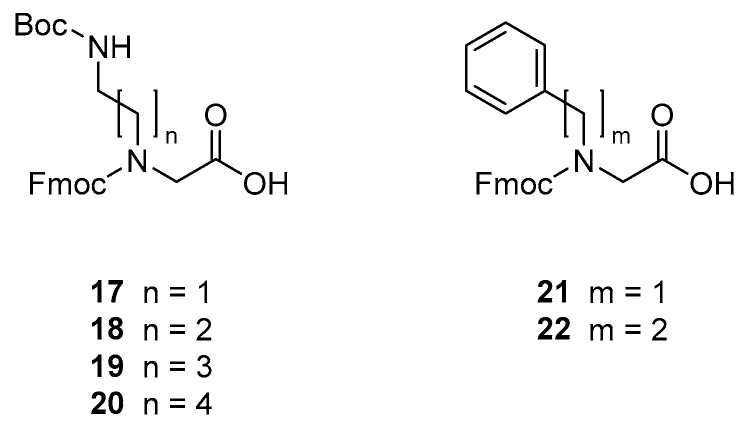
Structures of peptoid building blocks.

The peptidomimetics were synthesised by using Fmoc-based solid-phase peptide synthesis (SPPS) methodology for assembly of commercial amino acid and preformed peptoid building blocks (i.e., **17**–**22**). The Fmoc-protected peptoid building blocks were prepared essentially as previously described [30,56]: The primary amine corresponding to the desired side chain was alkylated with ethyl 2-bromoacetate or *tert*-butyl 2-bromoacetate to provide an intermediate that subsequently was hydrolysed and Fmoc-protected to yield the desired peptoid building block (see Figure 2). Importantly, the cationic peptoid building blocks **17**–**20** could all be obtained in pure state on a multigram scale via an extraction work-up process followed by crystallisation.

### 2.2. Effects of Relative Side-Chain Length and Flexibility on Hydrophobicity

The investigated peptidomimetics (see Figure 1) may be considered to comprise two main groups, consisting either of alternating cationic peptoid residues and hydrophobic amino acid residues (i.e., **1**–**8**) or of alternating cationic amino acid residues and hydrophobic peptoid residues (i.e., **9**–**16**). Four different lengths of the cationic side chains and two different lengths of the hydrophobic side chains were selected in order to obtain analogues within a sufficiently wide range of hydrophobicity so that the entire series was expected to comprise compounds with a substantial variation in antibacterial and/or hemolytic properties. Analogues displaying further extended hydrophobic side chains were not included since analogous peptide/β-peptoid hybrids were found to be poorly soluble in the test media and were found to be devoid of cell selectivity [55].

Thus, the peptidomimetics included in the present study represent variation of three structural features: (i) Cationic side-chain length (2-5 carbon atoms), (ii) hydrophobic side-chain length (benzyl or phenylethyl), and (iii) the type of residue that is of a flexible peptoid nature (i.e., cationic versus hydrophobic). Accordingly, the peptidomimetics can be further subdivided into four subgroups (I–IV), each having similar type of hydrophobic residues and nature of peptoid residues but with varying cationic side-chain length. Thus, subgroups I–IV comprise peptidomimetics **1**–**4**, **5**–**8**, **9**–**12**, and **13**–**16**, respectively.

The relative hydrophobicity of each peptidomimetic was measured by reverse-phase high-performance liquid chromatography (RP-HPLC). Typically, the RP-HPLC retention time (or percentage acetonitrile at peak elution) constitutes the preferred measure that allows for comparison of relative hydrophobicity, while log P values of polycationic peptides or peptidomimetics normally are considered unsuitable due to the extreme hydrophilic nature of such compounds [11,45,57,58,59,60]. Comparison of the hydrophobicity of the members of all four subgroups (see Table 1) indicates that subgroup I (with cationic peptoid residues and Phe, i.e., **1**–**4**) and subgroup II with cationic peptoid residues and homophenylalanine (hPhe) (i.e., **5**–**8**) display the largest differences in hydrophobicity between the least and most hydrophobic representative within each subgroup. For both subgroups III (with cationic amino acid residues and the peptoid analogue of Phe, NPhe, i.e., **9**–**12**) and IV (with cationic amino acid residues and the peptoid analogue of hPhe, NhPhe, i.e., **13**–**16**) only minor differences in hydrophobicity were seen. These observations infer that, when the cationic residues are of a peptoid nature, the side-chain length of these has a strong influence on the hydrophobicity. Expectedly, subgroups I and III, containing either Phe or NPhe, had a relatively low hydrophobicity (~ peak elution within the ranges 35.6–37.5% and 36.9–38.0% MeCN, respectively), while subgroups II and IV, containing hPhe and NhPhe, both displayed a comparably higher hydrophobicity (eluting within the ranges 40.6–43.5% and 40.5–41.4% MeCN, respectively). Thus, introduction of additional six methylene groups results, as expected, in increased hydrophobicity.

Within each subgroup, a somewhat surprising trend is seen — increased cationic side-chain length leads to reduced hydrophobicity. This may be explained by the cationic amino groups becoming gradually more accessible to the surroundings (due to lower shielding by the aromatic groups of the hydrophobic residues) when extending the corresponding side-chain length. In particular, for the peptidomimetics containing 2,4-diaminobutyric acid (Dab) or its peptoid analogue (i.e., NDab) this effect was pronounced, and these analogues was the most hydrophobic ones within each subgroup. When comparing these analogues with those containing ornithine (Orn) or its peptoid analogue (i.e., NOrn), the incorporation of additional methylene groups, expected to confer an increase in hydrophobicity, was clearly counteracted by a larger decrease in hydrophobicity induced by a reduced shielding of the amino functionalities by the aromatic groups, resulting in an overall decrease in hydrophobicity. A similar trend was seen for the hydrophobicity when comparing the peptidomimetics containing Orn or NOrn with the corresponding analogues displaying Lys or the peptoid analogue of Lys (i.e., NLys). By contrast, the peptidomimetic in each group that contained homolysine (hLys) or its peptoid analogue (i.e., NhLys) did not follow this trend, as they consistently possessed a higher hydrophobicity than the corresponding Lys/NLys analogues. For these peptidomimetics, the shielding of the cationic amino groups by the aromatic side chains appears to be surpassed already, and hence the opposite hydrophobicity enhancement conferred by the incorporation of additional methylene groups in the cationic side chains dominates. 

Analysis of similar hydrophobicity data were reported in a previous study concerning 14-mer AMPs containing hydrophobic, branched amino acids and cationic amino acids [57]. Here the relative hydrophobicity of H-Gly-(Ile-Ile-Lys-Lys)_3_-Ile-NH_2_ and H-Gly-(Ile-Ile-Orn-Orn)_3_-Ile-NH_2_ was compared, and the Lys-containing AMP (displaying an additional six methylene groups) was as expected more hydrophobic than the corresponding Orn analogue. However, when comparing the Orn analogue with the Dab analogue, and likewise the Dab analogue with the 2,3-diaminopropionic acid (Dap) analogue, the peptides with the shorter cationic side chains were more hydrophobic, most likely due to the cationic charges being partially shielded by the Ile residues. Interestingly, in the present study, the least hydrophobic compounds within each subgroup were the Lys/NLys analogues (**3**, **7**, **11**, and **15**), while in the above case of Ile-containing peptides, it was the Orn analogue, which may be ascribed to the fact that Phe/NPhe and hPhe/NhPhe all are larger in size than Ile.

### 2.3. Effects of Relative Side-Chain Length and Flexibility on Antibacterial Activity

The antibacterial activity (see Table 1) of the peptidomimetics was tested against a panel of four Gram-negative bacteria (*E. coli*, *K. pneumoniae*, *P. aeruginosa*, and *A. baumannii*) and two Gram-positive bacteria (*E. faecalis* and *S. aureus*). None of the peptidomimetics displayed noteworthy activity against *K. pneumoniae* (MIC values ≥ 32 µg/mL), which corroborates previous findings that Lys-based peptide/peptoid and peptide/β-peptoid hybrids as well as other related peptidomimetics generally lack potency against *K. pneumoniae* [31,58,59,60,61]. Only four peptidomimetics (**8**, **9**, **12**, and **16**) had MIC values below 32 µg/mL against *A. baumannii*, and three of these compounds contained hLys or NhLys, while the most potent oligomers (i.e., **8** and **16** having MIC values of 4 and 8 µg/mL, respectively) contained hPhe or NhPhe, which implies that extended side chains of both cationic and hydrophobic residues appear to confer activity toward *A. baumannii*.

All 16 peptidomimetics were active against *E. coli*, and most of these had a MIC in the range 4–8 µg/mL. Two exceptions were peptidomimetics **2** and **3** with slightly lower potency (i.e., MICs of 8–16 µg/mL) than the other compounds in subgroup I (i.e., **1** and **4** with a MIC of 4-8 µg/mL). Comparison of compounds **2** and **3** with peptidomimetics with the same cationic residues in subgroup II (i.e., **6** and **7** with MIC of 2–4 µg/mL) as well as with the analogues having a reversed amino acid/peptoid design in subgroup III (i.e., **10** and **11** with MICs of 4-8 µg/mL) showed that compounds **2** and **3** exhibited slightly lower antibacterial activity. Slightly higher MIC values against *E. coli* were observed for peptidomimetics in subgroup I as compared to those in subgroup II, possibly due to the additional methylene groups in the side chains of hPhe (as compared to Phe). By contrast all members of subgroups III and IV that displayed cationic amino acid residues were almost equipotent (i.e., MICs within the range 2–8 µg/mL).

Thus, when considering how hydrophobicity influences activity of the analogues against *E. coli* a clear pattern could not be discerned (see Figure S1): while the least hydrophobic compounds **2** and **3** (~ peak elution at 35.7% and 35.6% MeCN, respectively) exhibited poorest antibacterial activity, the most potent peptidomimetics against *E. coli* (i.e., **7**–**9** and **12** with MICs in the range 2–4 µg/mL) were not amongst the analogues with the highest hydrophobicity. Hence, no clear correlation between structural features and antibacterial potency was evident, since **7**–**9** and **12** differed with respect to both relative side-chain length of cationic and hydrophobic residues as well as the nature of peptoid residues. This indicated the existence of a hydrophobicity threshold for optimal activity against *E. coli* as previously found for end-group modified peptide/β-peptoid hybrids [39].

Comparison of subgroup I peptidomimetics with those in subgroup III showed that their hydrophobicity was similar, but since all members of subgroup III were more potent, the hydrophobic nature of the peptoid residues appeared to be a more important contributing factor for high activity against *E. coli* than merely the overall hydrophobicity (see Figure S1). When comparing peptidomimetics from subgroups II and IV their hydrophobicity was similar, but higher than for subgroups I and III, which appeared to confer improved potency against *E. coli*.

Two different strains of *P. aeruginosa* were included in the present study. No significant activity against these was observed for peptidomimetics in subgroup I, which displayed a generally low hydrophobicity, indicating that this property diminishes activity against *P. aeruginosa*. For the remaining subgroups, peptidomimetics with shorter cationic side chains exhibited higher activity against both strains of *P. aeruginosa* than peptidomimetics with longer cationic side chains. Generally, shortening of the cationic side chains by one methylene group conferred a two-fold increased potency. The compounds that proved most potent against *P. aeruginosa* contain hydrophobic peptoid residues, i.e., they belong to subgroups III and IV. Thus, a design with short-chain cationic amino acids and hydrophobic peptoid residues promotes antipseudomonal activity. These findings indicate that efficient membrane-disruptive interaction with *P. aeruginosa* requires that the peptidomimetics possess a hydrophobic “surface” comprised of the aromatic groups (partially) shielding the cationic side chains (see Figure S2). Previously, peptide/β-peptoid analogues of peptidomimetics **9**–**16** were found to exhibit potent activity against *P. aeruginosa* (MIC values in the range of 2–8 µg/mL) when the cationic side chains were short or when the hydrophobic side chains were extended [38,42,55], thus supporting the present findings.

A putative explanation for the higher activity against *E. coli* as compared to the very low activity against *K. pneumoniae* is the higher overall content of anionic phospholipids in the cell membranes of *E. coli* that is likely to make this species more susceptible to the membrane-disruptive actions of these highly cationic peptidomimetics. Thus, in *E. coli*, cardiolipin (CL; with an overall charge of −2) and phosphatidylglycerol (PG; with a net charge of −1) comprise 5% and 15%, respectively, of the total membrane phospholipids, while the content of the zwitterionic phosphatidylethanolamine (PE) is 80% [62]. In contrast, for *K. pneumoniae* the content of the negatively charged CL and PG is 6% and 5%, respectively, resulting in an overall less negatively charged membrane, which may be anticipated to interact less efficiently with these peptidomimetics [63]. Similarly, the phospholipid content in the cell membrane of *P. aeruginosa* comprise 11% CL and 21% PG, conferring a considerably higher negative charge to its membrane as compared to those of both *E. coli* and *K. pneumoniae*. Consequently, all differences in activity cannot readily be explained by their distinct phospholipid compositions alone. Yet, the outer membranes of *P. aeruginosa*, *E. coli* and *K. pneumoniae* differ somewhat with respect to the structure of lipid A that constitutes the inner part of lipopolysaccharide, which by far is the most abundant component of the outer leaflet. While lipid A in *E. coli* and *K. pneumoniae* have very similar structures (only differing in the position and length of a single fatty acid moiety), *P. aeruginosa* lipid A contains additional hydroxyl groups that enable increased hydrogen bonding between neighbouring lipid A molecules, thereby stabilising the outer membrane [64].

The antibacterial activity of the peptidomimetics was also tested against two Gram-positive bacteria (*S. aureus* and *E. faecalis*). Only peptidomimetics **5** and **6** showed moderate activity against *S. aureus*, whereas only **5** had weak activity against *E. faecalis*. Peptidomimetic **5** had the highest hydrophobicity of all analogues, while peptidomimetic **6** was amongst the most hydrophobic members, which is in accordance with previous studies, where a clear relationship was observed between hydrophobicity of peptoids and their activity against Gram-positive bacteria (in particular for *S. aureus*) [47,65]. Earlier studies have found similar trends for peptide/β-peptoid hybrids, for which increased hydrophobicity could be correlated to higher activity against Gram-positive bacteria [38,39,42].

### 2.4. Effects of Relative Side-Chain Length and Flexibility on Mammalian Cell Viability

The effects of the peptidomimetics on the viability of mammalian cells were tested against HepG2 cells and human red blood cells (see Table 2). In subgroup I, the combination of cationic peptoid residues and Phe resulted in a lower hydrophobicity than found for all other subgroups. Generally, the peptidomimetics in this subgroup exerted minimal effects on HepG2 cell viability, since all IC_50_ values were 1280 µg/mL or higher. Similarly, these compounds all exhibited weakly hemolytic properties (i.e., hemolysis below 10% at 800 µg/mL).

For subgroup II (i.e., **5**–**8**) there is a clear trend of an increasing effect on cell viability within the subseries NLys → NOrn → NDab (see Table 2.). For subgroup II further extension of the cationic side-chain length (from NLys in **7** to NhLys in **8**) confers a slightly increased hydrophobicity due to incorporation of additional six methylene groups. This leads to a significantly increased effect of **8** on cell viability, corresponding to a ca. 2.7-fold lowering of the IC_50_ value as compared to that found for **7**. In subgroup II, the most hydrophobic analogue (i.e., **5**) was the only representative giving rise to >10% hemolysis at 800 µg/mL.

Subgroup III (i.e., **9**–**12**) also had relatively low hydrophobicity (see Table 2), and for **9**–**11** we observed a trend of increasing cationic side-chain length conferring gradually decreased hydrophobicity with an ensuing declining effect on HepG2 cell viability. However, even though **9** and **12** had similar hydrophobicity, only **9** (with a hydrophobic surface) had a pronounced effect on HepG2 cell viability and was the most hemolytic compound in the entire array (79% hemolysis at 800 µg/mL). Finally, subgroup **IV** peptidomimetics (i.e., **13**–**16**) were found to possess relatively high hydrophobicity (see Table 2). However, no clear trends, attributable to cationic side-chain length or hydrophobicity, were observed with respect to their effect on HepG2 cell viability or hemolytic activity, except that peptidomimetic **13**, displaying the shortest cationic side chain, also had pronounced hemolytic properties (53% hemolysis at 800 µg/mL). The two peptidomimetics with highest hydrophobicity, namely **13** and **16**, also exerted the strongest effect on HepG2 cell viability. In subgroups II and IV, the lowest IC_50_ values for HepG2 viability were seen for the two peptidomimetics with the shortest and longest cationic side chains, respectively. Also, in subgroups II–IV, the most distinct hemolytic properties were seen for the members displaying a hydrophobic “surface” comprised of the aromatic groups shielding the cationic side chains.

**Figure 3:**
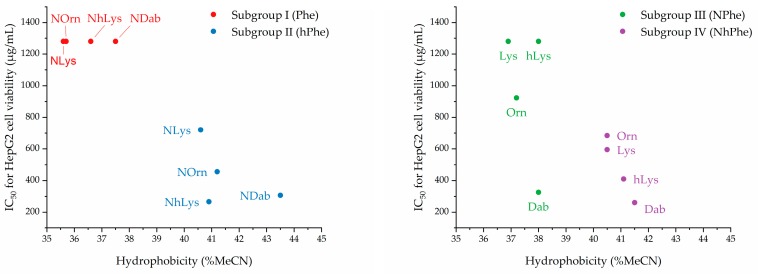
%MeCN at RP-HPLC peak elution versus effect on HepG2 cell viability (IC_50_).

These findings regarding the relationships between hydrophobicity and effect on HepG2 cell viability, dicusssed above for the four subgroups of peptidomimetics, are depicted graphically below in Figure 3. 

The correlation between high hydrophobicity and loss of cell selectivity found for the present series of petidomimetics corroborates findings in a previous study on peptoid oligomers [32]. Here, a relatively small difference in hydrophobicity (also based on % MeCN at peak elution) of the 12-mer H-(NLys-Nspe-Nspe)_4_-NH_2_ and the corresponding 9-mer was observed (53.6% MeCN versus 51.6% MeCN), and the concentration of these peptidomimetics resulting in 10% hemolysis (of human red blood cells) were 9.1 µM and 119.5 µM, respectively. Also, two 12-mer analogues with further increased hydrophobicity (due to incorporation of Nspe-derived residues with either a Me or Cl substituent in the 4-position; ~ peak elution at 61.1% MeCN and 64.1% MeCN, respectively) proved to be exceedingly hemolytic with 10% hemolysis reached below 6.25 µM [32]. Also, moieties within a wide range of polarity have been introduced at the N-terminus of an α-peptide/β-peptoid template in order to evaluate the ensuing effects on the activity profile of the resulting analogues [39]. Thus, analogues with aliphatic or aromatic end-group modifications showed increased antibacterial activity over the template. However, even though these compounds exhibited similar effects on the viability of NIH 3T3 (fibroblast cells, IC_50_ values in the range 35-67 µM) and HUVEC cells (human umbilical vein endothelial cells, IC_50_ values of ~150 µM) their hemolytic properties differed considerably (i.e., 50% hemolysis was reached between 9 µM and 602 µM) as did their hydrophobicity (~ peak elution between 48.8% and 54.3% MeCN). Two compounds eluting below 50% MeCN displayed the lowest effect on mammalian cell viability, and in particular, they exhibited significantly reduced hemolytic properties. Collectively, these findings strongly corroborate the hypothesis that, within a series of closely related compounds, there exists a threshold for acceptable maximal hydrophobicity, beyond which cell selectivity is lost completely.

In several studies, two hydrophobicity thresholds have been identified for both AMPs and antibacterial peptidomimetics [36,41,65,66,67,68,69,70]. Thus, the hydrophobicity of an AMP or peptidomimetic must exceed the lower threshold in order to possess potent antibacterial activity. However, when the higher threshold is surpassed the compound will lose selectivity toward bacteria over mammalian cells. In the present study, a lower hydrophobicity threshold for activity against susceptible Gram-negative bacteria (i.e., *E. coli* and *P. aeruginosa*) could not be identified for *E. coli*, whereas against *P. aeruginosa*, only peptidomimetics with a hydrophobicity above ~37% MeCN proved active. However, for *P. aeruginosa*, this was not the only criteria that should be fulfilled, since peptidomimetics with shorter cationic side chains proved most active, regardless of hydrophobicity.

For subgroups I and III, which both contained cationic peptoid residues and hydrophobic amino acid residues, analogues with a low hydrophobicity (~ to peak elution below ~38% MeCN) exerted a negligible effect on HepG2 cell viability when excluding peptidomimetic **9**. Noticeably, three out of four peptidomimetics containing the shortest cationic side chains (i.e., **5**, **9**, and **13**) had the highest hydrophobicity in their subgroup and showed pronounced hemolysis in the range 43–79% at 800 µg/mL. Interestingly, although **1** and **9** had very similar hydrophobicity (~ peak elution at 37.5% and 38.0% MeCN, respectively) they possess quite different hemolytic properties. This deviation from the common trend may be explained by the fact that **1** contains cationic peptoid residues, while the very hemolytic **9** display hydrophobic peptoid residues. Molecular modelling have suggested that peptoids have a greater diversity of conformational states than peptides [11], which means that the combination of short cationic side chains and flexible hydrophobic side chains are expected to interact more favorably with the cell membranes of red blood cells, thus conferring hemolytic properties even below the critical hydrophobicity threshold for antibacterial activity.

## 3. Materials and Methods

### 3.1. General Information

Starting materials and solvents were purchased from commercial suppliers (Iris Biotech, Markredwitz, Germany; Fluorochem, Hadfield, United Kingdom; and Merck, Darmstadt, Germany) and used without further purification. Water used for analytical and preparative high-performance liquid chromatography (HPLC) was filtered through a 0.22-μm Millipore membrane filter. Purity and retention time of each peptidomimetic were determined by analytical UHPLC by using a Phenomenex Luna C18(2) HST column (100 mm × 3 mm; particle size: 2.5 μm; pore size: 100 Å) on a Shimadzu Prominence and Shimadzu Nexera system using an aqueous acetonitrile (MeCN) gradient with 0.1% trifluoroacetic acid (TFA) added (eluent A: 5:95 MeCN–H_2_O + 0.1% TFA, eluent B: 95:5 MeCN–H_2_O + 0.1% TFA); a flow rate of 0.5 mL/min was used. For elution of peptidomimetics, a linear gradient of 0% to 60% B during 10 min was used with UV detection at λ = 220 nm. All tested compounds had a purity of at least 97%. For each peptidomimetic, the percentage of MeCN at peak elution was calculated from the retention time by using the following formula:

(1) $$\%\mathrm{MeCN}\;=\;0.95\;\left(0.6\frac{R_{t}}{10\;min.}\right)\;+\;0.05\;\left(1-0.6\frac{R_{t}}{10\;min.}\right).$$

Preparative HPLC was performed by using a Phenomenex Luna C18(2) column (250 × 21.2 mm; particle size: 5 μm) on a Shimadzu Prominence system using the same eluents as for analytical HPLC. Elution was performed with a linear gradient of 0% to 40% B during 20 min at a flow rate of 20 mL/min with UV detection at λ = 220 nm. High-resolution mass spectrometry (HRMS) spectra were obtained by using a Bruker Solarix XR MS detector.

### 3.2. Building Block Synthesis

#### 3.2.1. Synthesis of *N*-(((9*H*-fluoren-9-yl)methoxy)carbonyl)-*N*-(5-((*tert*-butoxycarbonyl)amino)ethyl)glycine [Fmoc-NDab(Boc)-OH, **17**]

*Tert*-butyl (5-aminoethyl)carbamate (11.7 g) was dissolved in THF (110 mL), and then Et_3_N (16 mL, 3.0 equiv) was added to the solution under stirring. The mixture was kept at room temperature (rt) for 10 min. Ethyl bromoacetate (12.24 mL, 1 equiv) was dissolved in THF (100 mL) and added dropwise to the solution under stirring. The mixture was kept at rt for 16 h. The mixture was concentrated in vacuo, and then the residue redissolved in Et_2_O (300 mL) and filtered. The filtrate was concentrated in vacuo to afford the intermediate ethyl ester (17.65 g) as a colourless oil, which was dissolved in dioxane (100 mL) and MeOH (40 mL). Then, 4M NaOH (18 mL) was added dropwise under stirring. The mixture was kept at rt for 1 h, then concentrated in vacuo, and the residue redissolved in H_2_O (100 mL). Fmoc-OSu (23.69 g, 1 equiv) was dissolved in warm (45 °C) MeCN (170 mL) and added dropwise to the mixture under stirring. The mixture was kept at rt for 16 h, then concentrated in vacuo until it turned turbid. Then EtOAc (300 mL) was added, and the resulting mixture was washed with 10% citric acid (400 mL). The aqueous phase was extracted with EtOAc (2 × 150 mL), and the combined organic phases were washed with H_2_O (3 × 250 mL), and brine (250 mL). The organic phase was extracted with 10% NaHCO_3_−10% Na_2_CO_3_−dioxane 3:3:2 (4 × 400 mL). The combined aqueous phases were adjusted to pH 2–3 with 4M HCl, extracted with EtOAc (150 mL), then combined, dried over Na_2_SO_4_ and concentrated in vacuo. The resulting solid was recrystallised from EtOAc (200 mL) and heptane (800 mL) to afford building block **17** (25.0 g, 77.6%) as a white solid; t_R_ = 6.53 min. (gradient 30–100% B during 10 min). HRMS: calcd for C_24_H_28_N_2_O_6_ [M + Na]^+^ 463.18396, found 463.18550; ΔM = 3.3 ppm. ^1^H NMR (600 MHz, methanol-*d*_4_) δ 7.79 (m, 2H, Ar*H*-Fmoc), 7.61 (m, 2H, Ar*H*-Fmoc), 7.39 (m, 2H, Ar*H*-Fmoc), 7.31 (m, 2H, Ar*H*-Fmoc), 4.39 (m, 2H, C*H*_2_-Fmoc), 4.24 (m, 1H, C*H*-Fmoc), 3.99 (s, 2H, N-C*H*_2_-C=O)), 3.43 – 3.25 (m, 2H, CH_2_-C*H*_2_-N), 3.03 (t, *J* = 6.2 Hz, 2H, N-C*H*_2_-CH_2_), 1.40 (s, 9H, C*H*_3_-Boc). ^13^C NMR (151 MHz, methanol-*d*_4_) δ 171.73, 156.77, 156.66, 143.85, 141.20, 127.43, 126.85, 124.68, 119.55, 78.83, 67.84, 49.09, 48.66, 47.00, 38.27, 27.35.

#### 3.2.2. Synthesis of *N*-(((9*H*-fluoren-9-yl)methoxy)carbonyl)-*N*-(5-((*tert*-butoxycarbonyl)amino)propyl)glycine [Fmoc-NOrn(Boc)-OH, **18**]

Fmoc-NOrn(Boc)-OH (**18**) was prepared, analogously to the preparation of **17**, from *tert*-butyl (5-aminopropyl)carbamate (10.0 g). Crystallisation from EtOAc–heptane afforded building block **18** (18.6 g, 61%) as a white solid; t_R_ = 6.72 min. (gradient 30–100% B during 10 min). HRMS: calcd for C_25_H_30_N_2_O_6_ [M + Na]^+^ 477.19961, found 477.20298; ΔM = 7.1 ppm. ^1^H NMR (600 MHz, methanol-d_4_) δ 7.79 (m, 2H, ArH-Fmoc), 7.60 (m, 2H, ArH-Fmoc), 7.38 (q, *J* = 7.1 Hz, 2H, ArH-Fmoc), 7.31 (m, 2H, ArH-Fmoc), 4.44 (m, 2H, CH_2_-Fmoc), 4.22 (m, 1H, CH-Fmoc), 3.92 (d, *J* = 4.2 Hz, 2H, N-CH_2_-C=O)), 3.34, 3.10 (two m, 2H, N-CH_2_-CH_2_), 3.03, 2.82 (two m, 2H, CH_2_-CH_2_-CH_2_), 1.67 (p, *J* = 6.8 Hz, 2H, CH_2_-CH_2_-N), 1.41 (s, 9H, CH_3_-Boc). ^13^C NMR (151 MHz, methanol-d_4_) δ 171.63, 157.02, 156.63, 143.88, 141.25, 127.40, 126.83, 124.57, 119.55, 67.39, 48.58, 47.05, 45.94, 37.32, 27.83, 27.36.

#### 3.2.3. Synthesis of *N*-(((9*H*-fluoren-9-yl)methoxy)carbonyl)-*N*-(5-((*tert*-butoxycarbonyl)amino)butyl)glycine [Fmoc-NLys(Boc)-OH, **19**]

Fmoc-NLys(Boc)-OH (**19**) was prepared, analogously to to the preparation of **17**, from *tert*-butyl (5-aminobutyl)carbamate (24.9 g). Crystallisation from EtOAc–heptane afforded building block **19** (39.9 g, 43%) as a white solid; t_R_ = 6.94 min. (gradient 30–100% B during 10 min). HRMS: calcd for C_26_H_32_N_2_O_6_ [M + Na]^+^ 491.21526, found 491.21664; ΔM = 2.8 ppm. ^1^H NMR (600 MHz, methanol-*d*_4_) δ 7.80 (m, 2H, Ar*H*-Fmoc), 7.60 (m, 2H, Ar*H*-Fmoc), 7.38 (m, 2H, Ar*H*-Fmoc), 7.31 (m, 2H, Ar*H*-Fmoc), 4.46 (m, 2H, C*H*_2_-Fmoc), 4.22 (m, 1H, C*H*-Fmoc), 3.90 (m, 2H, N-C*H*_2_-C=O)), 3.35–3.27 (m, 2H, CH_2_-C*H*_2_-N), 3.04, 2.90 (two t, *J* = 6.8 Hz, 2H, N-C*H*_2_-CH_2_), 3.01, 1.19 (two m, 2H, N-CH_2_-C*H*_2_-CH_2_), 1.52, 1.45 (q, *J* = 7.4 Hz, 1H, CH_2_-C*H*_2_-CH_2_-N), 1.43 (s, 9H, C*H*_3_-Boc). ^13^C NMR (151 MHz, methanol-*d*_4_) δ 171.56, 156.75, 156.53, 143.83, 141.35, 127.44, 126.84, 124.70, 119.60, 78.46, 66.85, 48.08, 47.92, 47.82, 47.08, 39.54, 27.40, 26.66, 24.67.

#### 3.2.4. Synthesis of *N*-(((9*H*-fluoren-9-yl)methoxy)carbonyl)-*N*-(5-((*tert*-butoxycarbonyl)amino)pentyl)glycine [Fmoc-NhLys(Boc)-OH, **20**]

Fmoc-NhLys(Boc)-OH (**20**) was prepared, analogously to to the preparation of **17**, from tert-butyl (5-aminopentyl)carbamate (5.0 g). Crystallisation from EtOAc–heptane afforded building block **20** (5.4 g, 45%) as a white solid; t_R_ = 7.27 min. (gradient 30–100% B during 10 min). HRMS: calcd for C_27_H_34_N_2_O_6_ [M + Na]^+^ 505.23091, found 505.23220; ΔM = 2.6 ppm. ^1^H NMR (600 MHz, CDCl_3_) δ 7.74 (m, 2H, ArH-Fmoc), 7.55 (m, 2H, ArH-Fmoc), 7.37 (m, 2H, ArH-Fmoc), 7.29 (m, 2H, ArH-Fmoc), 4.48 (m, 2H, CH_2_-Fmoc), 4.21 (m, 1H, CH-Fmoc), 3.93 (m, 2H, N-CH_2_-C(O)), 3.31, 3.04 (two t, *J* = 7.3 Hz, 2H, CH_2_-CH_2_-N), 3.08 (q, *J* = 8.8, 8.2 Hz, 2H, N-CH_2_-CH_2_), 1.52 (p, *J* = 7.5 Hz, 1H, CH_2_-CH_2_-CH_2_-N), 1.46 (s, 9H, CH_3_-Boc), 1.36 (p, *J* = 7.4 Hz, 2H, N-CH_2_-CH_2_-CH_2_), 1.30 (p, *J* = 7.7 Hz, 2H, CH_2_-CH_2_-CH_2_-N), 1.27, 1.09 (two m, 2H, CH_2_-CH_2_-CH_2_-CH_2_-CH_2_). ^13^C NMR (151 MHz, CDCl_3_) δ 173.69, 156.79, 156.14, 144.05, 141.48, 127.79, 127.20, 124.98, 120.06, 79.54, 67.81, 48.82, 48.66, 47.40, 40.64, 29.71, 28.56, 27.76, 23.89.

#### 3.2.5. Synthesis of *N*-(((9*H*-fluoren-9-yl)methoxy)carbonyl)-*N*-benzylglycine [Fmoc-NPhe-OH, **21**]

Fmoc-NPhe-OH (**21**) was prepared, analogously to the preparation of **17**, from benzylamine (14.0 g). Crystallisation from EtOAc–heptane afforded building block **21** (24.5 g, 48.3%); t_R_ = 7.18 min. (gradient 30–100% B during 10 min) HRMS: calcd for C_24_H_21_NO_4_ [M + Na]^+^ 410.13628, found 410.13852; ΔM = 5.5 ppm. ^1^H NMR (600 MHz, methanol-d_4_) δ 7.76 (m, 2H, Ar*H*-Fmoc), 7.62 – 7.48 (m, 2H, Ar*H*-Ph), 7.37 (m, 2H, Ar*H*-Fmoc), 7.30 (m, 2H, Ar*H*-Fmoc), 7.28–7.24 (m, 2H, Ar*H*-Ph), 7.24–7.15 (m, 2H, Ar*H*-Fmoc), 7.00–6.96 (m, 1H, Ar*H*-Ph), 4.51, 4.33 (two s, 2H, N-C*H*_2_-C=O), 4.51 (m, 2H, Ar*H*-Fmoc), 4.22 (t, *J* = 6.1 Hz, 1H, C*H*-Fmoc), 3.87, 3.75 (two s, 2H, C*H*_2_-Ph). ^13^C NMR (151 MHz, methanol-d_4_) δ 171.28, 156.83, 143.80, 141.29, 136.84, 128.29, 127.53, 127.43, 127.37, 127.24, 127.13, 126.80, 126.79, 124.58, 119.56, 67.51, 50.91, 46.98, 46.85.

#### 3.2.6. Synthesis of *N*-(((9*H*-fluoren-9-yl)methoxy)carbonyl)-*N*-phenethylglycine [Fmoc-NhPhe-OH, **22**]

Phenethylamine (20.8 mL) was dissolved in THF (75 mL). Then Et_3_N (69 mL, 3.0 equiv) was added to the solution. *Tert*-butyl bromoacetate (32.2 g, 1 equiv) was dissolved in THF (75 mL) and added dropwise to the solution under stirring. The mixture was kept at rt for 16 h. The mixture was filtered, the filtrate was concentrated in vacuo, and the residue was redissolved in CH_2_Cl_2_ (50 mL). The solution was loaded onto a VLC column (height, 7.5 cm; diameter, 12 cm; column material: 15–40 µm silica gel, column pretreated with heptane). Gradient elution was carried out with heptane followed by CH_2_Cl_2_−MeOH 100:1 and CH_2_Cl_2_−MeOH 50:10. The appropriate fractions were concentrated in vacuo to yield the intermediate *tert*-butyl ester (31.8 g, 82%) as a yellow oil, which was dissolved in CH_2_Cl_2_ (120 mL), and then TFA (280 mL) was added slowly to the solution under stirring. The mixture was kept at rt for 4 h and then concentrated in vacuo. The residue was dissolved in toluene (100 mL) and concentrated in vacuo. This was repeated twice to give the crude acid (39.76 g) as a yellow solid, which was dissolved in dioxane (400 mL) and 10% Na_2_CO_3_ (800 mL). Then, Fmoc-Cl (72.6 g, 1.7 equiv) dissolved in dioxane (400 mL) was added dropwise under stirring at 0 °C. The mixture was then kept at rt for 16 h. The mixture was adjusted to pH 10 by adding 10% Na_2_CO_3_ (200 mL) and split into four portions (~450 mL). Each portion was diluted with H_2_O (500 mL), washed with Et_2_O (900 mL), and filtered. The filtrate was adjusted to pH 1 by adding concentrated HCl (20 mL), and then extracted with EtOAc (900 mL). The combined organic phases were concentrated in vacuo to yield the crude product as a solid (55.8 g, 85%) which was dissolved in CH_2_Cl_2_ (100 mL) and loaded onto a VLC column (height, 11.5 cm; diameter, 12.0 cm; column material: 15–40 µm silica gel, column pretreated with heptane). Gradient elution was carried out with heptane followed by heptane−EtOAc 4:1 and heptane−EtOAc 1:1. The appropriate fractions were concentrated in vacuo to yield building block **22** (40.6 g, 62%) as a white solid; t_R_ = 7.50 min (gradient 30-100% B during 10 min). HRMS: calcd for C_25_H_23_NO_3_ [M + Na]^+^ 424.15193, found 424.15548; ΔM = 8.4 ppm. ^1^H NMR (600 MHz, methanol-*d*_4_) δ 7.81 – 7.75 (m, 2H, Ar*H*-Fmoc), 7.59 (m, 2H, Ar*H*-Fmoc), 7.38 (m, 2H, Ar*H*-Fmoc), 7.31 (m, 2H, Ar*H*-Fmoc), 7.23 (m, 2H, Ar*H*-Ph), 7.19–7.13 (m, 2H, Ar*H*-Ph), 6.92–6.87 (m, 1H, Ar*H*-Ph), 4.47 (m, 2H, C*H*_2_-Fmoc), 4.18 (dd, *J* = 6.5, 5.6 Hz, 1H, C*H*-Fmoc), 3.78 (m, 2H, N-C*H*_2_-C=O), 3.35 (m, 2H, N-C*H*_2_-CH_2_), 2.62 (m, 2H, CH_2_-C*H*_2_-Ph). ^13^C NMR (151 MHz, methanol-*d*_4_) δ 171.59, 156.53, 143.93, 141.39, 138.81, 128.49, 128.07, 127.39, 126.81, 125.90, 124.50, 119.59, 66.54, 49.86, 48.65, 47.05, 33.96.

### 3.3. General Protocol for Manual Synthesis of Peptidomimetics

Peptidomimetics **4**–**10**, **12**, **13**, and **16** were prepared manually as previously described [38]. In brief, H-Rink-Amide ChemMatrix resin (PCAS BioMatrix Inc., Saint-Jean-sur-Richelieu, QC, Canada; loading 0.52 mmol/g, 0.05 mmol) and Teflon vessels (10 mL; fitted with a polypropylene filter) were used. Coupling conditions used for Fmoc-protected amino acid and peptoid building blocks: 3.0 equiv building block, 3.0 equiv *N*,*N*′-diisopropylcarbodiimide (DIC), and 3.0 equiv ethyl (hydroxyimino)cyanoacetate (OxymaPure^®^) (>1 h under shaking at 40 °C; amino acid building blocks were coupled twice to ensure complete conversion of resin-bound peptoid secondary amines). Fmoc deprotection conditions: 20% piperidine in DMF (2 × 10 min, each time with 5 mL under shaking at 40 °C). Washing conditions: DMF, MeOH, and CH_2_Cl_2_ (3 × 3 min, each time with 5 mL under shaking at 40 °C). Capping was applied after loading, and after all couplings with amino acid building blocks (except the last one): Ac_2_O–DIPEA–NMP 1:2:3 (5 mL for 10 min under shaking at 40 °C). Cleavage and side-chain deprotection were performed with TFA–H_2_O–CH_2_Cl_2_ (95:2.5:2.5; 2 × 1 h, each with 5 mL under shaking at rt). The filtrates were collected, and the resin was further eluted with TFA (2 mL) and CH_2_Cl_2_ (2 mL). The combined filtrates were concentrated in vacuo and co-evaporated with toluene (3 × 5 mL). The crude product was purified by preparative HPLC, and the appropriate fractions were concentrated in vacuo and lyophilised. Identity was verified by HRMS, and purity (>97%) was determined by analytical UHPLC.

### 3.4. General Protocol for Microwave-Assisted Automated Synthesis of Peptidomimetics

Peptidomimetics **1**–**3**, **11**, **14**, and **15** were prepared by automated microwave (MW)-assisted Fmoc-based SPPS on a CEM™ Liberty microwave peptide synthesiser. H-Rink-Amide ChemMatrix^®^ resin (PCAS Bio-Matrix Inc., Saint-Jean-sur-Richelieu, QC, Canada; loading 0.52 mmolg^−1^, 0.1 mmol) was used. Fmoc (9-fluorenylmethyloxycarbonyl) deprotection conditions: excess 20% piperidine in DMF, initially 75 °C (MW) for 30 s, subsequently 75 °C (MW) for 180 s. Coupling conditions: 5.0 equiv of building block, 5.0 equiv DIC, and 5.0 equiv OxymaPure^®^, DMF, 75 °C (MW) for 15 min. The resin was transferred to a Teflon vessel fitted with a polypropylene filter by using DMF and CH_2_Cl_2_. Upon draining, the resin was washed with DMF, MeOH, and CH_2_Cl_2_ (3 × 3 min, each time with 5 mL under shaking at rt). Cleavage and side-chain deprotection followed by purification were performed as described above for manual synthesis of peptidomimetics.

### 3.5. Determination of Minimum Inhibitory Concentration

MIC values were evaluated by using the modified Hancock lab protocol [71] in non-binding polystyrene microtitre plates, with bacterial suspensions of ∼2 × 10^5^ CFU/mL in Mueller-Hinton broth (Difco, Sparks MD, USA), with Mg^2+^ and Ca^2+^ concentrations of 4 mg/L each. The compounds were dissolved in H_2_O and diluted in 0.01% acetic acid, 0.2% BSA (final concentration). Aliquots of 11 µL of 10 × test compounds were then transferred to the appropriate wells with bacterial suspensions. For preparation and transfer of the solutions of the compounds, low-binding sterile tubes and tips (Axygen, Union City CA, USA) were used. After 20 h incubation of covered plates at 35 °C (±2 °C) with circular shaking at 220 rpm, the MICs were read. The antibacterial activity of peptidomimetics was tested against *E. coli* ATCC 25922, *K. pneumoniae* ATCC 13883, *P. aeruginosa* ATCC 27853, *P. aeruginosa* PAO1, *A. baumannii* ATCC 19606, *S. aureus* ATCC 29213, and *E. faecalis* ATCC 29212.

### 3.6. Determination of Hemolytic Activity

The lysis of human red blood cells was measured as previously described [15], with modifications. In brief, freshly drawn human red blood cells (hRBCs) were washed three times with PBS buffer and centrifuged two times for 5 min at 2500 rpm. A two-fold serial dilution of peptidomimetics in PBS buffer was added to each well in a sterile polypropylene V-bottom 96-well plate to the total volume of 75 μL. A 0.5% *v/v* hRBC suspension (75 μL in PBS buffer) was added to each well to reach a final volume of 150 μL in each well. The plate was incubated (37 °C) for 60 min, and then the cells were subsequently pelleted by centrifugation at 1000 rpm for 5 min. The supernatants (50 μL) were transferred to clear, flat-bottomed plastic 96-well plates. The concentration of hemoglobin was detected by measuring the OD at 405 nm. The OD of cells incubated with 0.1% SDS defined 100% hemolysis, while the OD of cells incubated with PBS buffer defined 0% hemolysis. The concentrations tested were 200, 400, and 800 μg/mL (only average values for the highest concentration are stated in Table 2).

### 3.7. Determination of Antiproliferative Activity on HepG2 Cell Line

Side effects affecting cell viability were estimated on the HepG2 cell line ATCC HB-8065. In brief, HepG2 cells were seeded in flat-bottomed 96-well plates at a concentration of 5000 cells per well. At 80−90% confluency, the cells were incubated for 24 h in a humidified incubator (5% CO_2_, 37 °C). The medium was removed, and the cells were incubated for 48 h in a humidified incubator (5% CO_2_, 37 °C) with compounds in two-fold serial dilutions. An MTT assay was then performed as previously described [72]. In brief, after incubation with the test compounds, the culture medium was removed and fresh medium with 0.2 mg/mL MTT was added in each well of the plate. After incubation (3 h, 37 °C, 5% CO_2_), the medium with MTT was removed, and 200 μL dimethyl sulfoxide were added at once to each sample. Absorbance of MTT was measured using spectrophotometer TECAN Infinite M1000 at 540 nm. The relative viability was calculated by using the formula: OD_treated cells_ × 100/OD_control cells_. The IC_50_ values are calculated by using the program Graph Pad Prism^®^ 5.0. For all compounds, the test range was 10–1280 μg/mL.

## 4. Conclusions

In the present work, we explored how the hydrophobicity and activity profiles (i.e., antibacterial activity versus effect on viability of mammalian cells) of a series of peptide/peptoid hybrid peptidomimetics depend on the structural features termed “relative side-chain length” and “nature of the more flexible residue” (i.e., whether the peptoid units are cationic or hydrophobic).

A general trend within each subgroup was that increased cationic side-chain length confers gradually reduced hydrophobicity, since the amino functionalities become increasingly more accessible to the surroundings due to a lowered shielding by the aromatic groups of the hydrophobic residues. This effect was most distinct for analogues displaying the short cationic Dab or NDab residues, as this feature conferred the highest hydrophobicity within each subgroup. However, extension of the cationic side-chain length beyond that of lysine resulted in a slightly increased hydrophobicity in accordance with an expected hydrophobicity enhancement resulting from the incorporation of additional methylene groups in the cationic side chains, thereby increasing the carbon content relative to the nitrogen/oxygen content.

The peptidomimetics were tested against a panel of seven bacterial strains. None of the peptidomimetics displayed significant activity against *K. pneumoniae*, and only four displayed activity against *A. baumannii*, while only a few analogues exhibited moderate to low activity against *S. aureus* and *E. faecalis*, respectively. By contrast, all analogues were active against *E. coli*, with the two least hydrophobic peptidomimetics being the least active, indicating the existence of a hydrophobicity threshold for optimal activity against *E. coli*. Otherwise, no clear design rules were identified except for the observation that in two of four subgroups the most hydrophobic member was slightly more potent. Most analogues were active against *P. aeruginosa*, and generally a shortening of the cationic side chains by one methylene group gave rise to a two-fold increased potency. This infers that peptidomimetics displaying a hydrophobic surface, comprised of the aromatic groups (partially) shielding the shorter cationic side chains, display more efficient membrane-disruptive interaction with *P. aeruginosa*.

Testing for hemolytic properties as well as effect on HepG2 cell viability revealed that peptidomimetics displaying the above-mentioned hydrophobic surface proved to be the most hemolytic and affect HepG2 cell viability to the highest degree. Expectedly, high hydrophobicity was clearly correlated to increased effect on HepG2 cell viability, thereby corroborating that for a series of closely related compounds a threshold for acceptable maximal hydrophobicity may typically be identified. Consequently, it is important to: (i) ensure that the surface does not become too hydrophobic (i.e., considerably shorter cationic side chains as compared to the length of the hydrophobic side chains), and (ii) keep the overall hydrophobicity below the threshold that confers extensive toxicity toward mammalian cells regardless of the effects caused by relative side-chain length.

## Figures and Tables

**Table 1:** Minimal inhibitory concentrations (MICs) for peptidomimetics **1**–**16**.

Subgroup	Cmpd	Hydrophobicity (% MeCN at elution ^a^)	MIC (µg/mL)						
			Gram-Negative					Gram-Positive	
			*E. coli* ATCC 25922	*K. pneumoniae* ATCC 13883	*P. aeruginosa* ATCC 27853	*P. aeruginosa* PAO1	*A. baumannii* ATCC 19606	*S. aureus* ATCC 29213	*E. faecalis* ATCC 29212
**I**	**1**	37.5%	4-8	64	64	>64	>64	>64	>64
**I**	**2**	35.7%	8-16	64	>64	>64	>64	>64	>64
**I**	**3**	35.6%	8-16	32	>64	>64	>64	>64	>64
**I**	**4**	36.6%	4-8	64	>64	>64	64	>64	>64
**II**	**5**	43.5%	4	64	4	16	>64	8	32
**II**	**6**	41.2%	4	>64	8	16-32	>64	16	>64
**II**	**7**	40.6%	2-4	>64	32	32-64	>64	>64	>64
**II**	**8**	40.9%	2	64	64	64	4	>64	>64
**III**	**9**	38.0%	2-4	32-64	1	2	16-32	>64	>64
**III**	**10**	37.2%	4-8	>64	2-4	4-8	64->64	>64	>64
**III**	**11**	36.9%	4-8	64	4	8-16	>64	>64	>64
**III**	**12**	38.0%	2	>64	64	64	16	>64	>64
**IV**	**13**	41.4%	4	32	2	2-4	>64	>64	>64
**IV**	**14**	40.5%	4-8	32	8	8-16	>64	>64	>64
**IV**	**15**	40.5%	4	>64	16	8-16	64	>64	>64
**IV**	**16**	41.1%	4	>64	16	32	8	>64	>64
	**Colistin**		0.125-0.25	0.25	0.125-0.25	0.5	0.5	>64	>64

^a^ RP-HPLC gradient: 0 → 60% B (during 10 min). MeCN: acetonitrile.

**Table 2:** Effects of peptidomimetics on mammalian cell viability.

Subgroup	Cmpd	Hydrophobicity (% MeCN at elution ^a^)	IC_50_ (µg/mL) ^b^	Viability at 1280 µg/mL ^c^	Hemolytic Activity ^d^	Cell Selectivity ^e^
**I**	**1**	37.5%	1280	49%	7%	160-320
**I**	**2**	35.7%	>1280	62%	5%	>80
**I**	**3**	35.6%	1280	52%	8%	80-160
**I**	**4**	36.6%	1280	49%	9%	160-320
**II**	**5**	43.5%	307	-	43%	77
**II**	**6**	41.2%	456	-	8%	114
**II**	**7**	40.6%	720	-	6%	180-360
**II**	**8**	40.9%	266	-	9%	133
**III**	**9**	38.0%	326	-	79%	82-163
**III**	**10**	37.2%	923	-	10%	115-231
**III**	**11**	36.9%	>1280	76%	10%	>160
**III**	**12**	38.0%	1280	47%	9%	640
**IV**	**13**	41.4%	261	-	53%	65
**IV**	**14**	40.5%	684	-	11%	86-171
**IV**	**15**	40.5%	595	-	8%	149
**IV**	**16**	41.1%	410	-	9%	103

^a^ RP-HPLC gradient: 0 → 60% B (during 10 min). ^b^ Toxicity against HepG2 cells is given as IC_50_ value for inhibiting growth of HepG2 cells (assay was performed in two biological replicates each with three technical replicates). The highest concentration tested was 1280 μg/mL. ^c^ The viability of HepG2 cells at 1280 µg/mL is only given for compounds with an IC_50_ value of 1280 µg/mL or higher. ^d^ Percentage hemolysis in human red blood cells (RBCs) at 800 μg/mL. ^e^ Cell selectivity was calculated as the ratio between IC_50_ for effect on HepG2 cell viability and MIC against *E. coli*.

## Supplementary Materials

The following are available online, correlations between cationic side-chain length, hydrophobicity, and MIC or IC_50_, HRMS spectra, HPLC chromatograms for all peptidomimetics and building blocks, and HepG2 cell viability curves for peptidomimetics.
