# Supplementary material for: Peptide/Peptoid Hybrid Oligomers: The Influence of Hydrophobicity and Relative Side-Chain Length on Antibacterial Activity and Cell Selectivity
Source: Molecules. 2019 Dec 4;24(24):4429. doi: 10.3390/molecules24244429 (PMC6943742; doi:10.3390/molecules24244429)

*Supplementary Materials for*

**Peptide/Peptoid Hybrid Oligomers: Influence of Hydrophobicity and Relative Side chain Length on Antibacterial Activity and Cell Selectivity**

**Nicki Frederiksen, Paul R. Hansen, Fredrik Björkling, and Henrik Franzyk ^*^**

Department of Drug Design and Pharmacology, Faculty of Health and Medical Sciences, University of Copenhagen, Jagtvej 162, DK-2100 Copenhagen, Denmark; nicki.frederiksen@sund.ku.dk

* Correspondence: henrik.franzyk@sund.ku.dk; +45-3533-6255

Contents

[Graphs of %MeCN at RP-HPLC elution versus MIC or IC_50_ 2](#_Toc25066807)

[Chromatograms of a dilution series of peptidomimetic 5 5](#_Toc25066808)

[Characterisation of peptidomimetics 6](#_Toc25066809)

[Characterisation of peptoid building blocks 22](#_Toc25066810)

# Graphs of %MeCN at RP-HPLC elution versus MIC or IC_50_


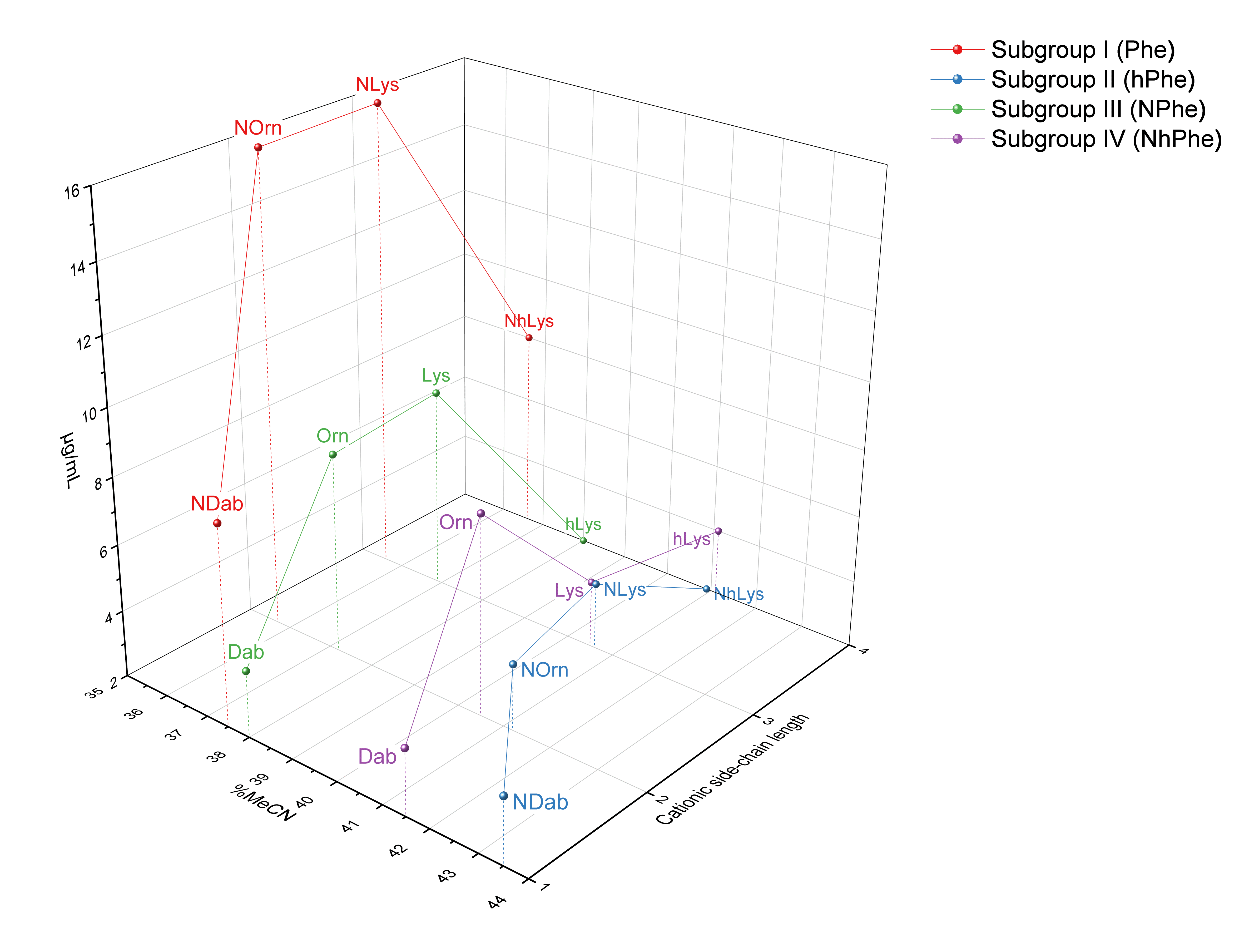


**Figure S1:** Cationic side-chain length versus %MeCN at RP-HPLC elution versus MIC against E. coli.


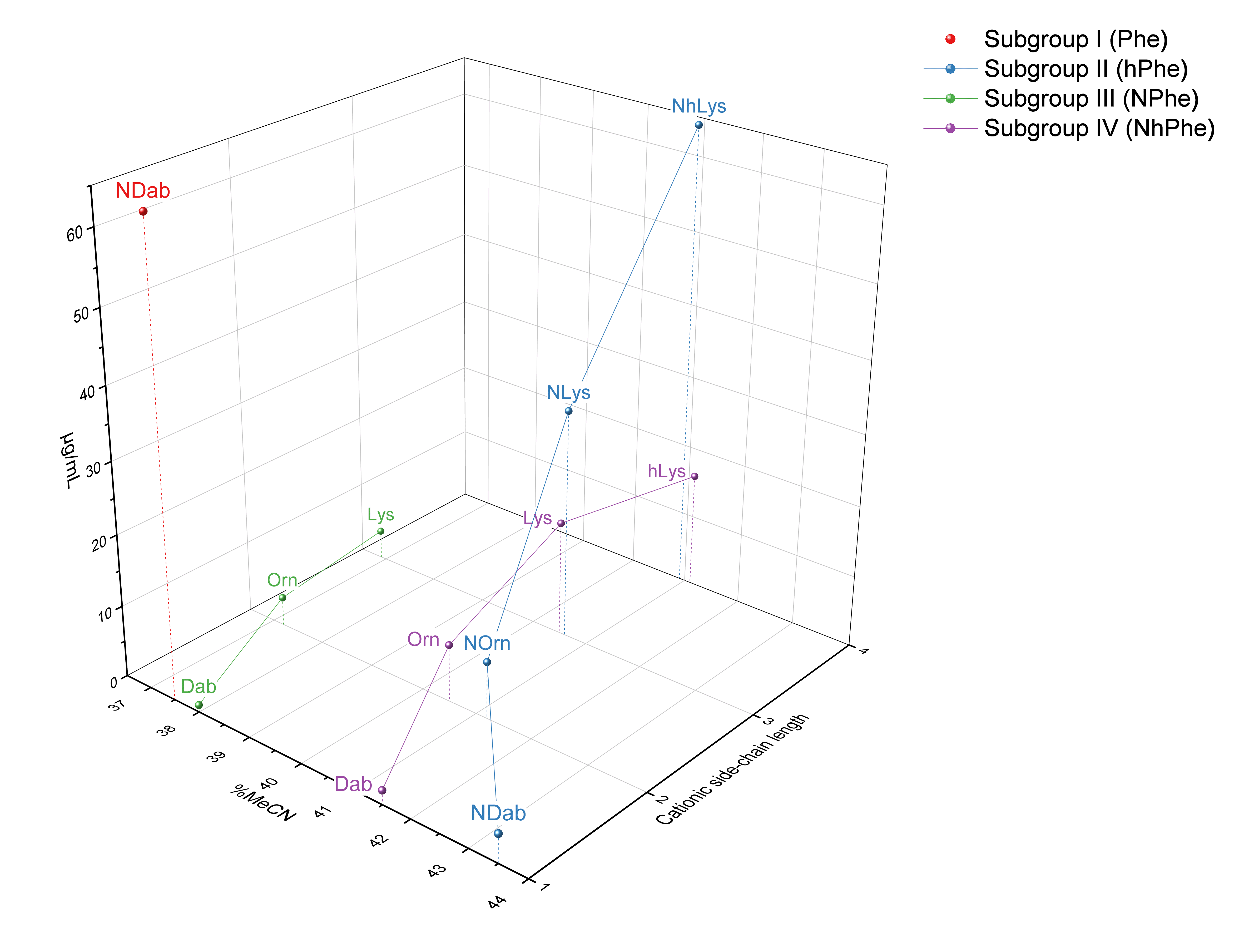


**Figure S2:** Cationic side-chain length versus %MeCN at RP-HPLC elution versus MIC against P. aeruginosa.


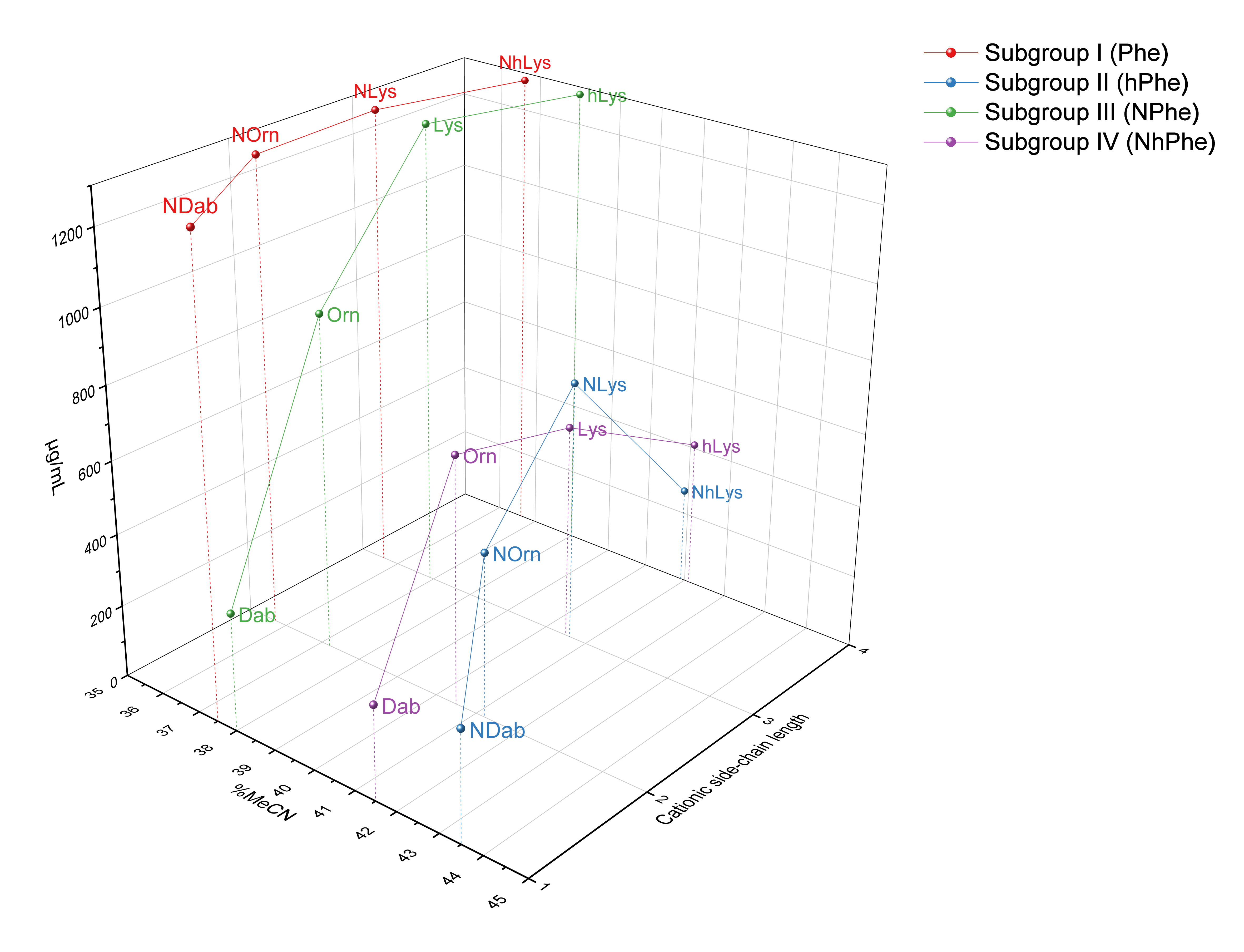


**Figure S3:** Cationic side-chain length versus %MeCN at RP-HPLC elution versus HepG2 IC_50_.

# Chromatograms of a dilution series of peptidomimetic 5

Peptidomimetic **5** was dissolved in eluent A to yield a solution with a concentration of 4 mg/mL. A dilution series consisting of 2-fold dilutions to a concentration of 0.03125 mg/mL was then produced and analysed using the same conditions as stated in Materials and Methods. Furthermore, the sample containing the highest concentration was analysed a second time following sonication for 10 min. and heating to 50 °C for 10 min. No difference was observed following this procedure.


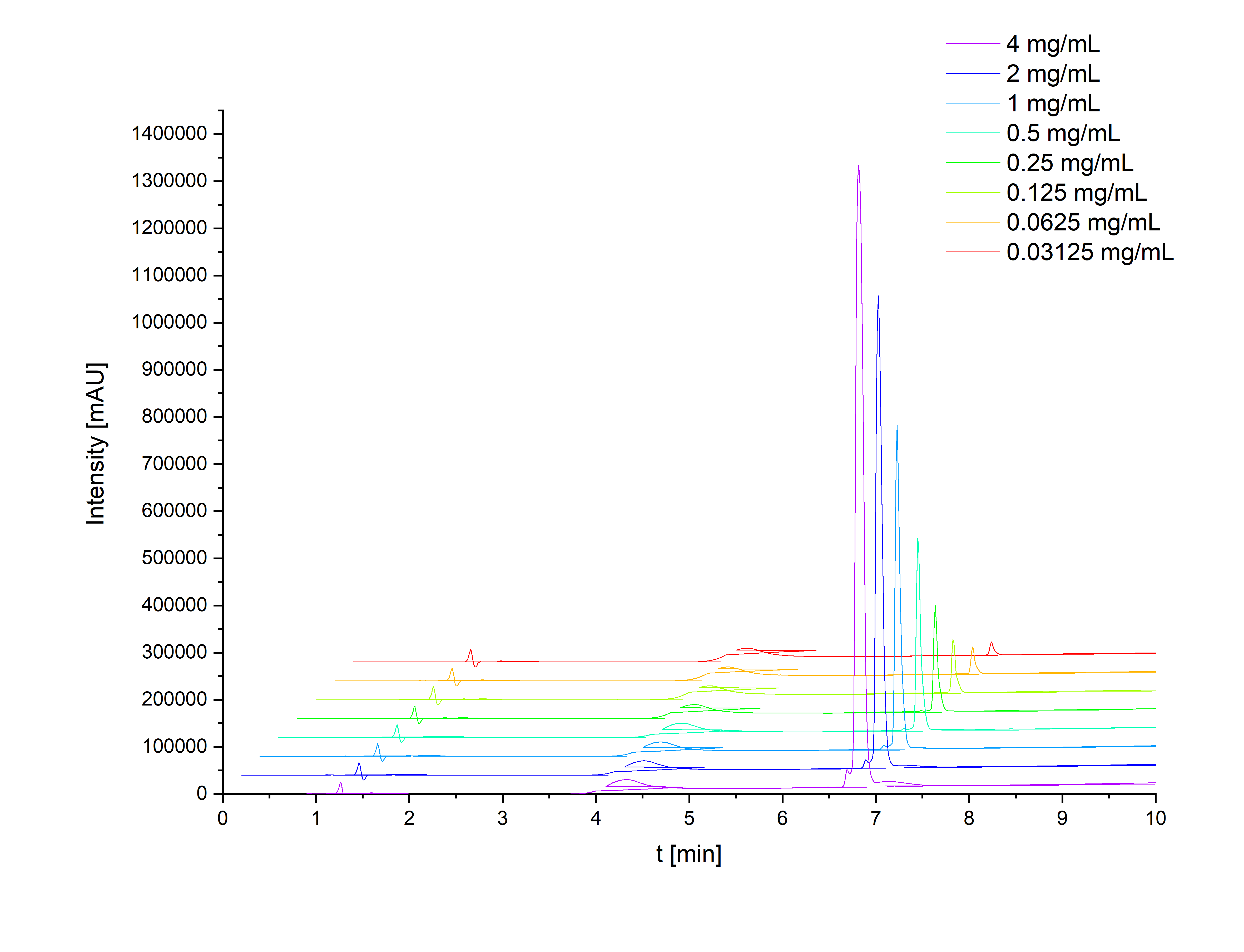


**Figure S4:** Chromatograms of peptidomimetic **5** dilution series using a Phenomenex Luna® 2.5 µm C18(2) 100 Å LC Column (100 x 3 mm) with a gradient of 0% to 60% B during 10 min. with UV detection at λ = 220 nm.


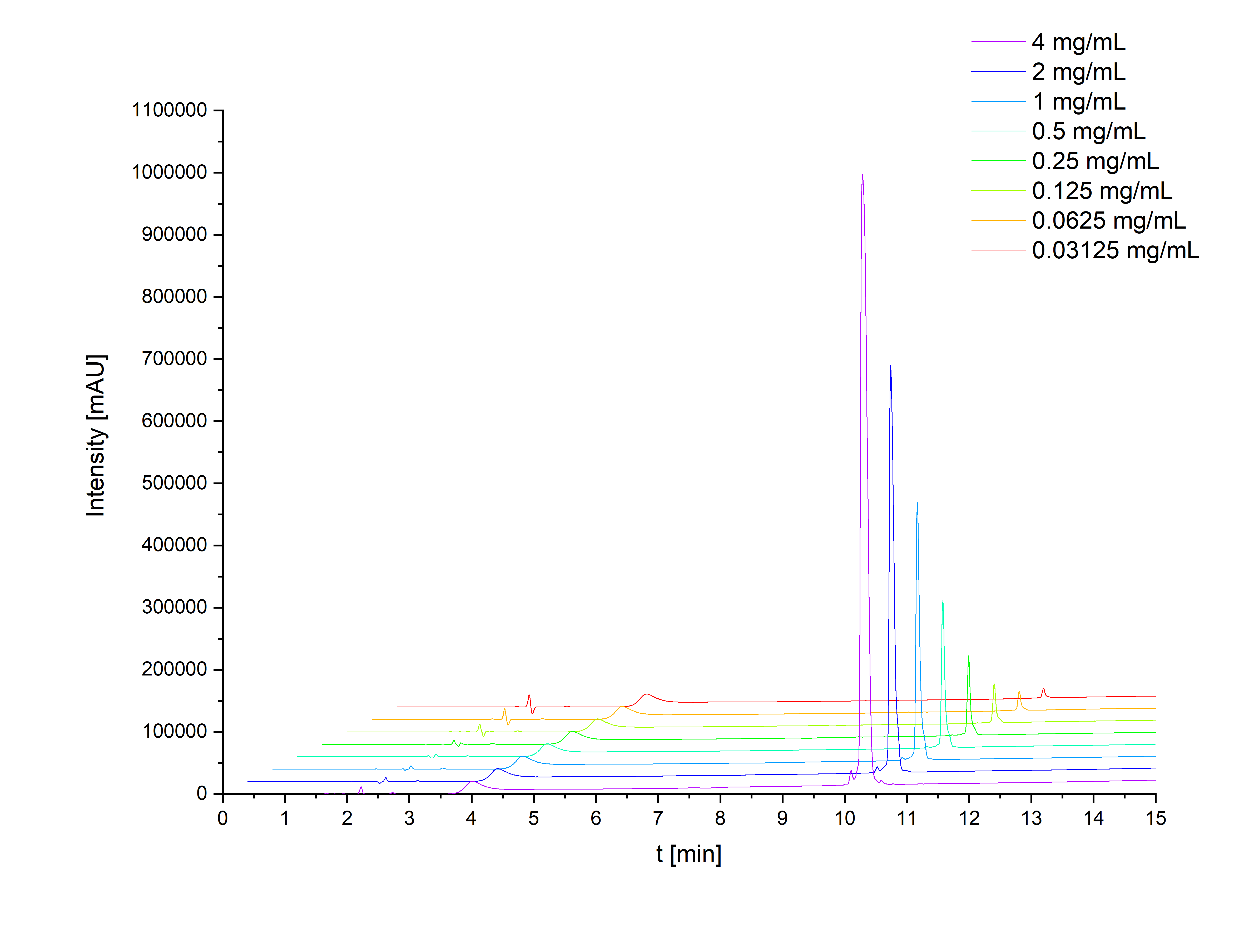


**Figure S5:** Chromatograms of peptidomimetic 5 dilution series using a Phenomenex Aeris™ 3.6 µm PEPTIDE XB-C18 100 Å LC Column (150 x 4.6 mm) with a gradient of 0% to 60% B during 15 min. with UV detection at λ = 220 nm.

# Characterisation of peptidomimetics

| **Peptidomimetic 1** | |
| --- | --- |
| **HRMS:** calculated for [M+1H]^1+^ 1501.82964, found 1501.83306; ∆M = 2.3 ppm.  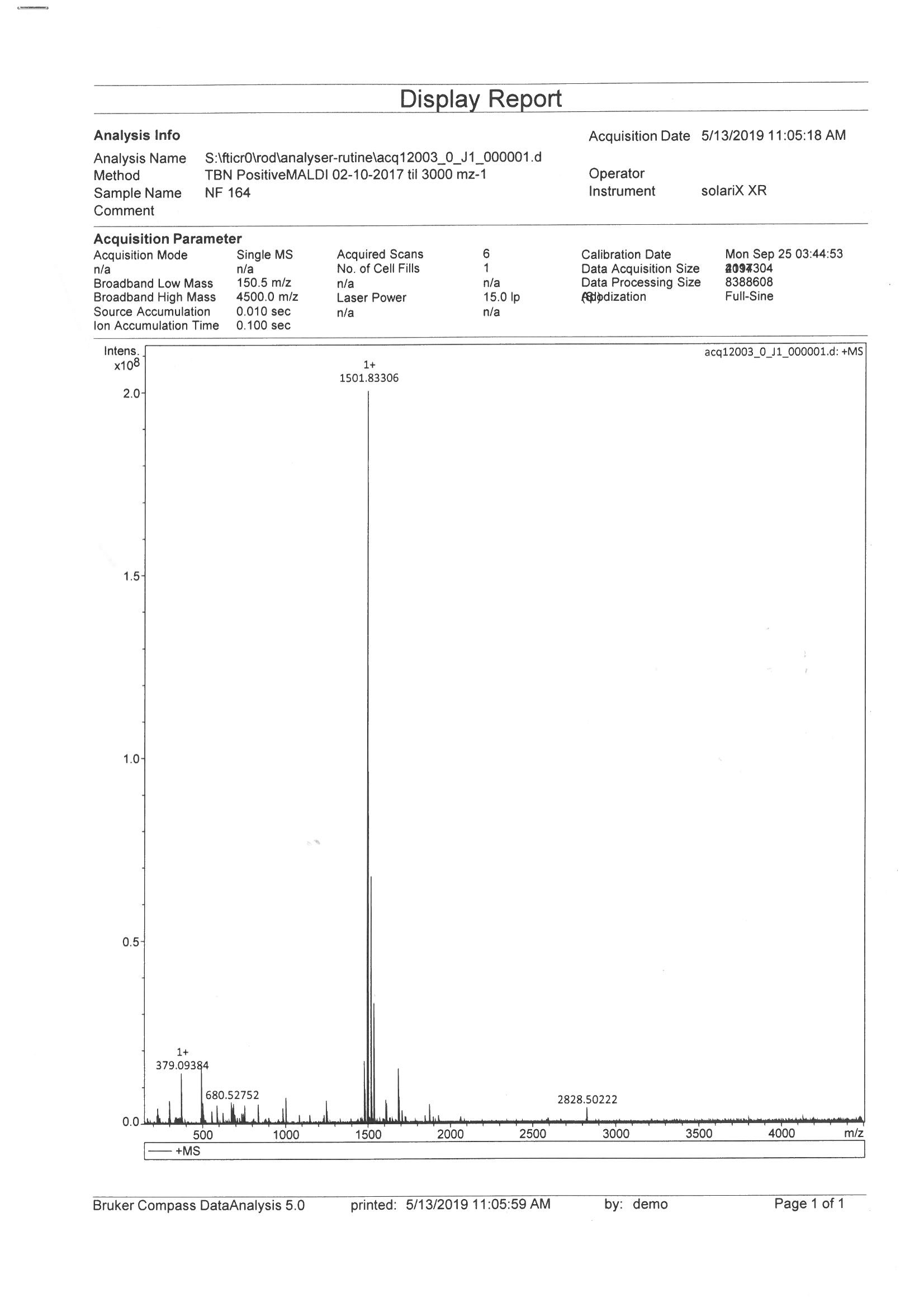 | |
| **HPLC:** Charge: +7. t_R_ = 6.02 min, purity 98.4%. Gradient: 0-60% B during 10 min.  B = 95% MeCN + 0.1% TFA. 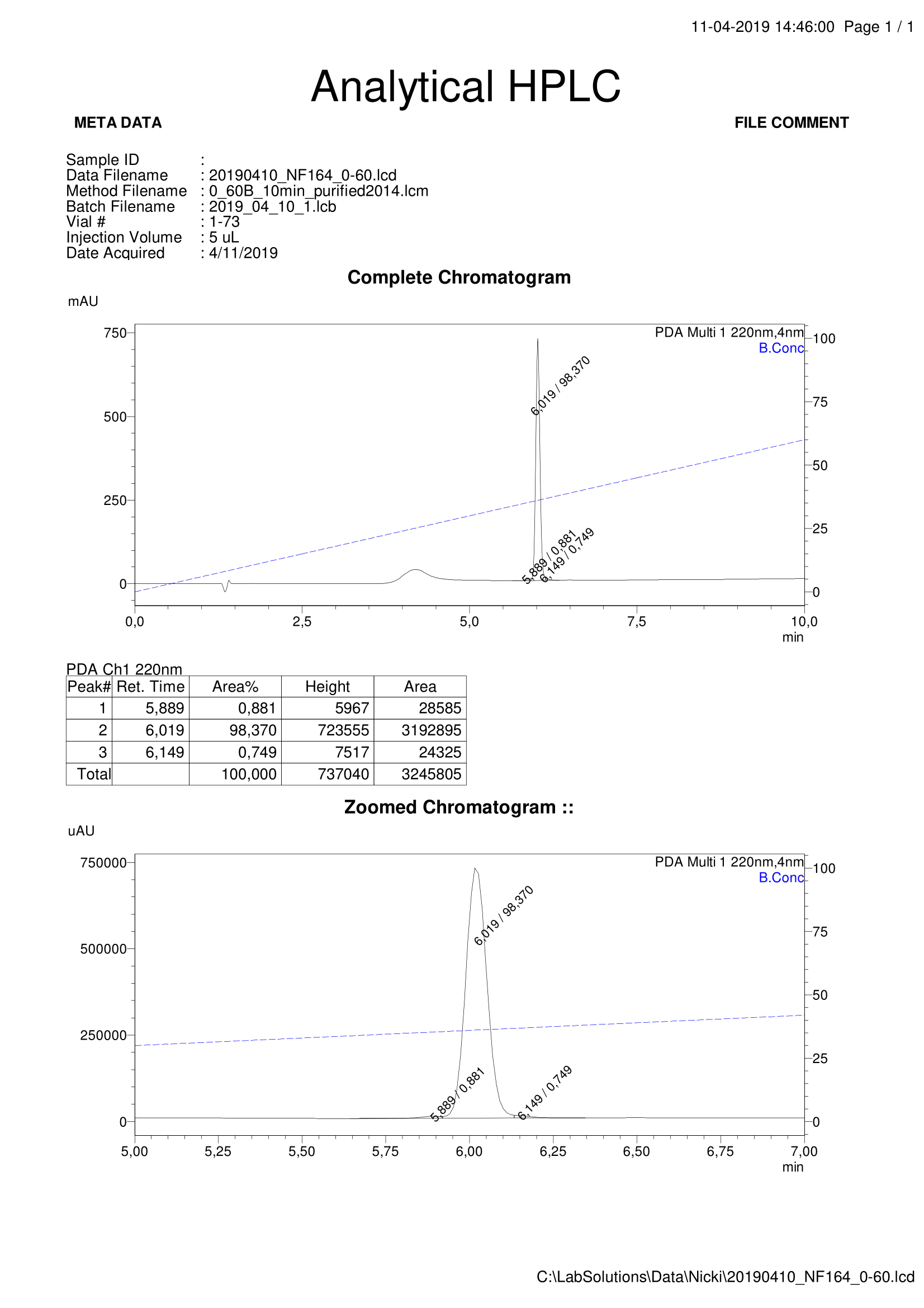 | **HepG2 cell viability**   |

| **Peptidomimetic 2** | |
| --- | --- |
| **HRMS:** calculated for [M+1H]^1+^ 1585.92354, found 1585.93652; ∆M = 8.2 ppm.  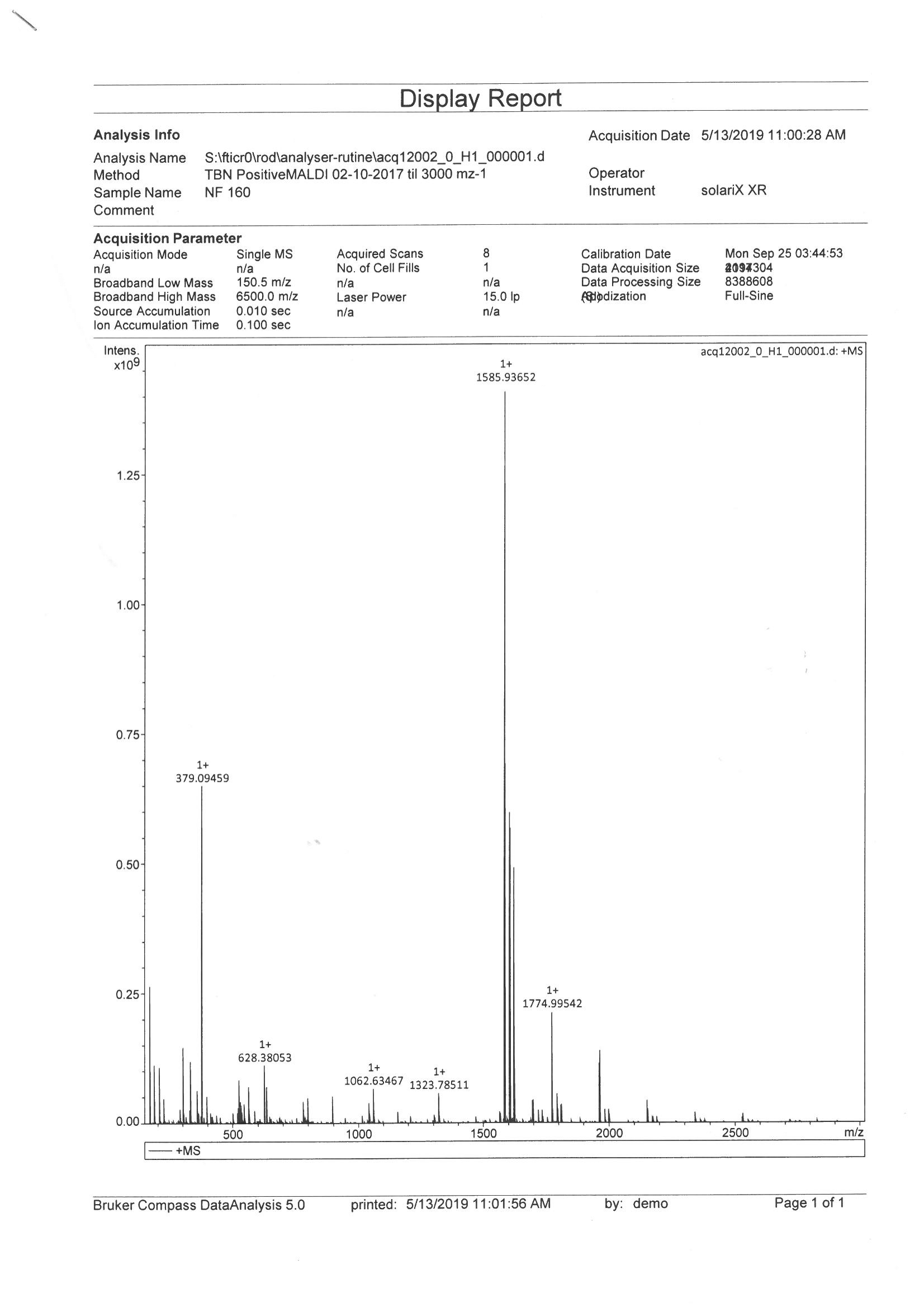 | |
| **HPLC:** Charge: +7. t_R_ = 5.69 min, purity 100.0%. Gradient: 0-60% B during 10 min.  B = 95% MeCN + 0.1% TFA.  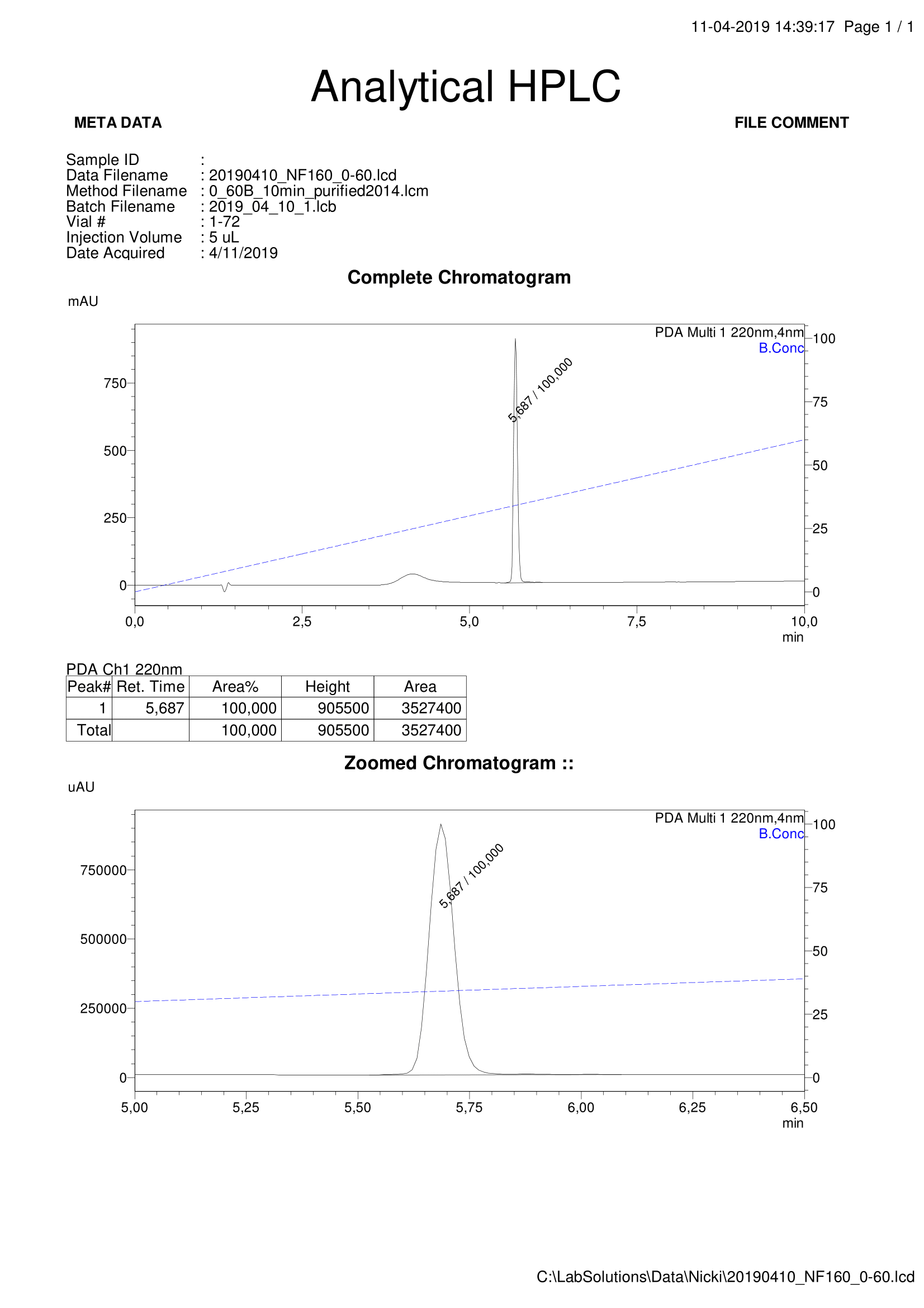 |  |

| **Peptidomimetic 3** | |
| --- | --- |
| **HRMS:** calculated for [M+1H]^1+^ 1670.01744, found 1670.01698; ∆M = 0.3 ppm.  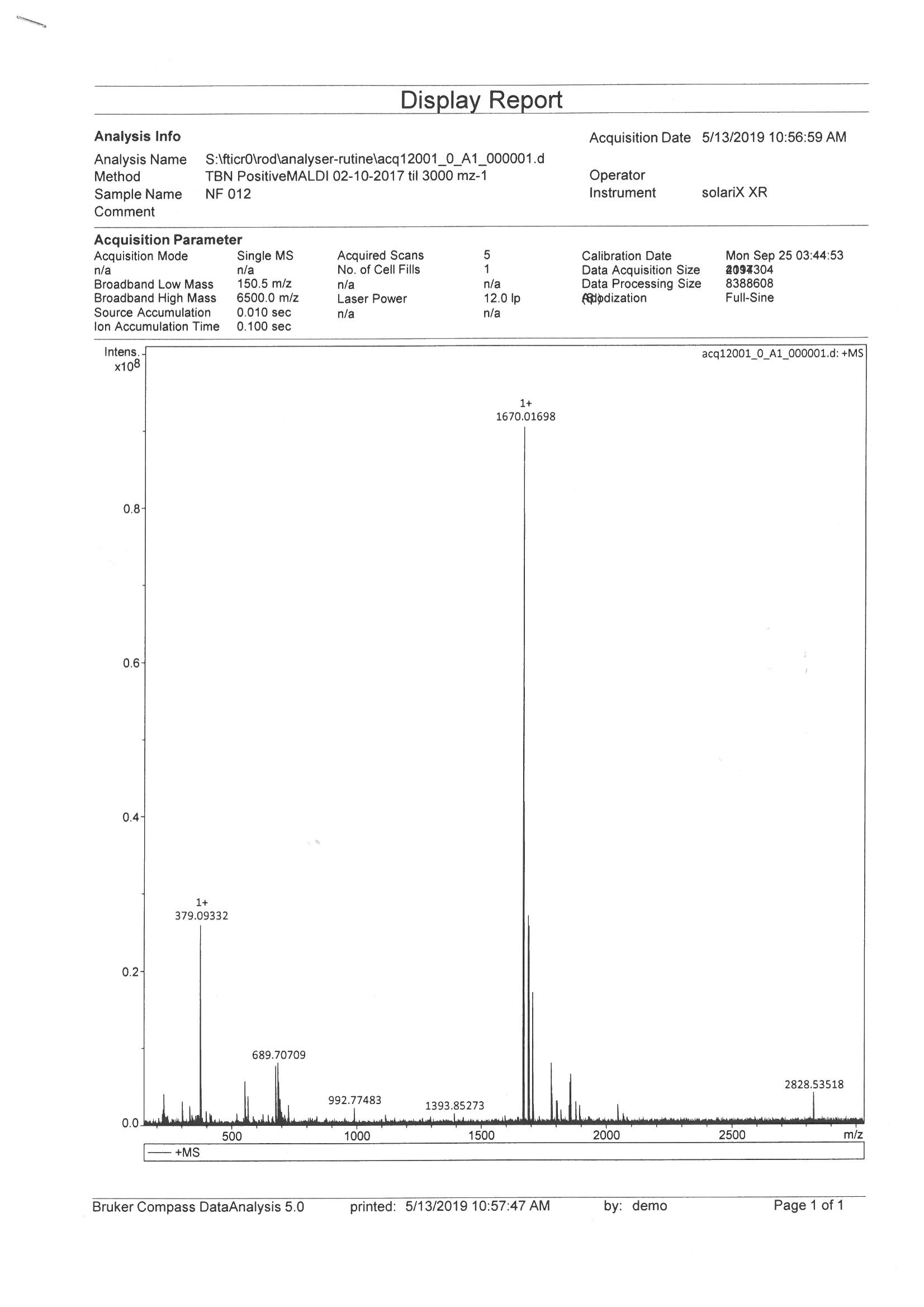 | |
| **HPLC:** Charge: +7. t_R_ = 5.66 min, purity 97.65%. Gradient: 0-60% B during 10 min.  B = 95% MeCN + 0.1% TFA.  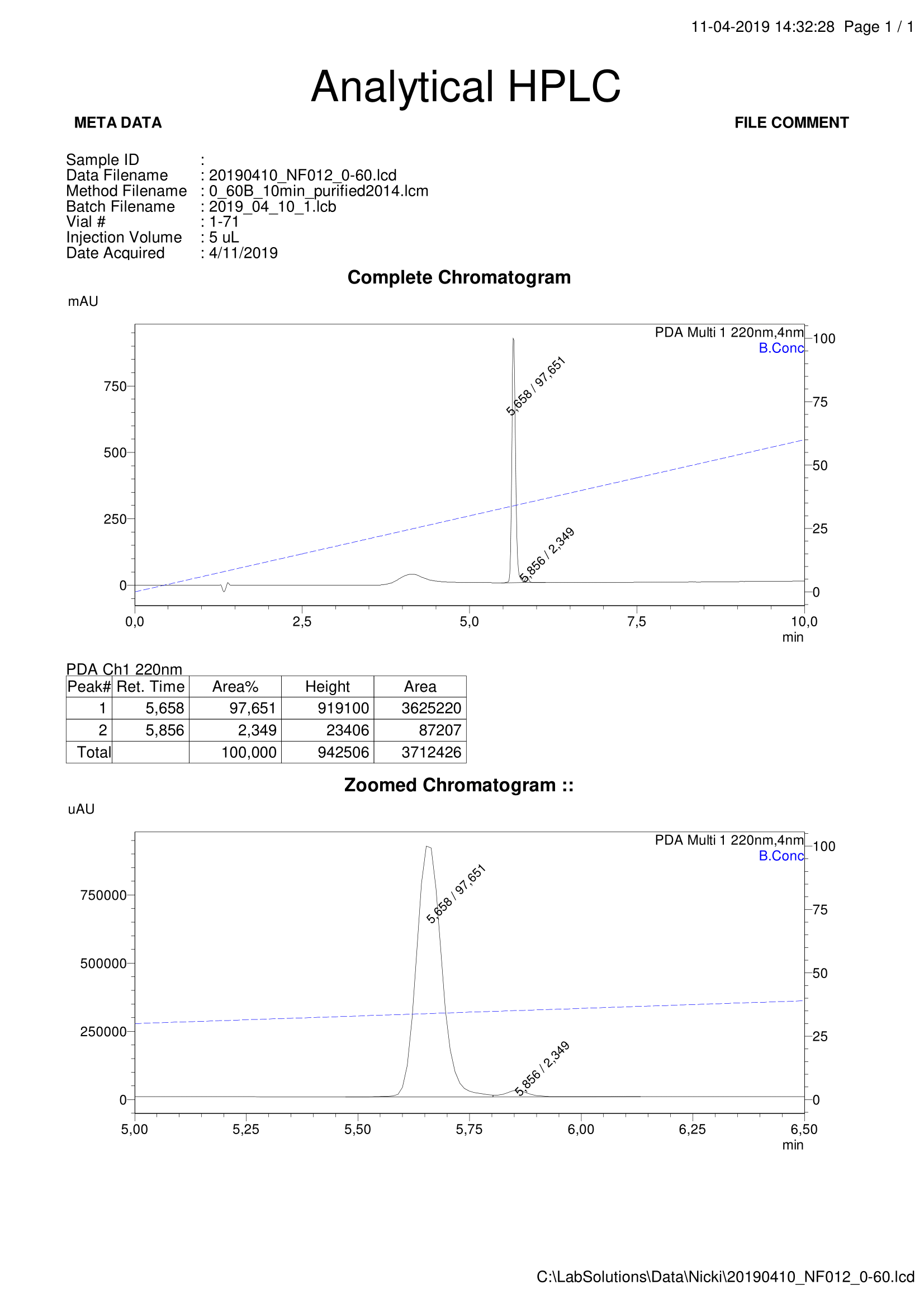 | **HepG2 cell viability**   |

| **Peptidomimetic 4** | |
| --- | --- |
| **HRMS:** calculated for [M+1H]^1+^ 1754.11134, found 1754.10850; ∆M = 1.6 ppm.  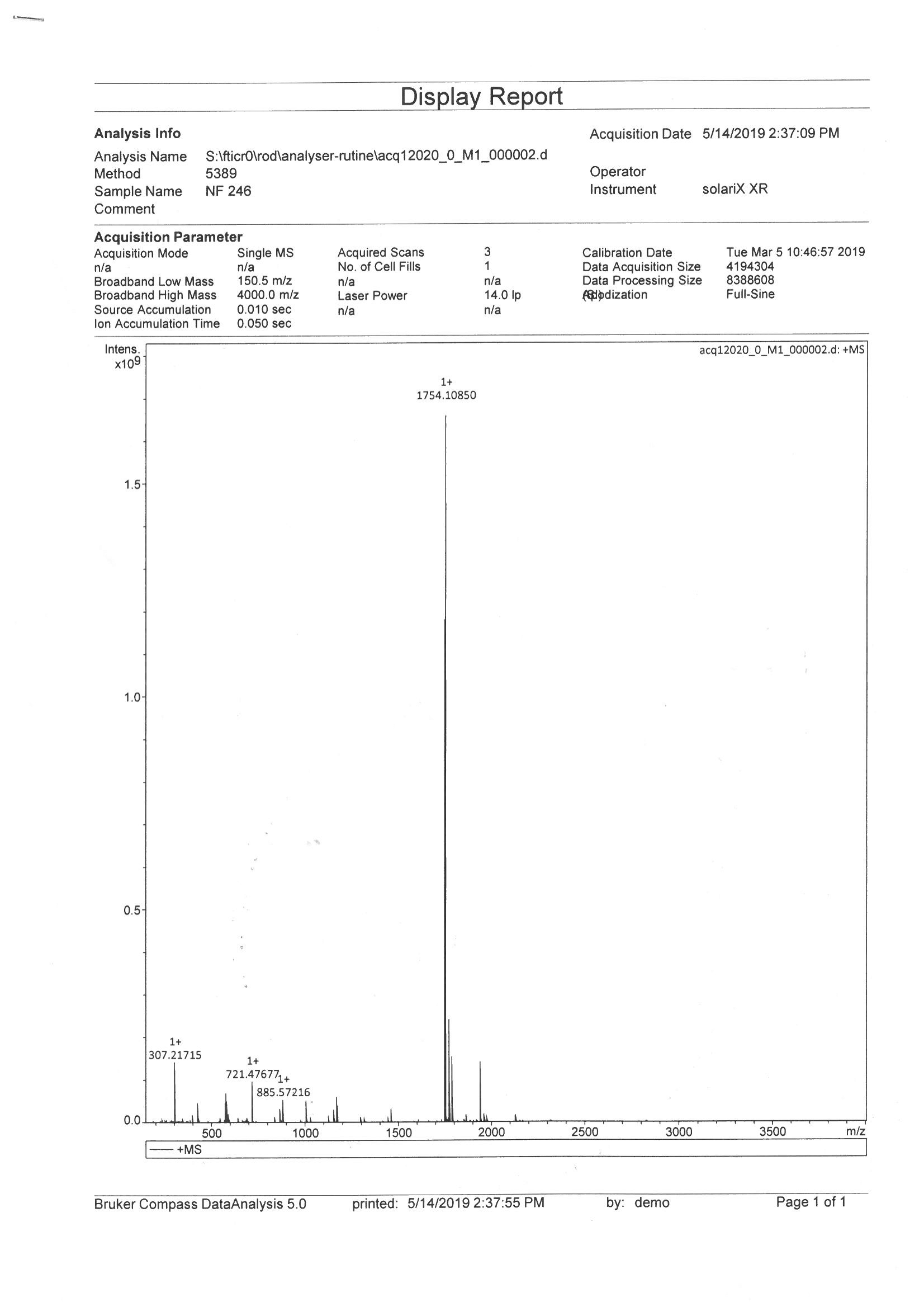 | |
| **HPLC:** Charge: +7. t_R_ = 5.86 min, purity 99.39%. Gradient: 0-60% B during 10 min.  B = 95% MeCN + 0.1% TFA. 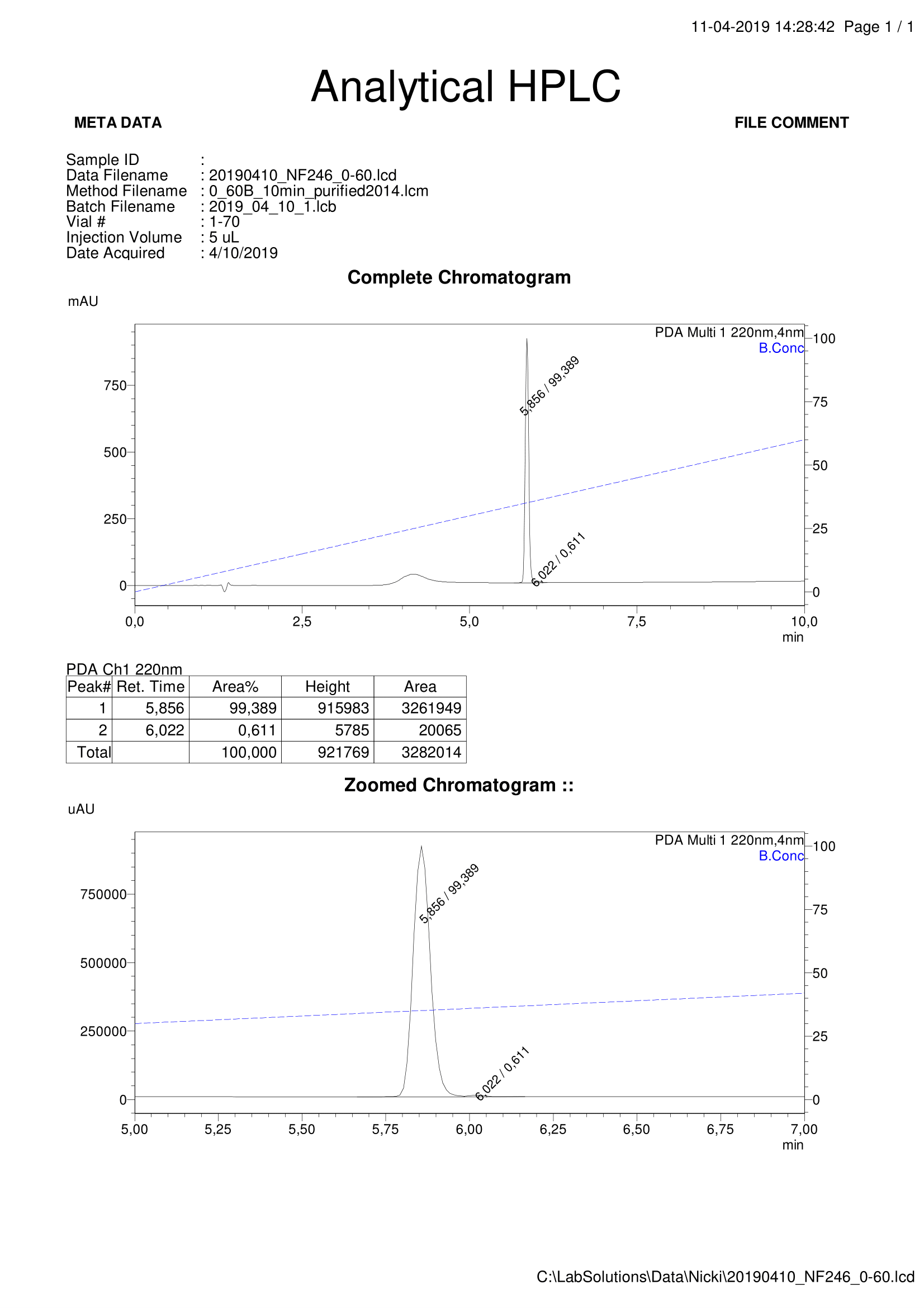 | **HepG2 cell viability**   |

| **Peptidomimetic 5** | |
| --- | --- |
| **HRMS:** calculated for [M+1H]^1+^ 1585.92354, found 1585.92273; ∆M = 0.5 ppm.  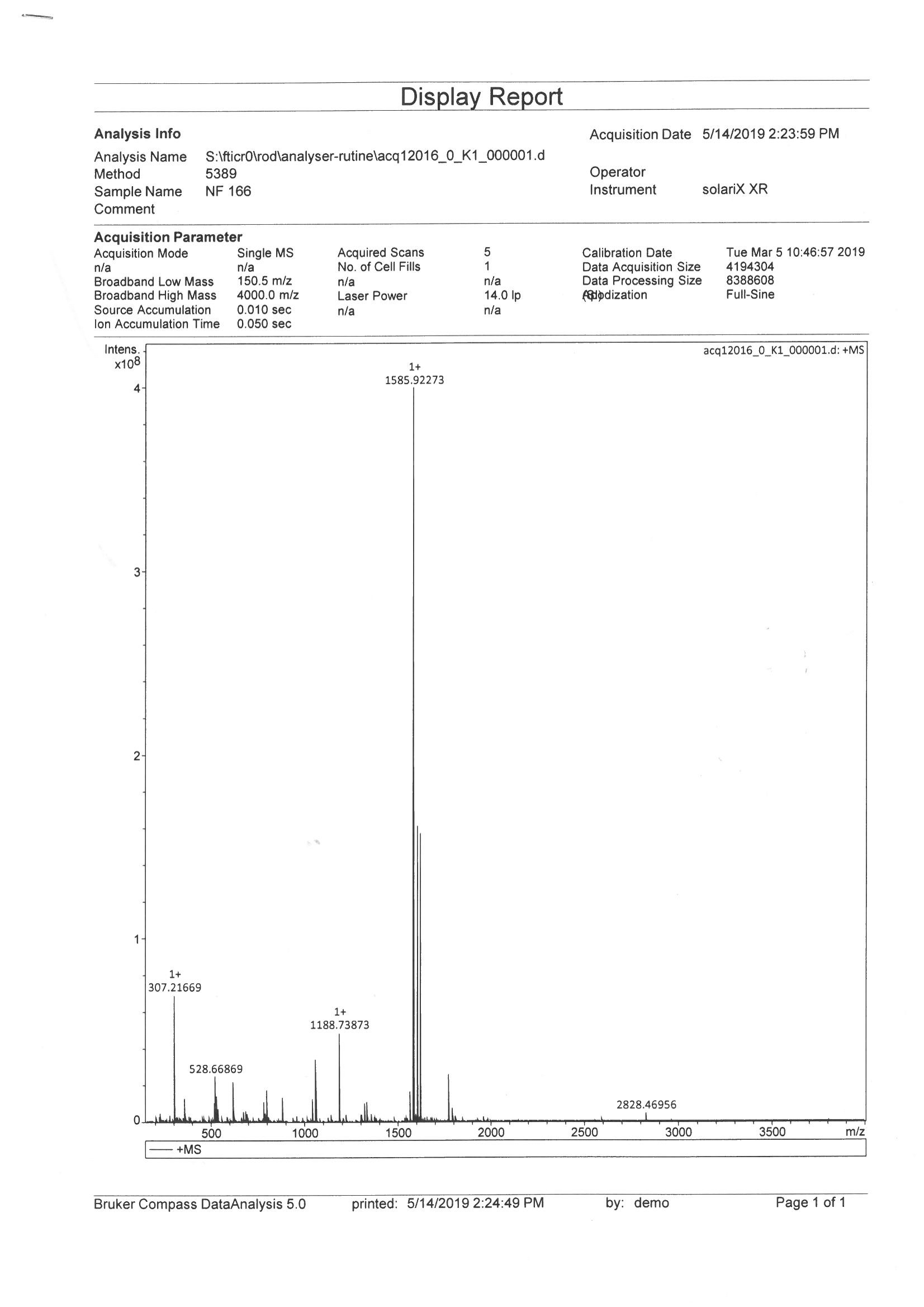 | |
| **HPLC:** Charge: +7. t_R_ = 7.12 min, purity 98.28%. Gradient: 0-60% B during 10 min.  B = 95% MeCN + 0.1% TFA.  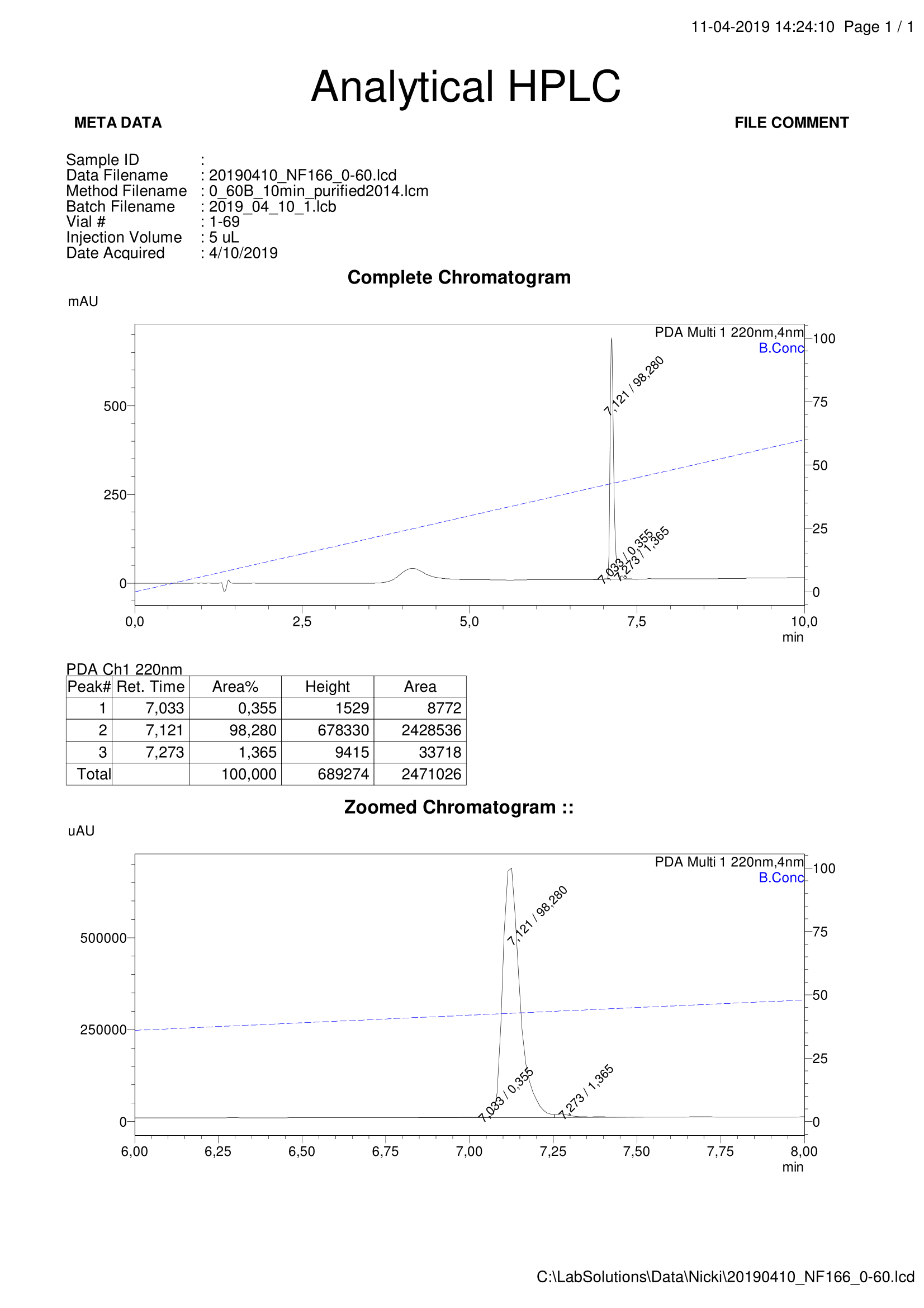 | **HepG2 cell viability**   |

| **Peptidomimetic 6** | |
| --- | --- |
| **HRMS:** calculated for [M+1H]^1+^ 1670.01744, found 1670.02808; ∆M = 6.4 ppm.  **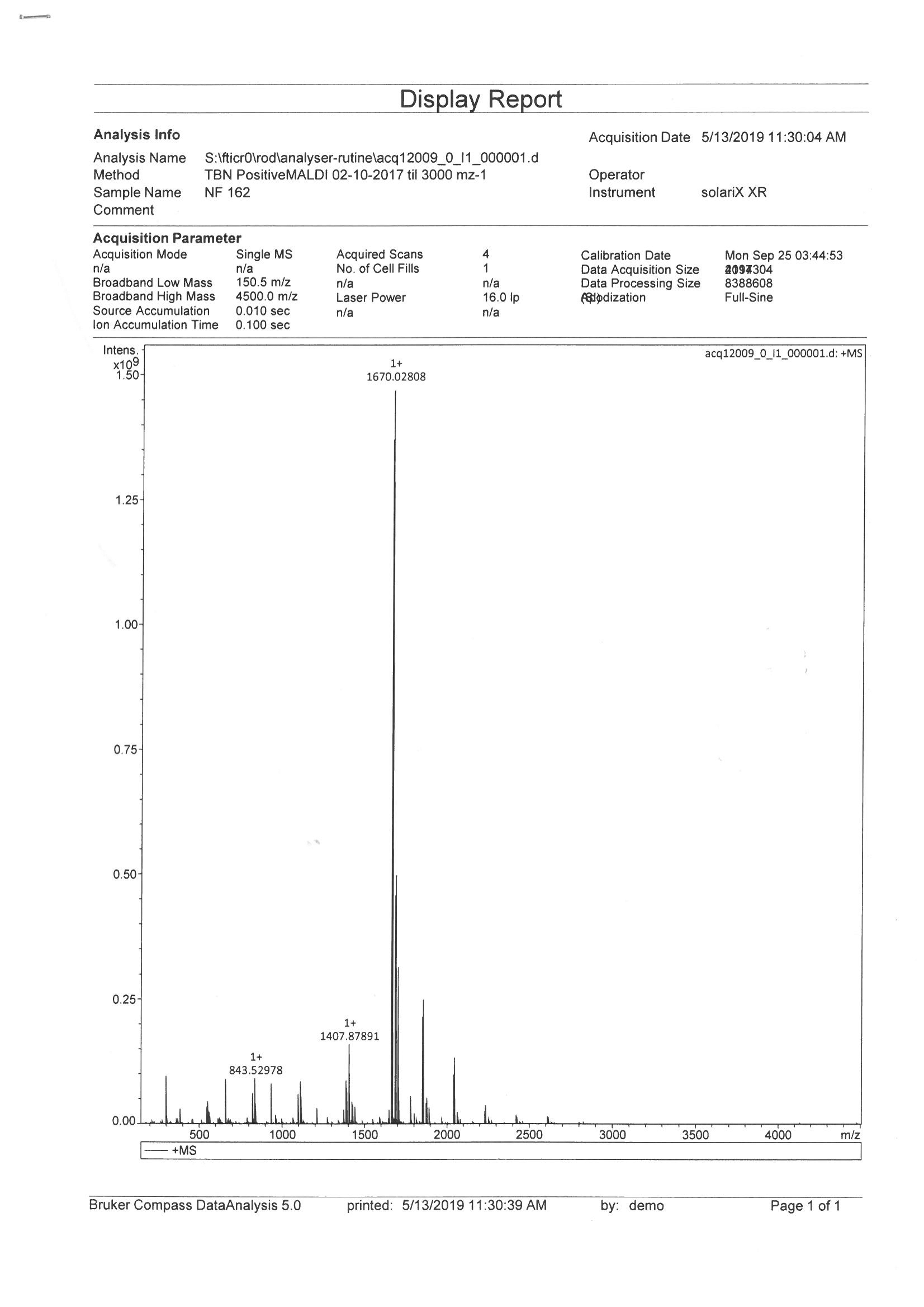** | |
| **HPLC:** Charge: +7. t_R_ = 6.70 min, purity 98.86%. Gradient: 0-60% B during 10 min.  B = 95% MeCN + 0.1% TFA.  **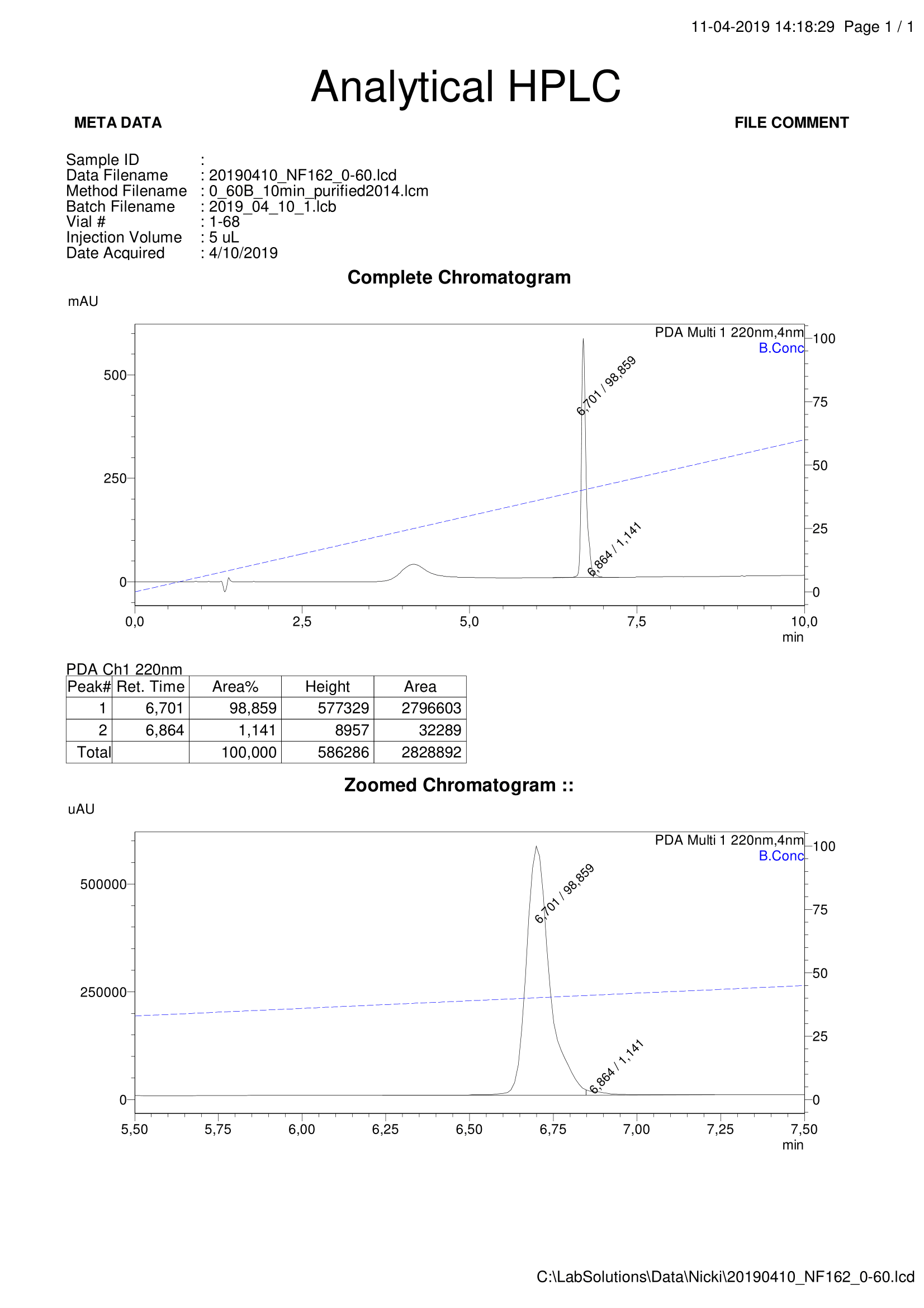** | **HepG2 cell viability**   |

| **Peptidomimetic 7** | |
| --- | --- |
| **HRMS:** calculated for [M+1H]^1+^ 1754.11134, found 1754.10823; ∆M = 1.8 ppm.  **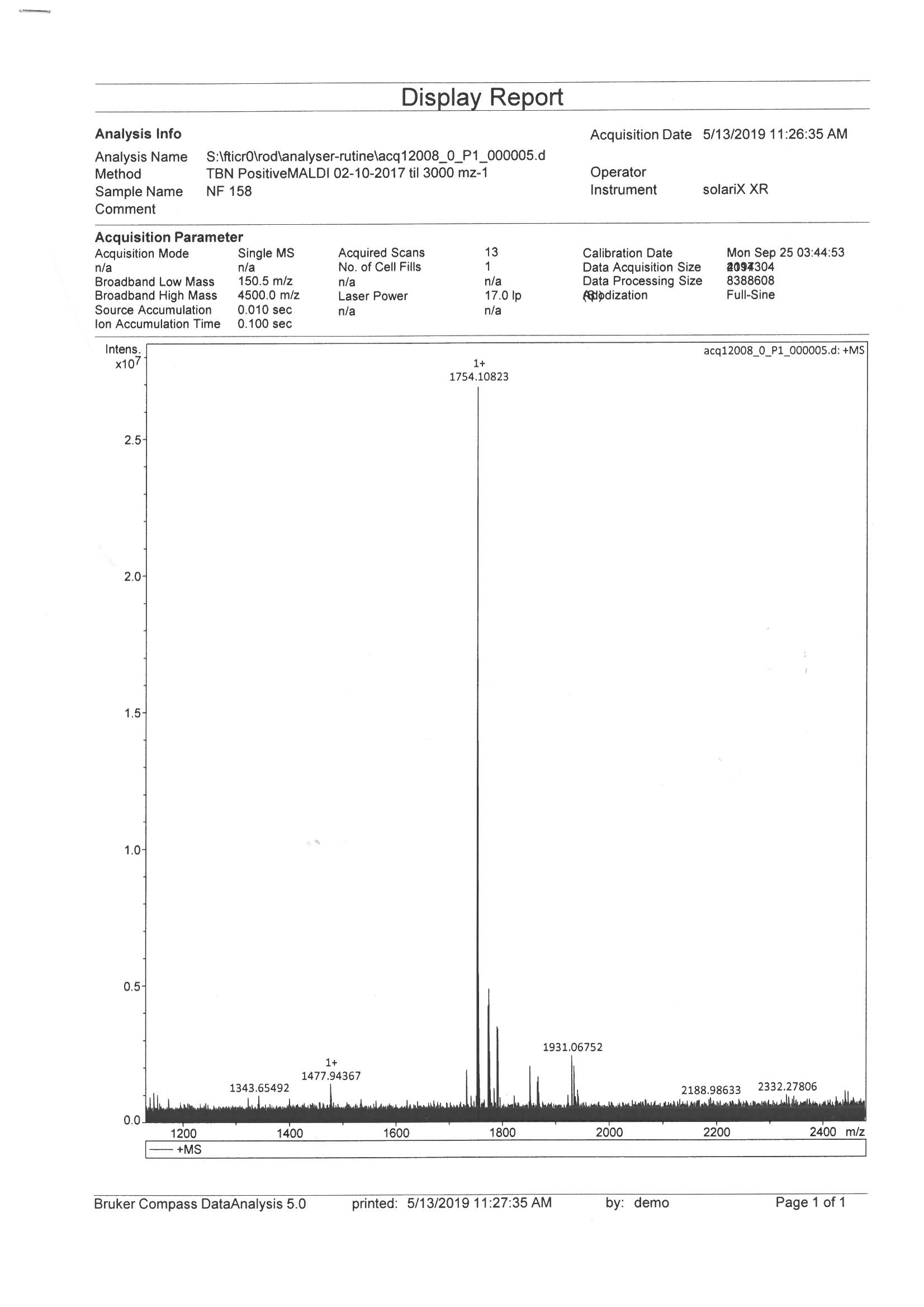** | |
| **HPLC:** Charge: +7. t_R_ = 6.60 min, purity 98.06%. Gradient: 0-60% B during 10 min.  B = 95% MeCN + 0.1% TFA.  **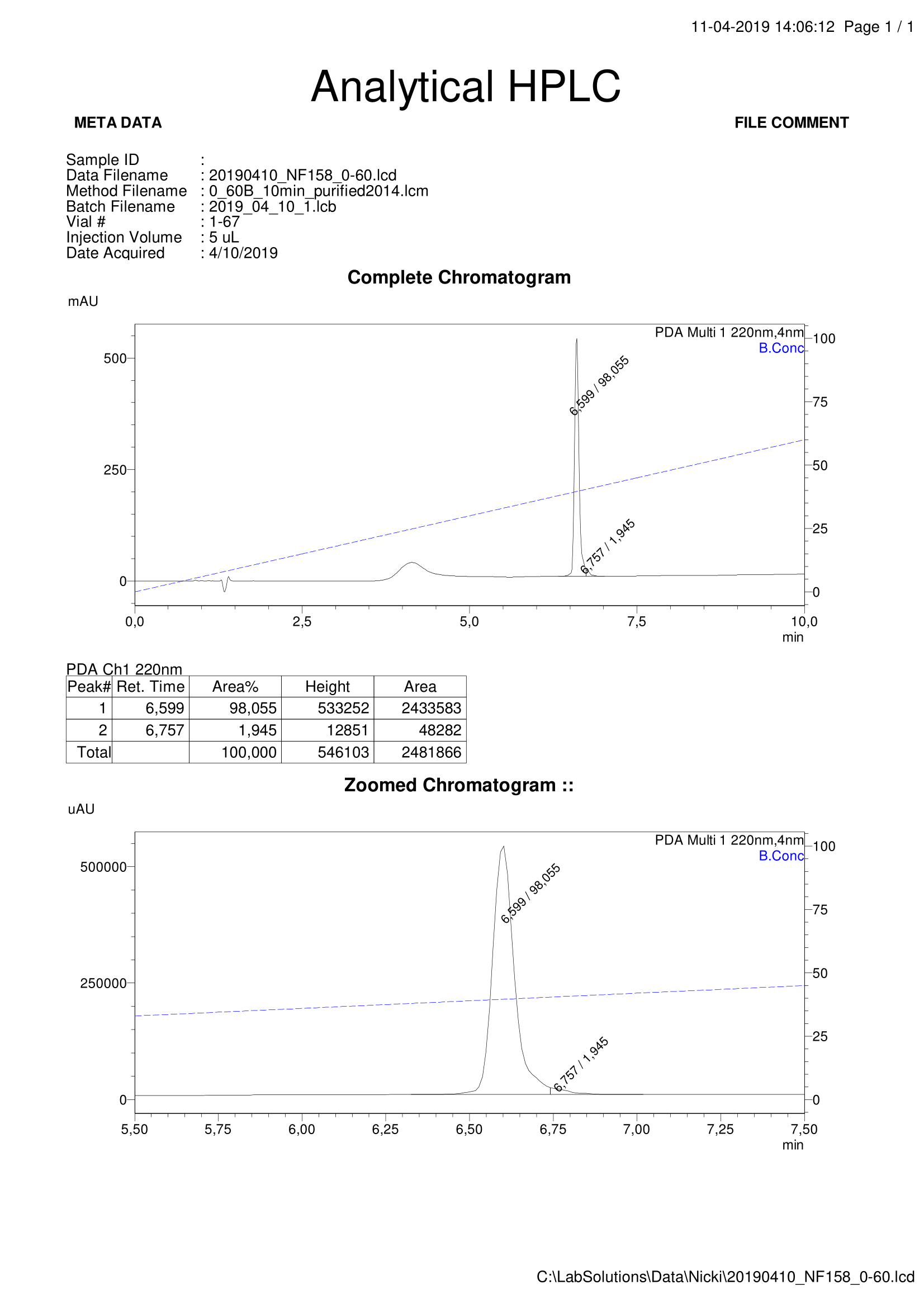** | **HepG2 cell viability**   |

| **Peptidomimetic 8** | |
| --- | --- |
| **HRMS:** calculated for [M+Na]^1+^ 1860.18719, found 1860.18510; ∆M = 1.1 ppm.  **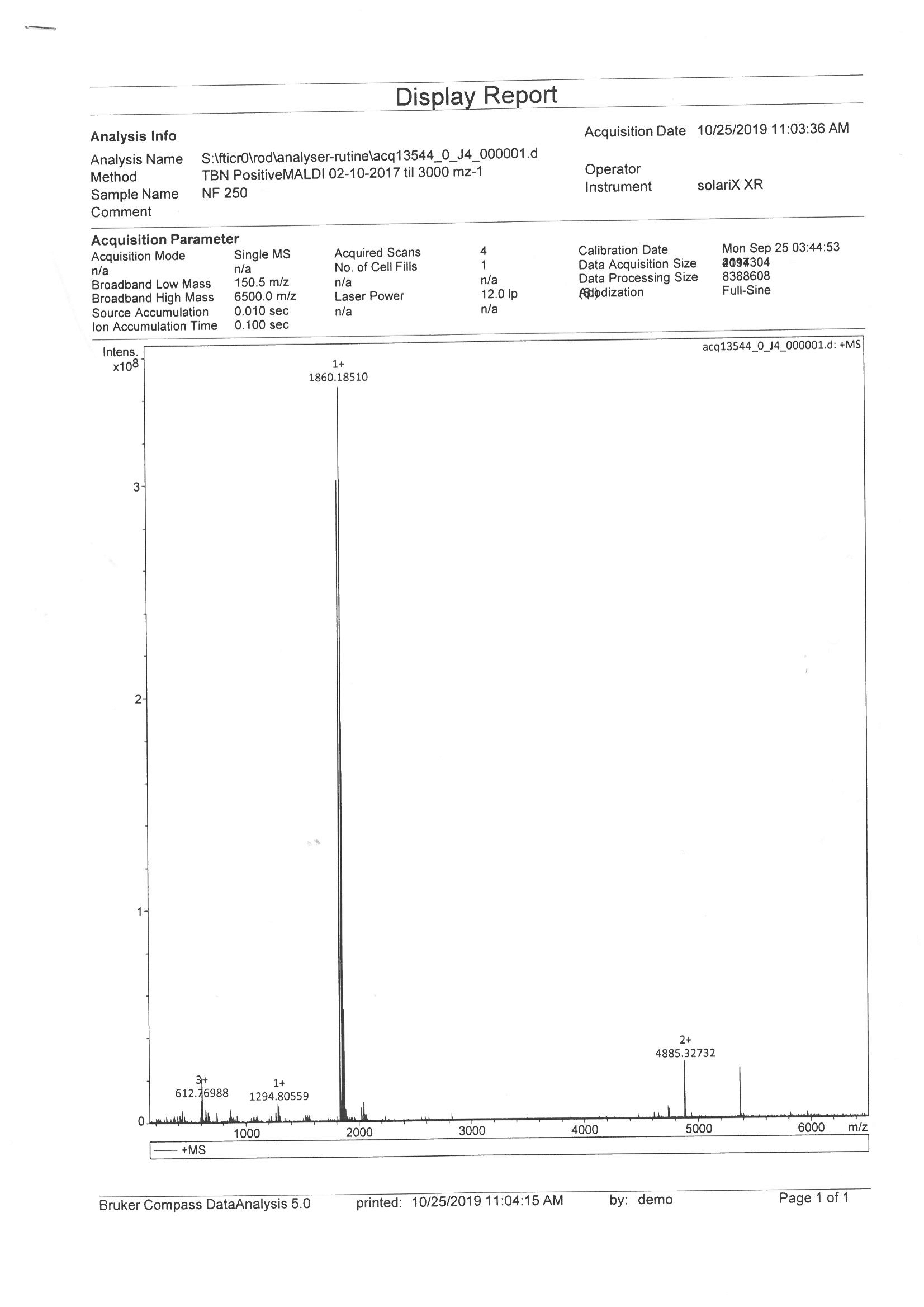** | |
| **HPLC:** Charge: +7. t_R_ = 6.65 min, purity 97.39%. Gradient: 0-60% B during 10 min.  B = 95% MeCN + 0.1% TFA.  **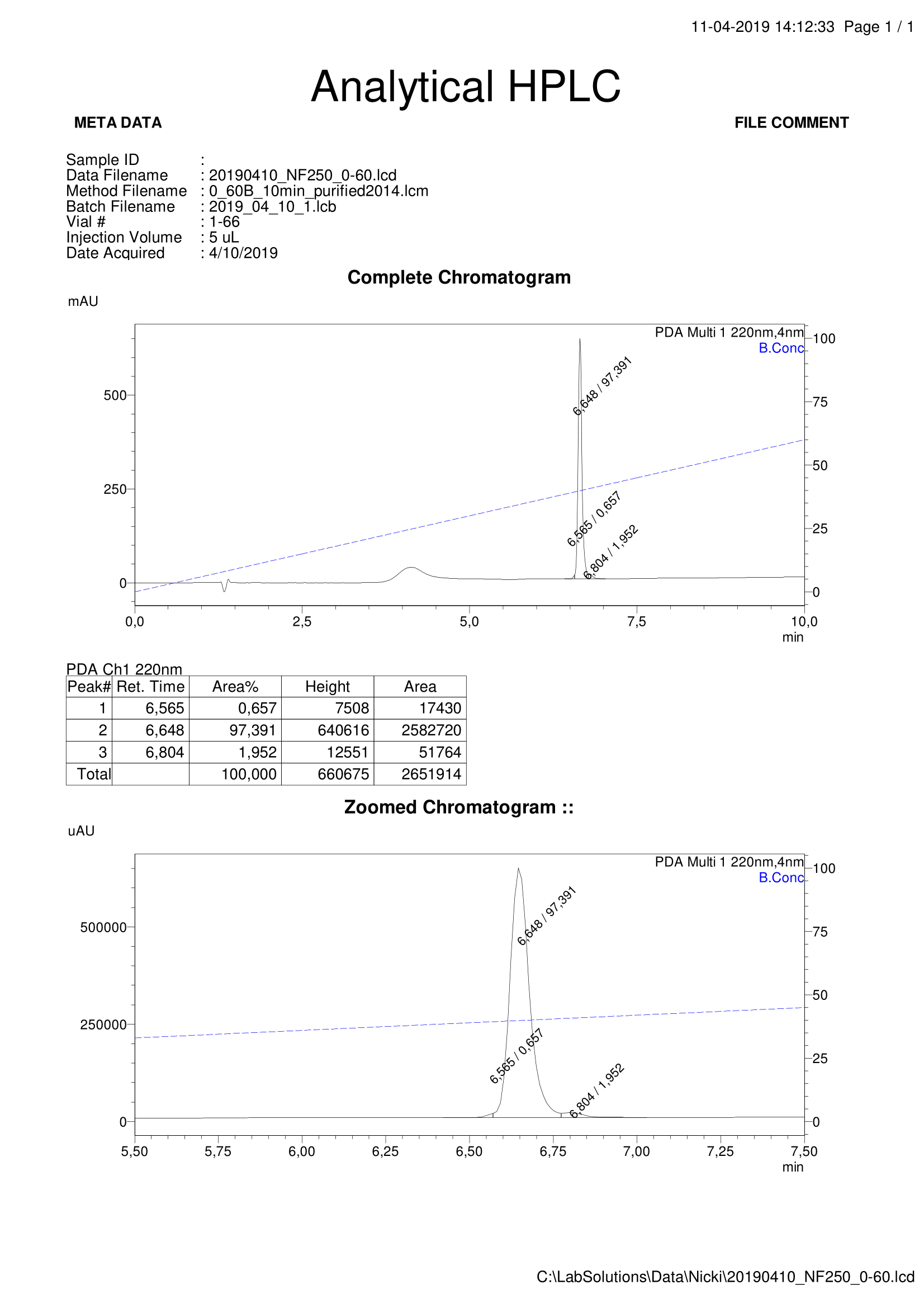** | **HepG2 cell viability**   |

| **Peptidomimetic 9** | | | |  |
| --- | --- | --- | --- | --- |
| **HRMS:** calculated for [M+1H]^1+^ 1500.82629, found 1500.82804; ∆M = 1.2 ppm.  **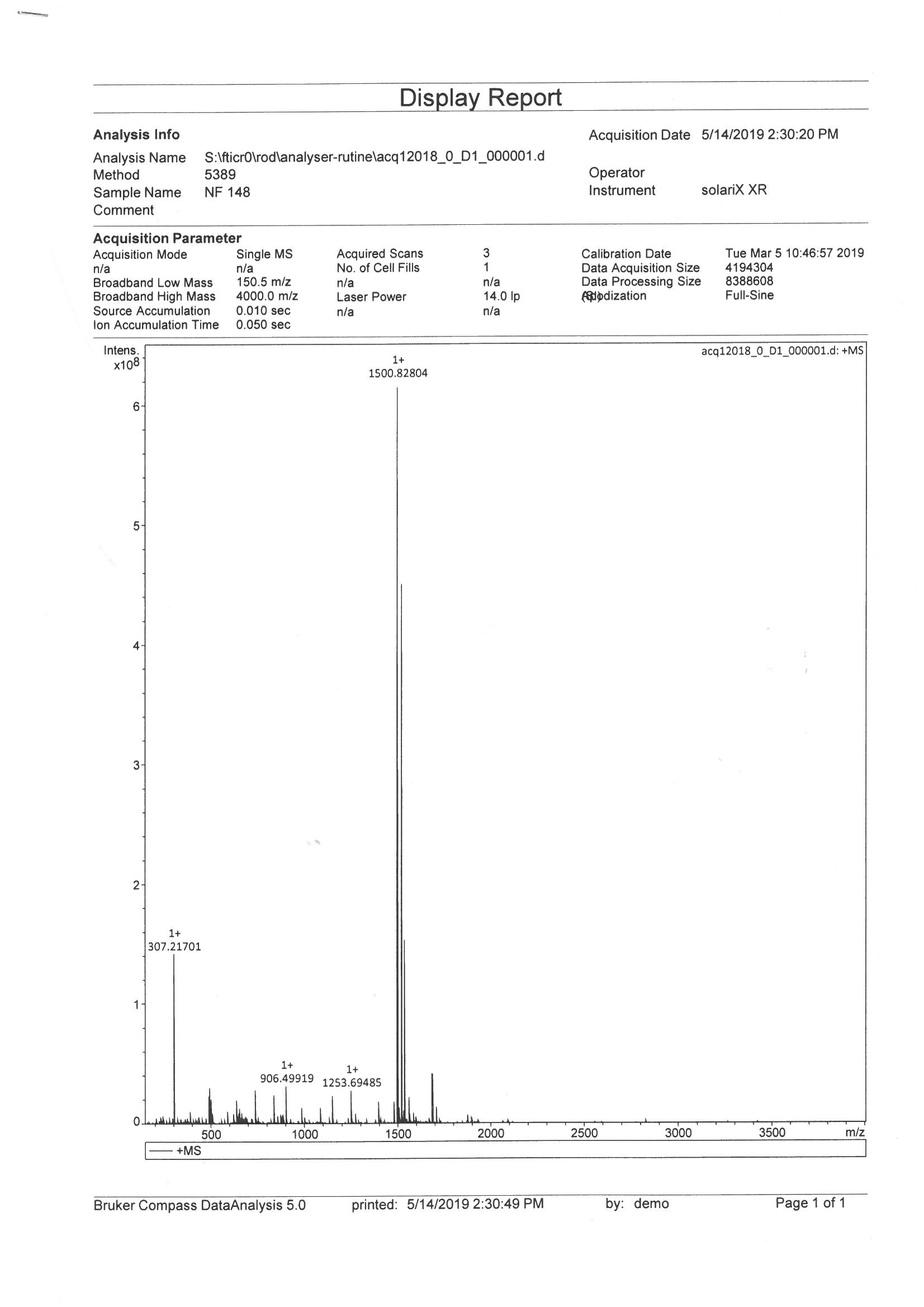** | | | |  |
| **HPLC:** Charge: +7. t_R_ = 6.11 min, purity 95.2%. Gradient: 0-60% B during 10 min.  B = 95% MeCN + 0.1% TFA.  **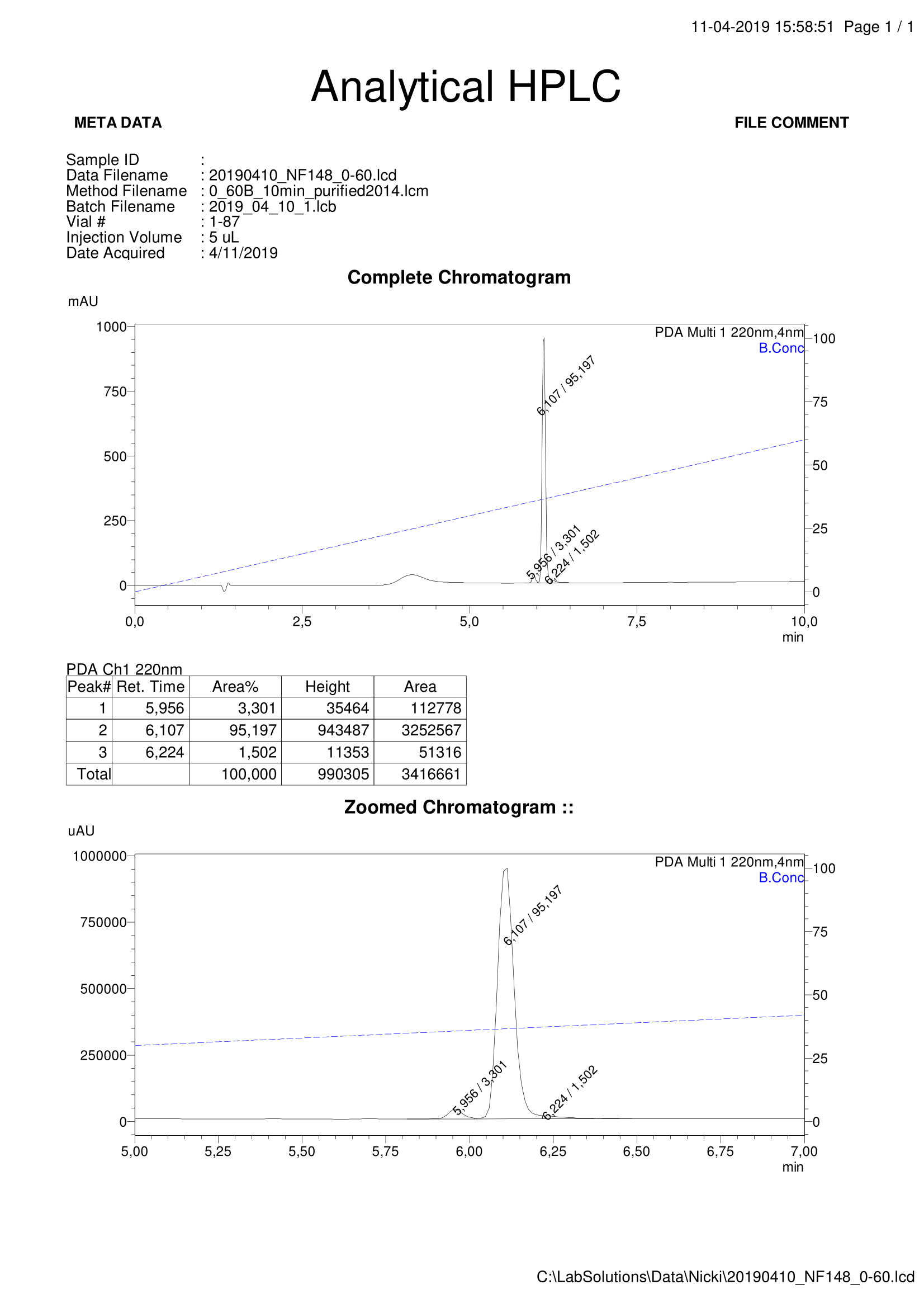** | **HepG2 cell viability**   | | |  |
| **Peptidomimetic 10** | | | |  |
| **HRMS:** calculated for [M+1H]^1+^ 1584.92019, found 1584.91531; ∆M = 3.1 ppm.  **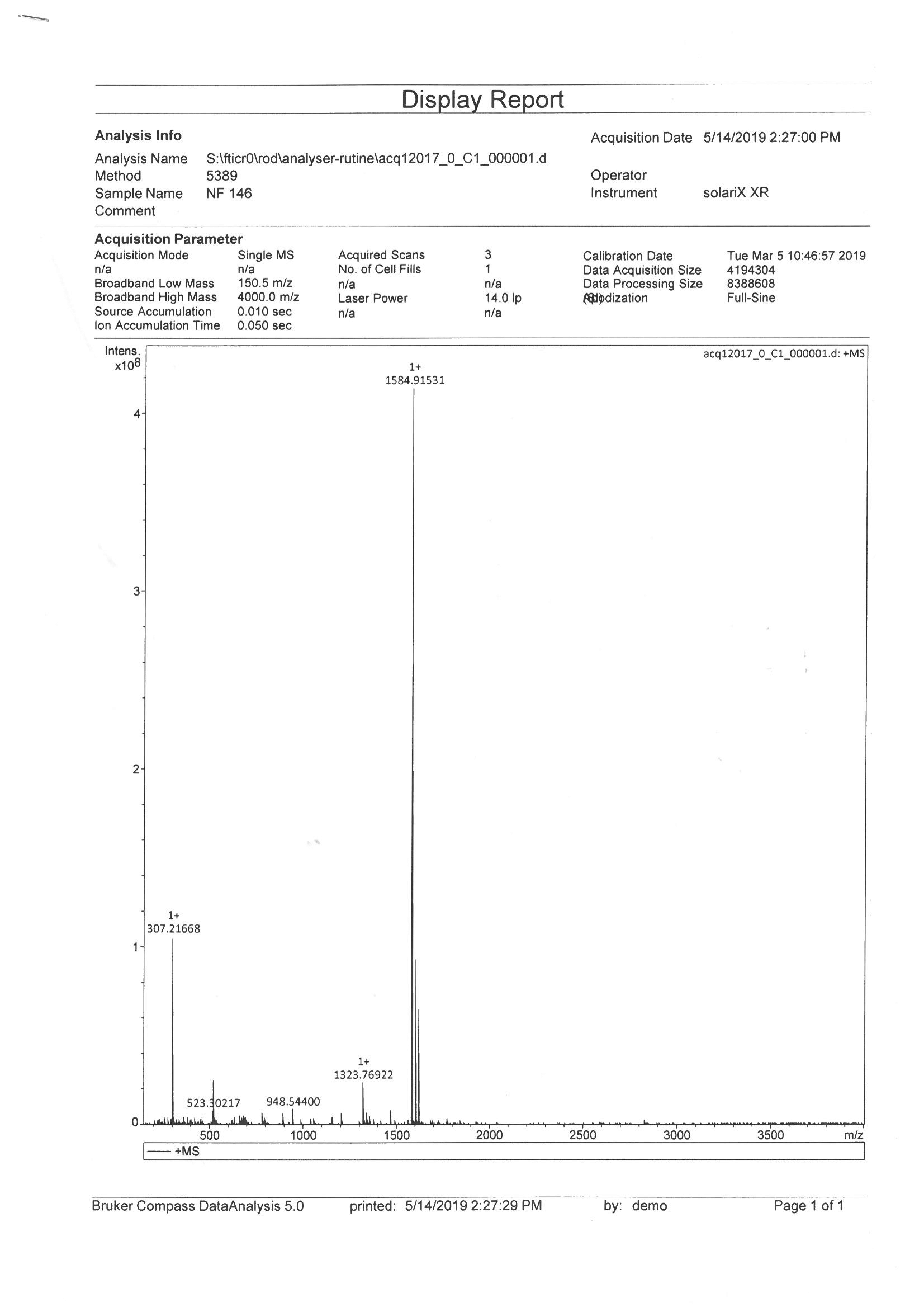** | | | |  |
| **HPLC:** Charge: +7. t_R_ = 5.96 min, purity 86.54%. Gradient: 0-60% B during 10 min.  B = 95% MeCN + 0.1% TFA.  **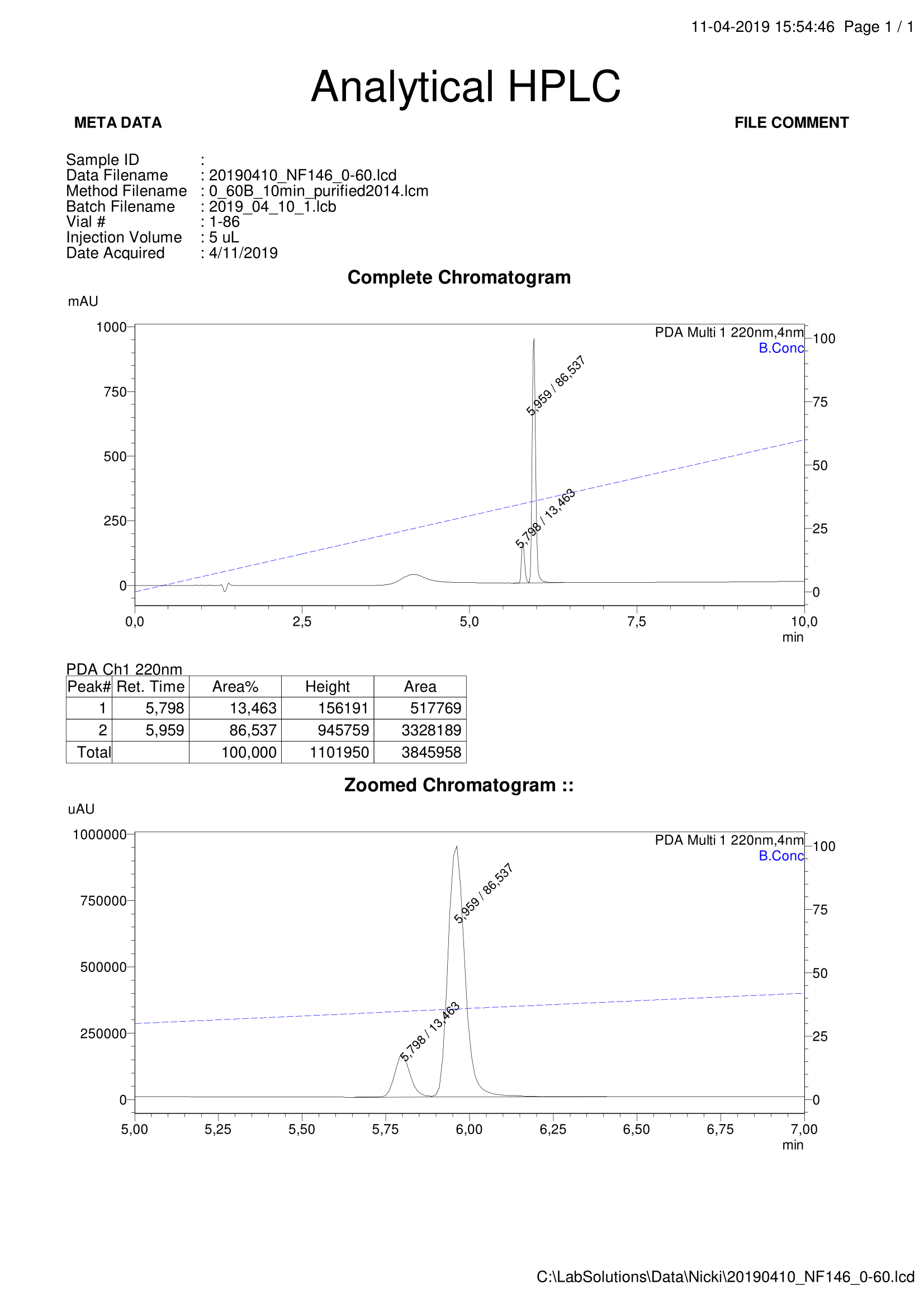** | **HepG2 cell viability**   | | |  |
| **Peptidomimetic 11** | | | |  |
| **HRMS:** calculated for [M+1H]^1+^ 1670.01744, found 1670.0168; ∆M = 0.4 ppm.  **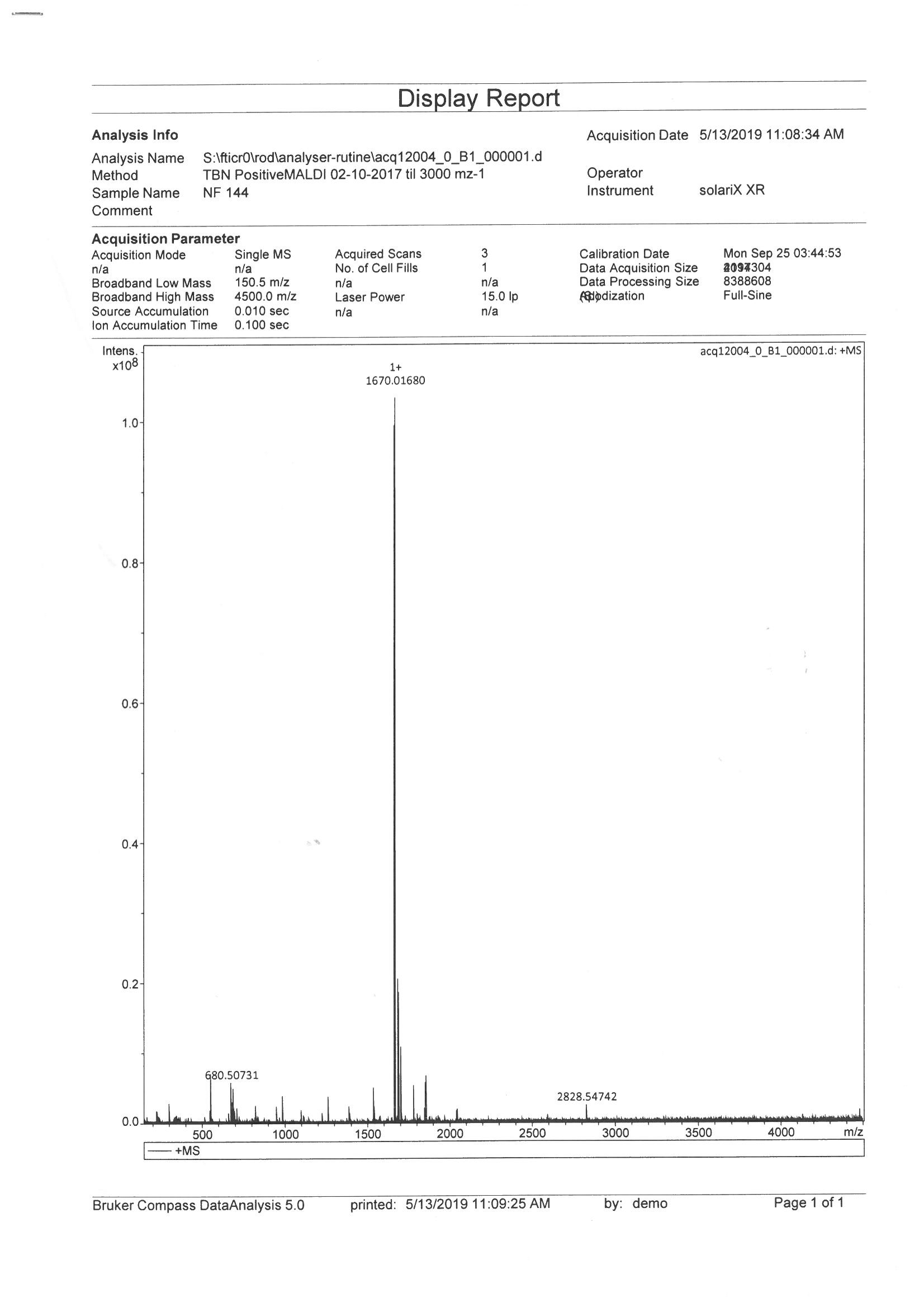** | | | |  |
| **HPLC:** Charge: +7. t_R_ = 5.92 min, purity 97.32%. Gradient: 0-60% B during 10 min.  B = 95% MeCN + 0.1% TFA.  **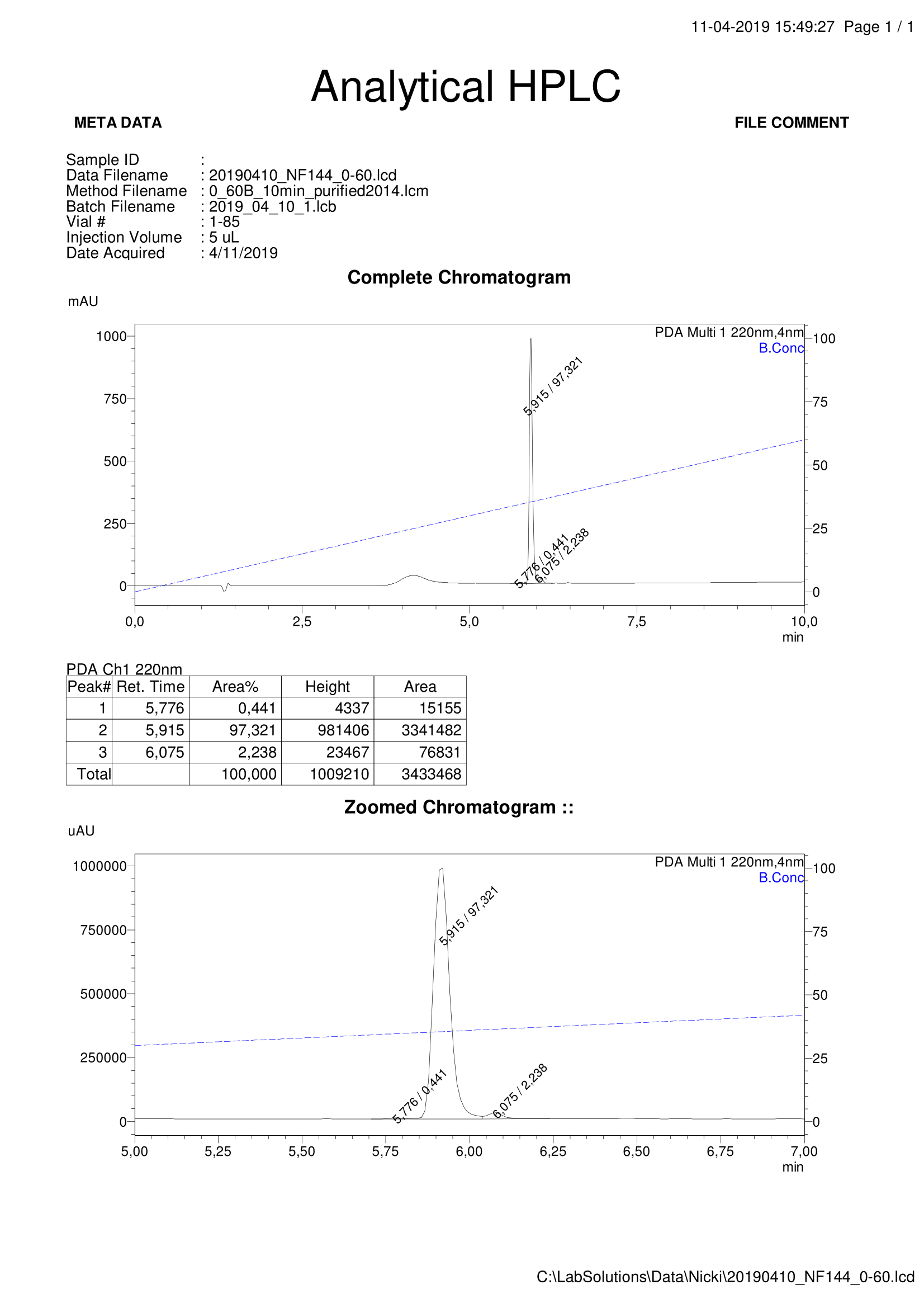** |  | | |  |
| **Peptidomimetic 12** | | | |  |
| **HRMS:** calculated for [M+1H]^1+^ 1754.11134, found 1754.10929; ∆M = 1.2 ppm.  **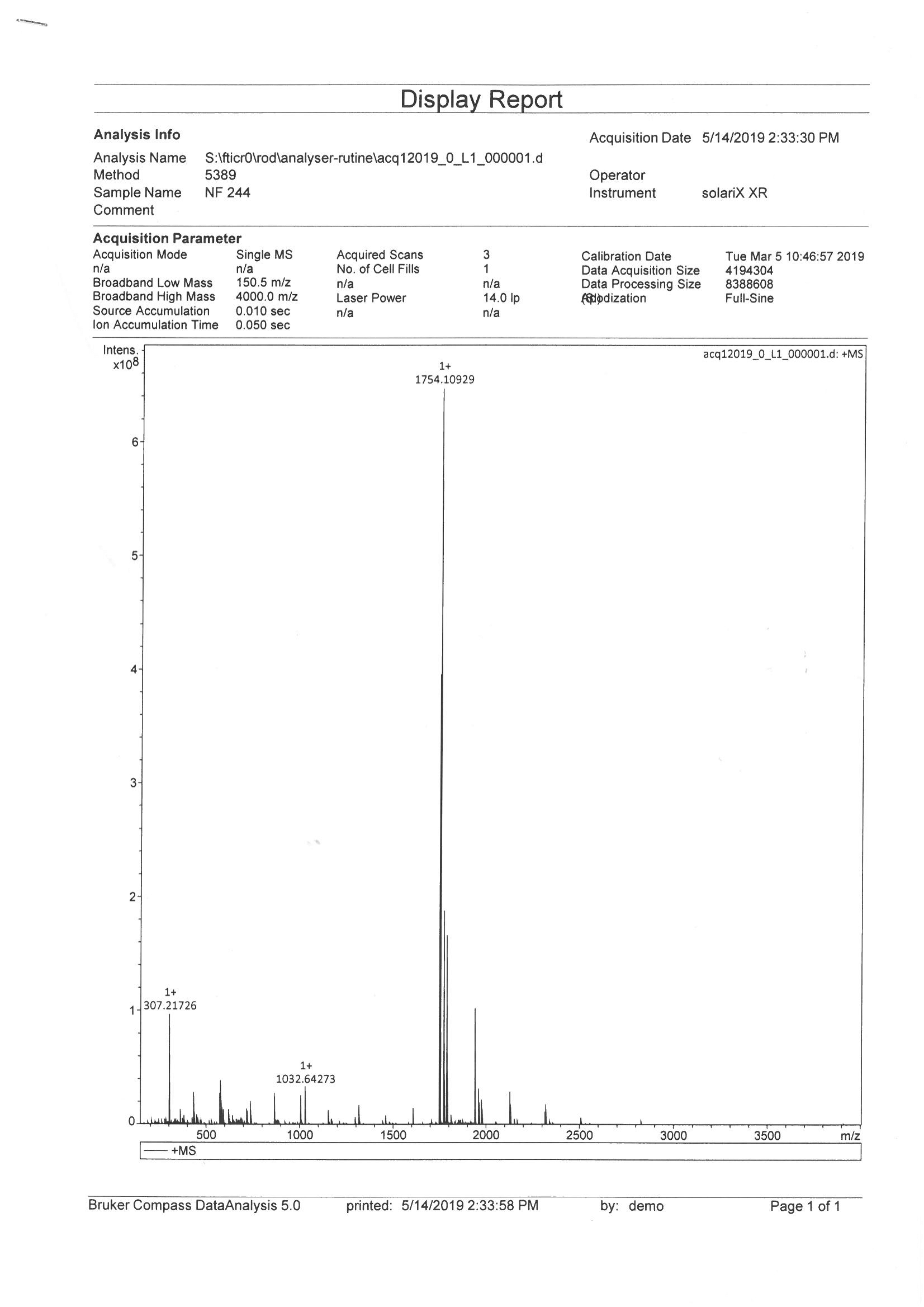** | | | |  |
| **HPLC:** Charge: +7. t_R_ = 6.11 min, purity 98.18%. Gradient: 0-60% B during 10 min.  B = 95% MeCN + 0.1% TFA.  **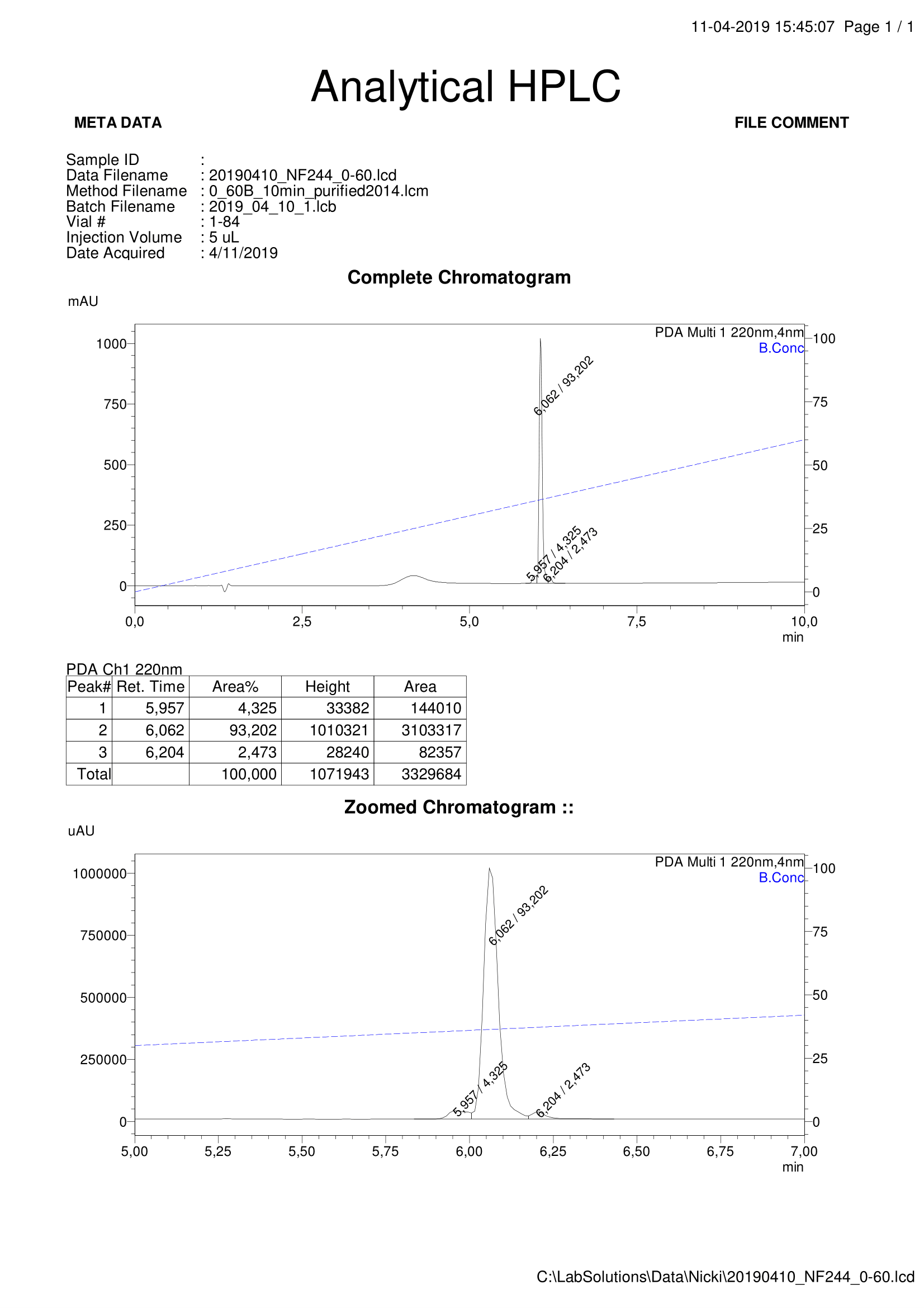** | **HepG2 cell viability**   | | |  |
| **Peptidomimetic 13** | | | |  |
| **HRMS:** calculated for [M+1H]^1+^ 1584.92019, found 1584.92028; ∆M = 0.1 ppm.  **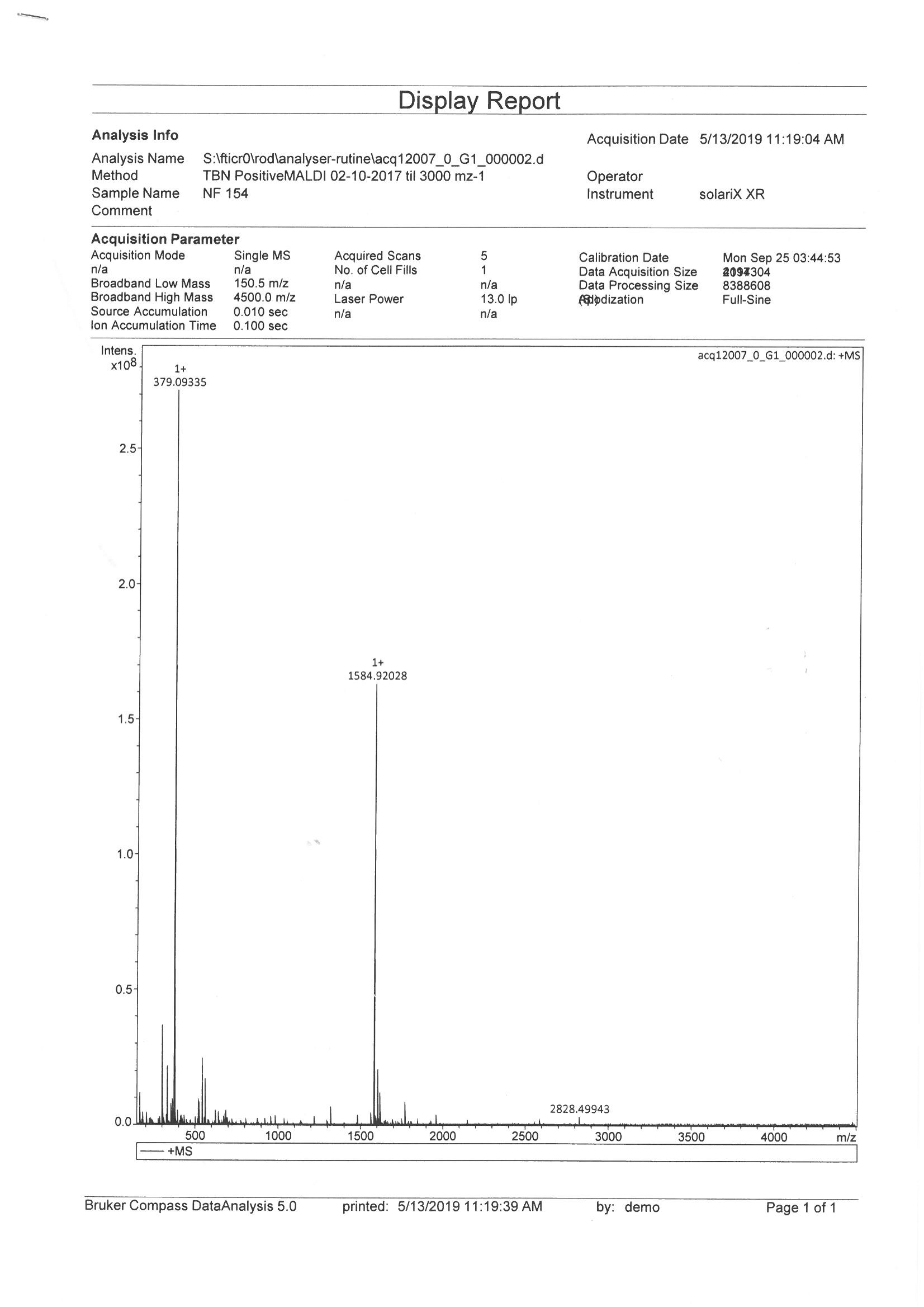** | | | |  |
| **HPLC:** Charge: +7. t_R_ = 6.75 min, purity 98.98%. Gradient: 0-60% B during 10 min.  B = 95% MeCN + 0.1% TFA.  **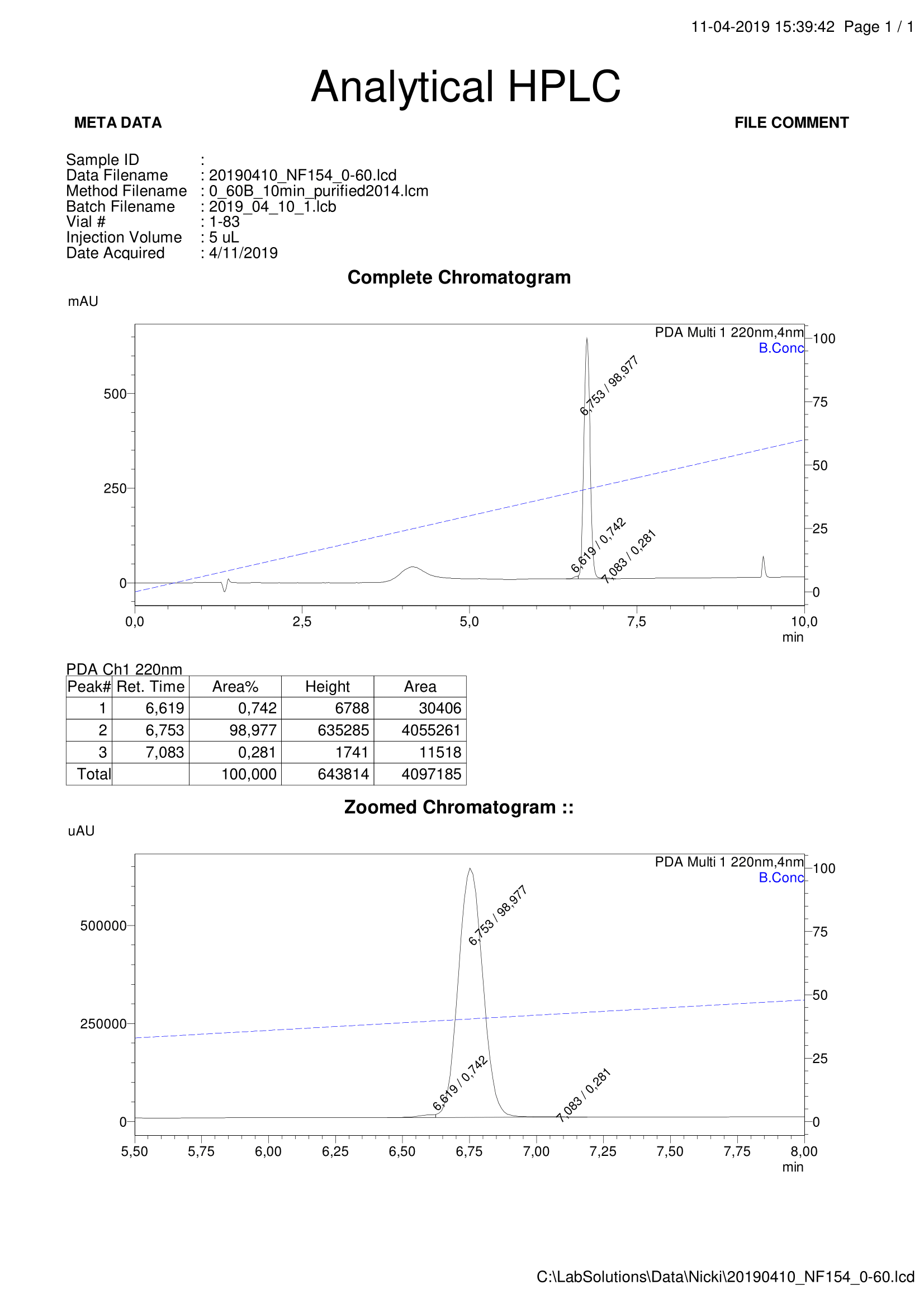** | | | **HepG2 cell viability**   |  |
| **Peptidomimetic 14** | | | |  |
| **HRMS:** calculated for [M+1H]^1+^ 1670.01744, found 1670.01900; ∆M = 0.9 ppm.  **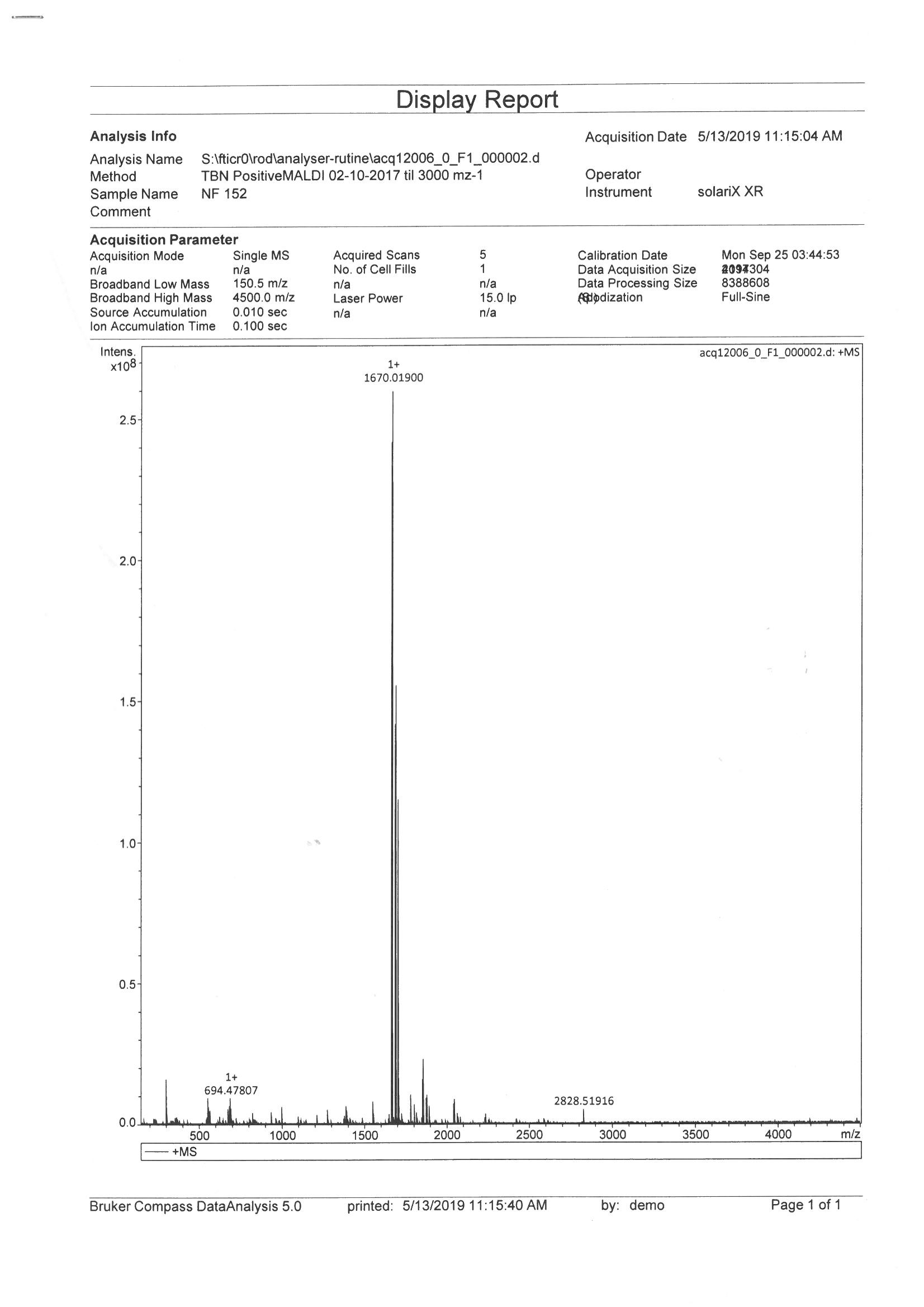** | | | |  |
| **HPLC:** Charge: +7. t_R_ = 6.58 min, purity 99.64%. Gradient: 0-60% B during 10 min.  B = 95% MeCN + 0.1% TFA.  **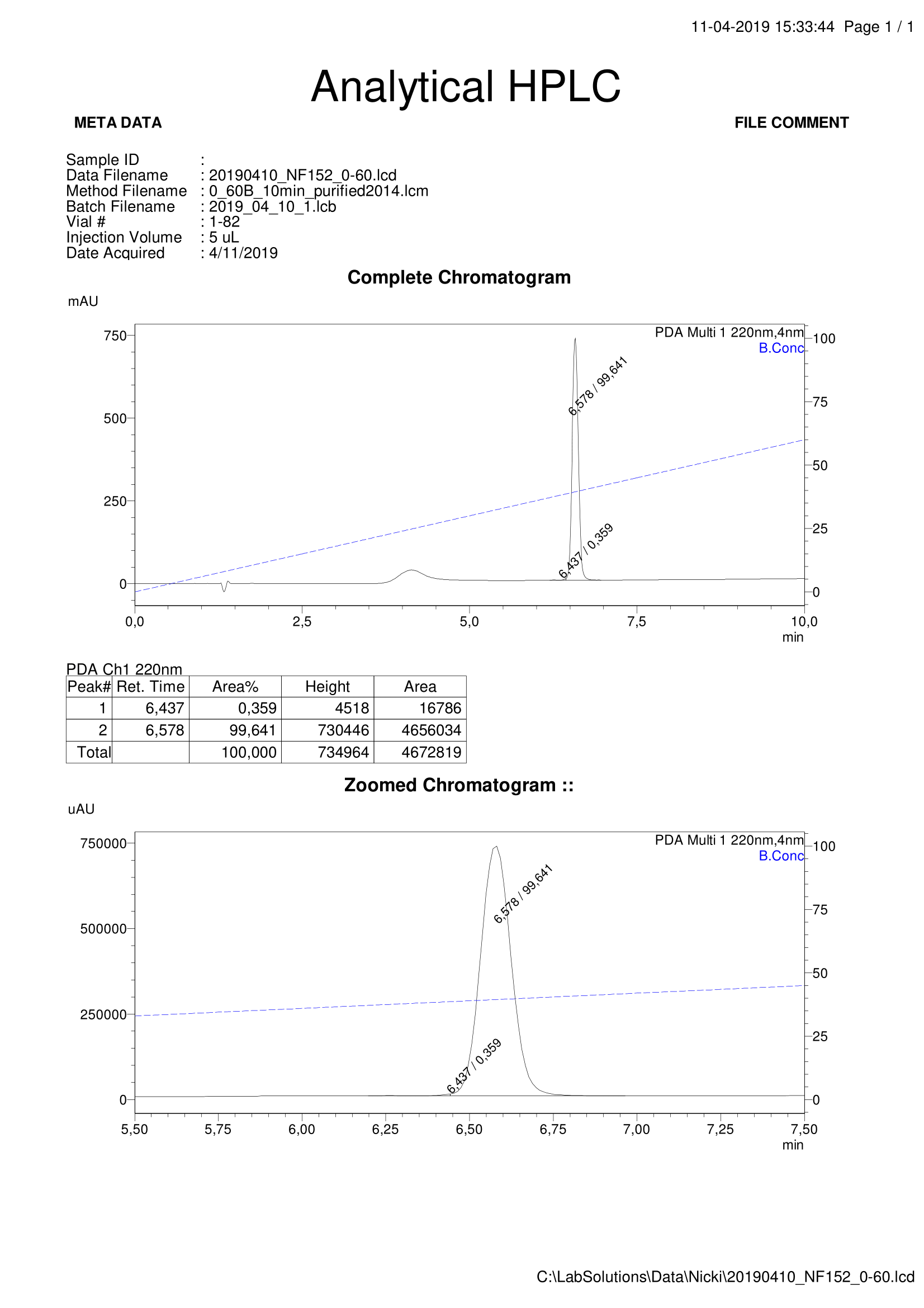** | | | **HepG2 cell viability**   |  |
| **Peptidomimetic 15** | | | |  |
| **HRMS:** calculated for [M+1H]^1+^ 1754.11134, found 1754.10844; ∆M = 1.7 ppm.  **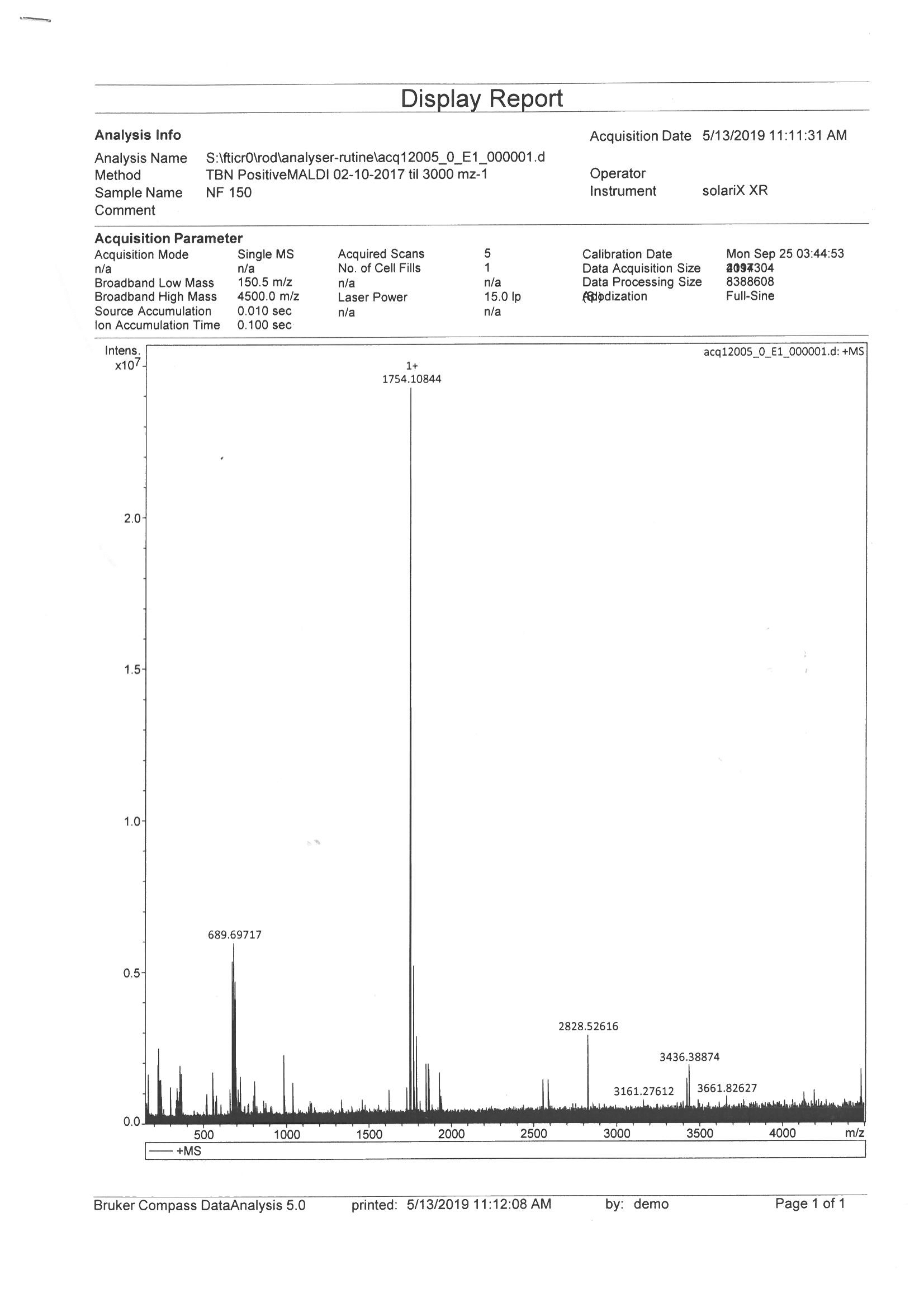** | | | |  |
| **HPLC:** Charge: +7. t_R_ = 6.58 min, purity 99.84%. Gradient: 0-60% B during 10 min.  B = 95% MeCN + 0.1% TFA.  **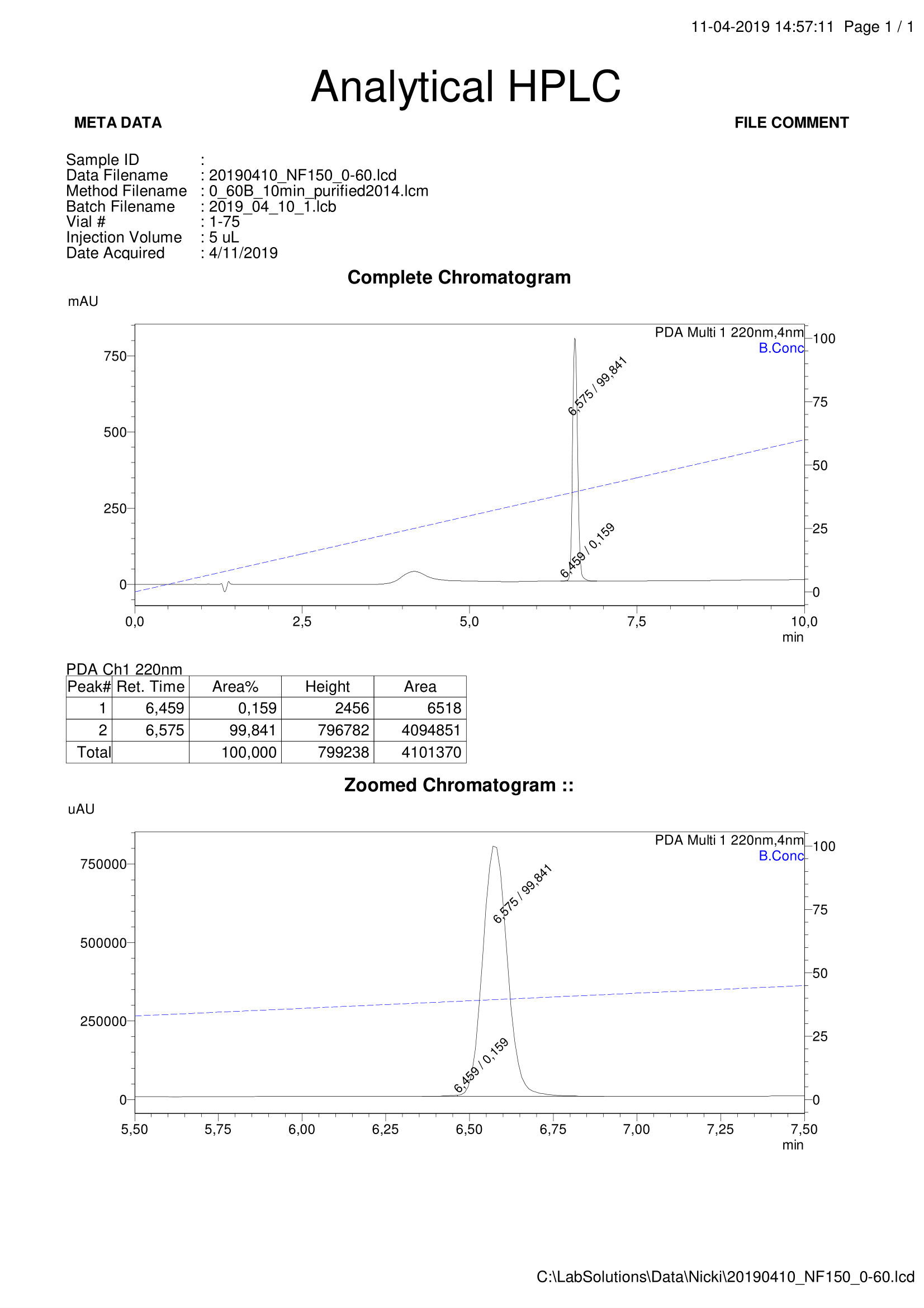** | | | **HepG2 cell viability**   |  |
| **Peptidomimetic 16** | | | | |
| **HRMS:** calculated for [M+1H]^1+^ 1838.20524, found 1838.20179; ∆M = 1.9 ppm.  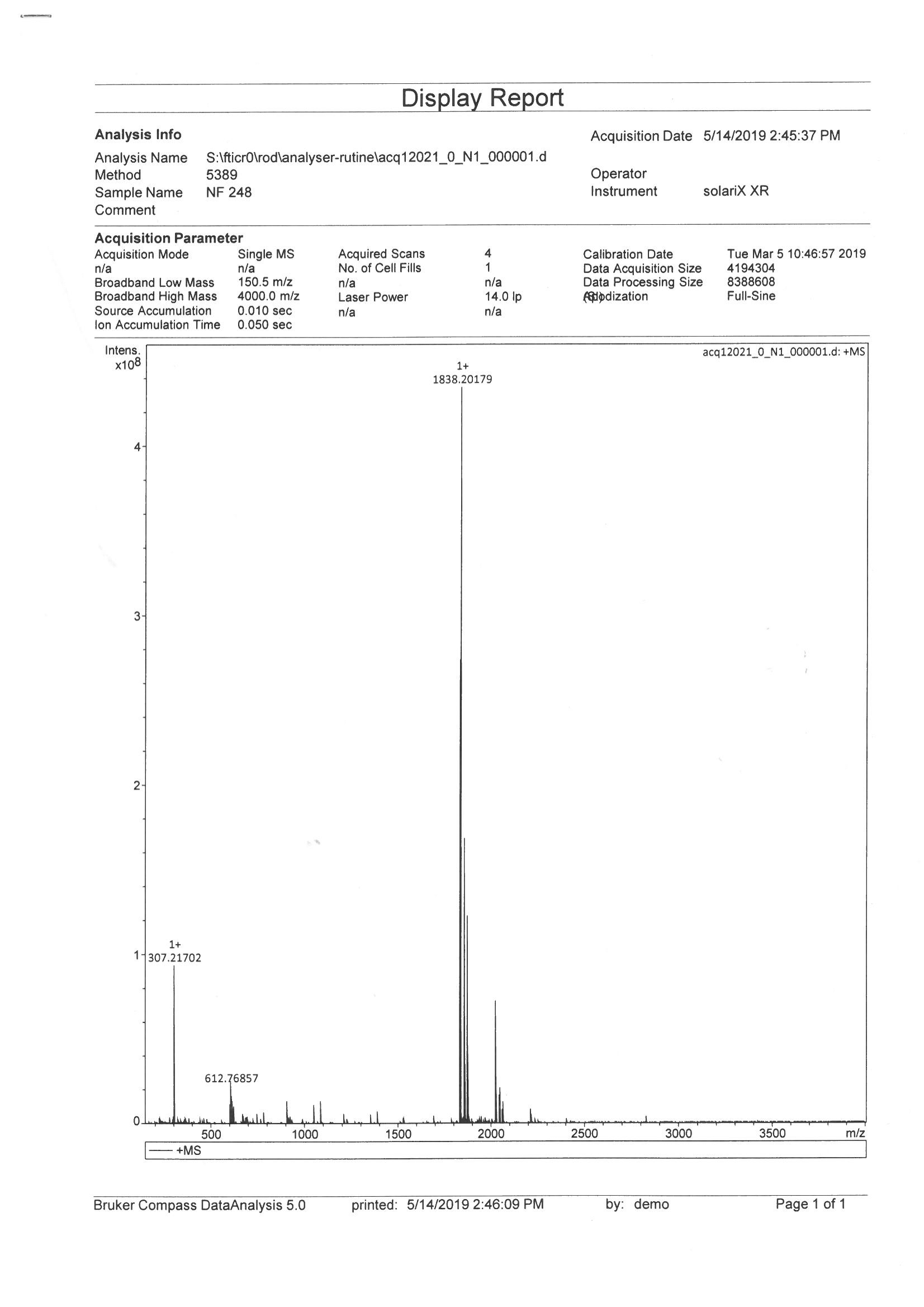 | | | | |
| **HPLC:** Charge: +7. t_R_ = 6.68 min, purity 99.02%. Gradient: 0-60% B during 10 min.  B = 95% MeCN + 0.1% TFA.  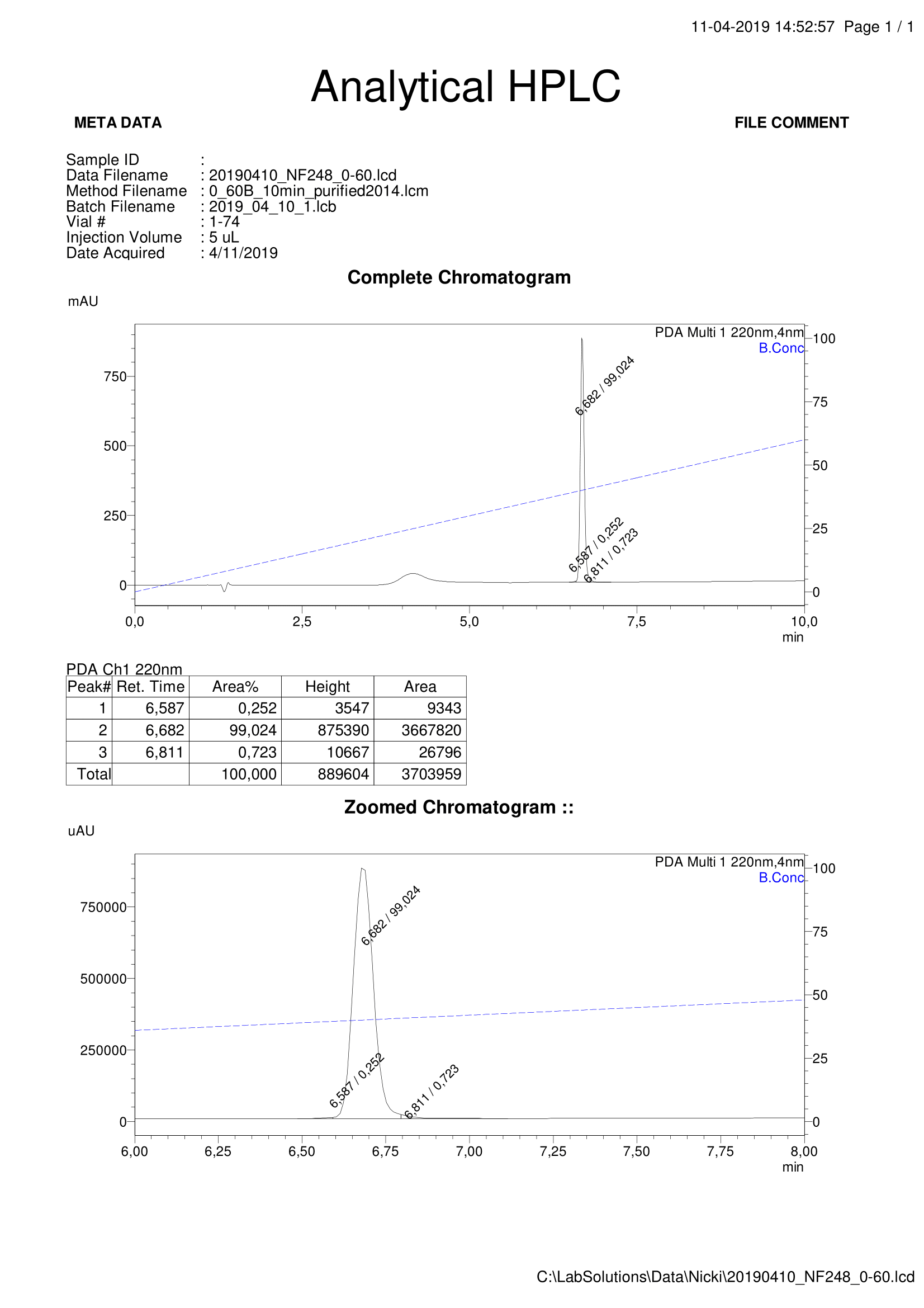 | | **HepG2 cell viability**   | | |

# Characterisation of peptoid building blocks

**Peptoid building block 17:** Fmoc-NDab(Boc)-OH

**HRMS:** calculated for [M+Na]^1+^ 463.18396, found 463.18550; ∆M = 3.3 ppm.


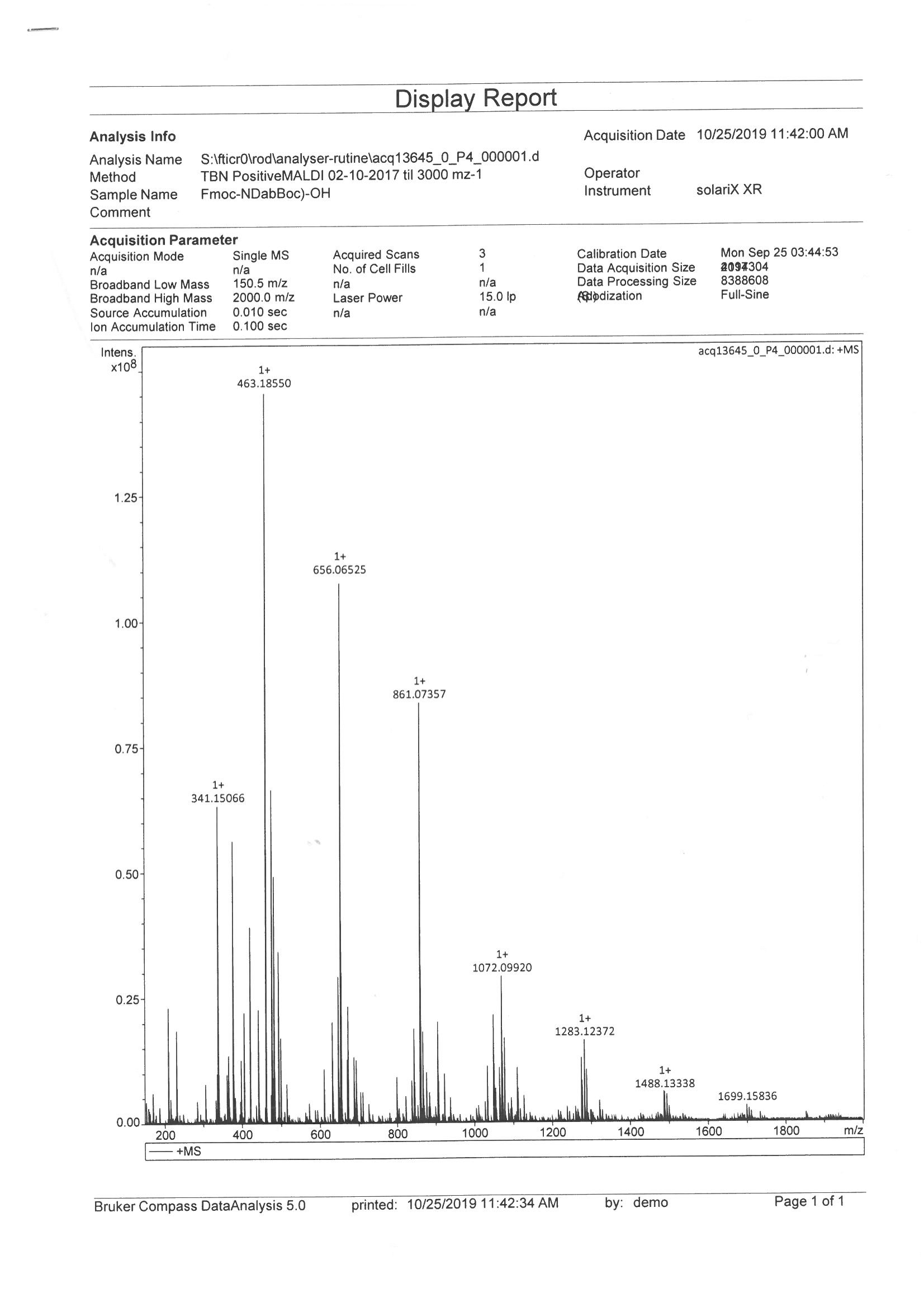


**HPLC:** t_R_ = 6.53 min, purity 100.00%. Gradient: 30-100% B during 10 min. B = 95% MeCN + 0.1% TFA.


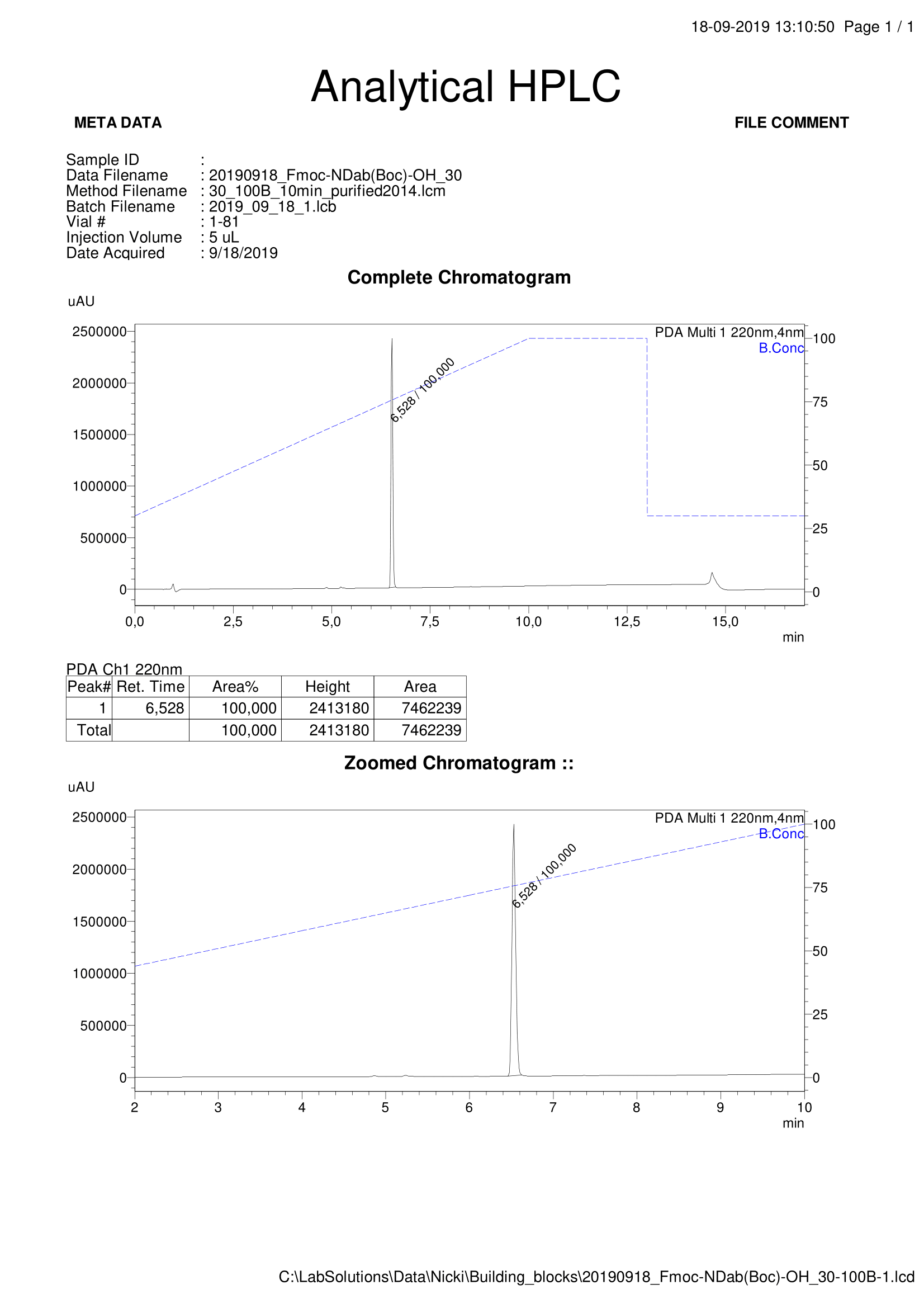


**^13^C-NMR**


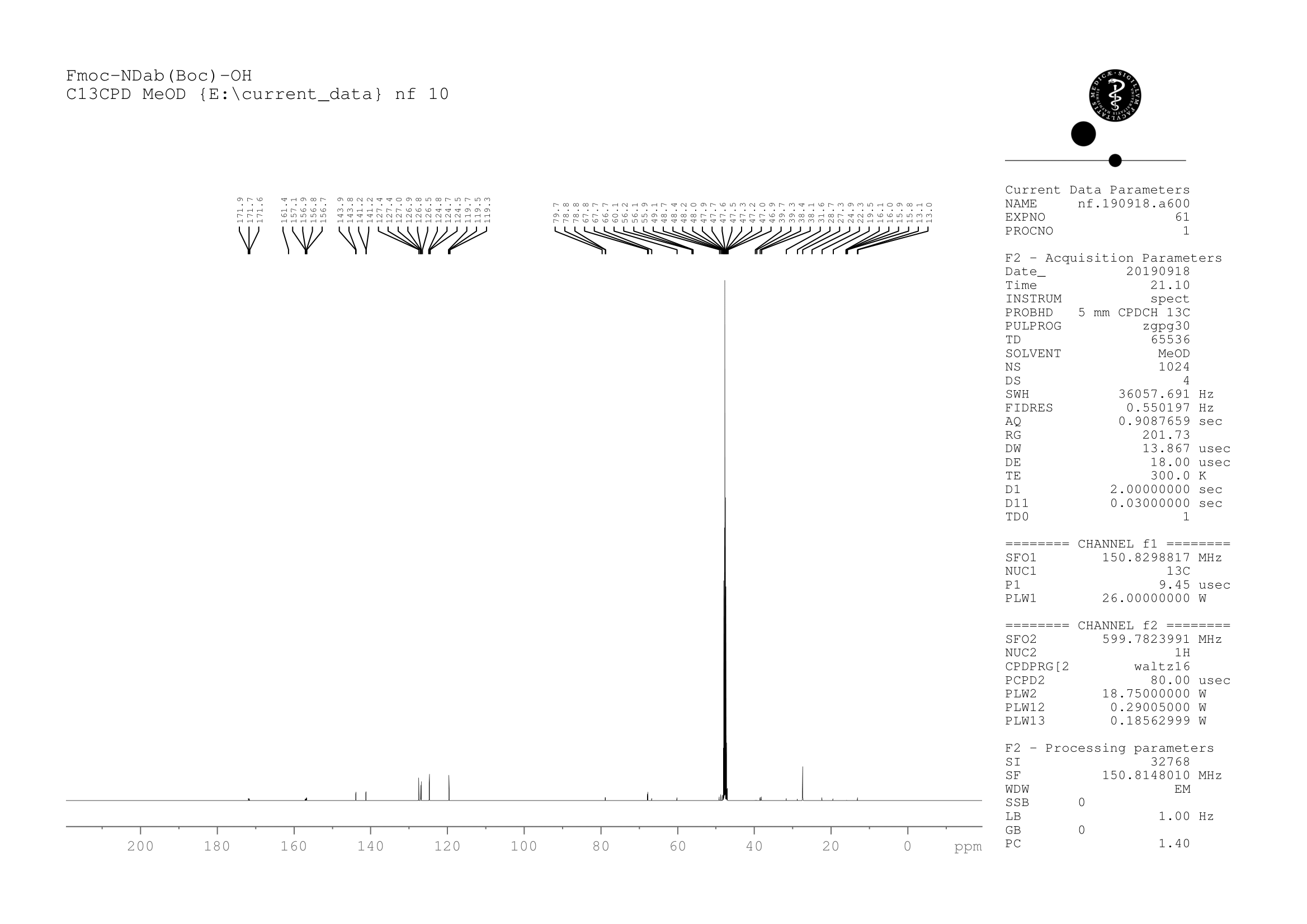


**Peptoid building block 18:** Fmoc-NOrn(Boc)-OH

**HRMS:** calculated for [M+Na]^1+^ 477.19961, found 477.20298; ∆M = 7.1 ppm.


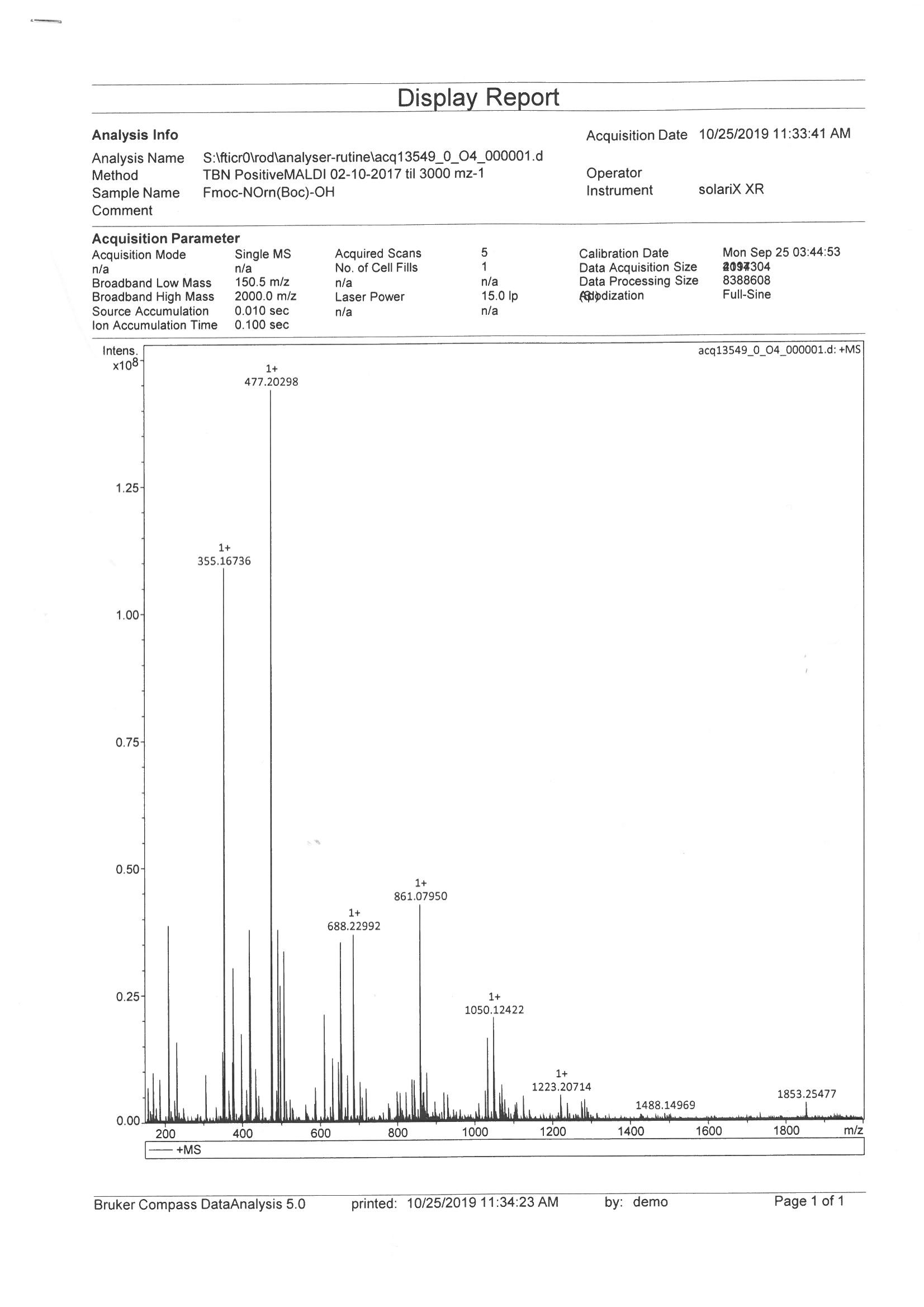


**HPLC:** t_R_ = 6.72 min, purity 100.00%. Gradient: 30-100% B during 10 min. B = 95% MeCN + 0.1% TFA.


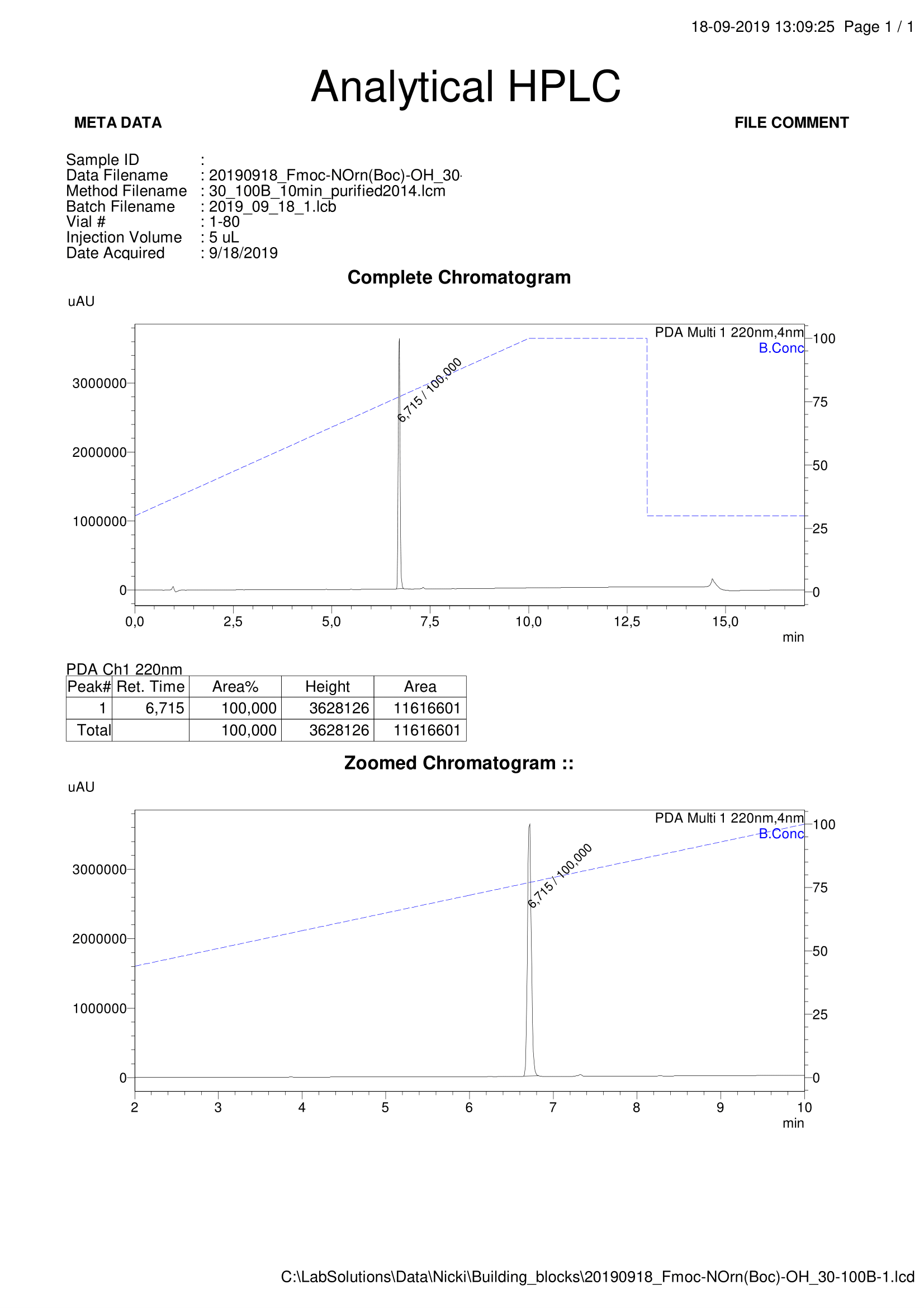


**^13^C-NMR**


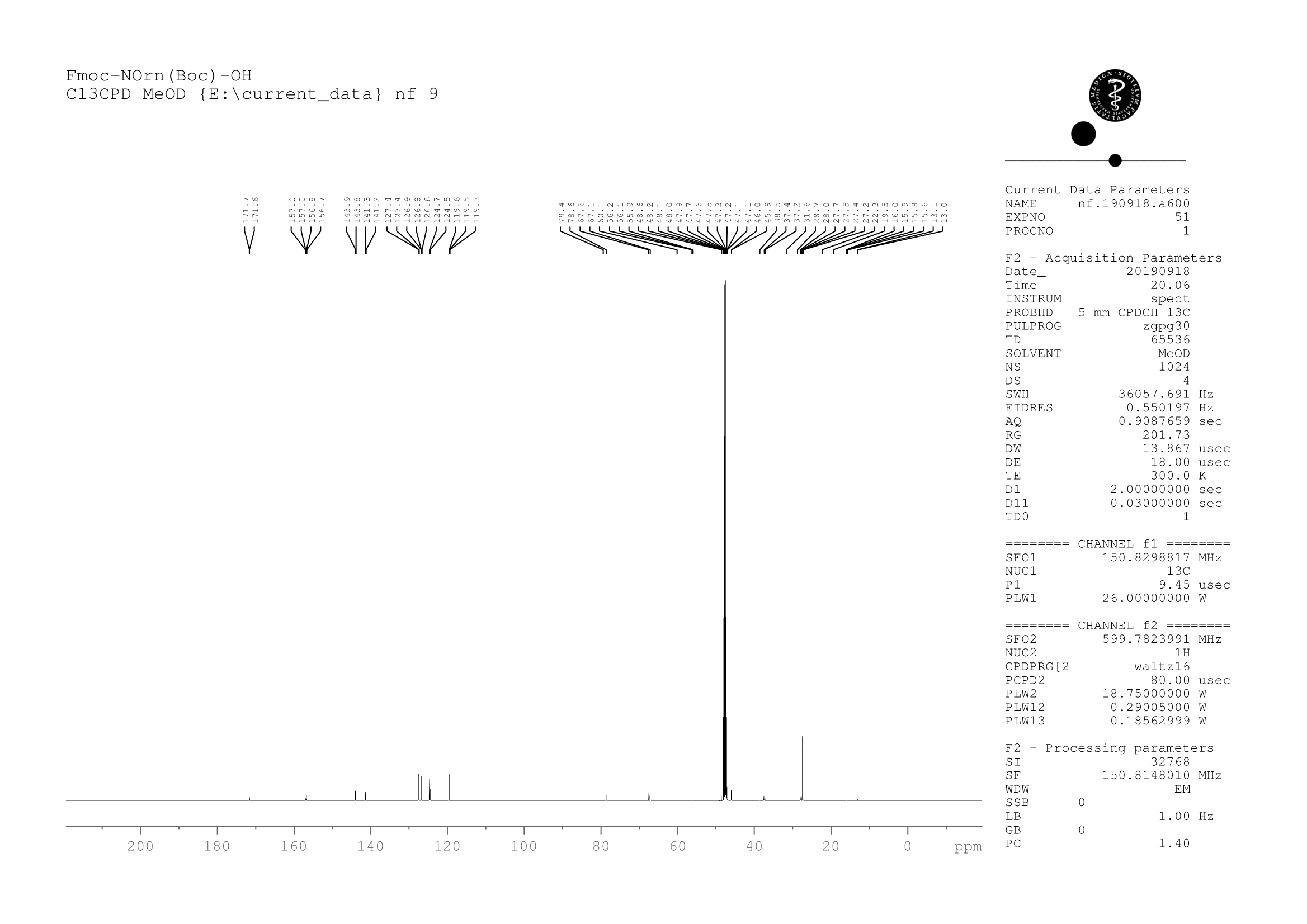


**Peptoid building block 19:** Fmoc-NLys(Boc)-OH.

**HRMS:** calculated for [M+1H]^1+^ 491.21526, found 491.21664; ∆M = 2.8 ppm.


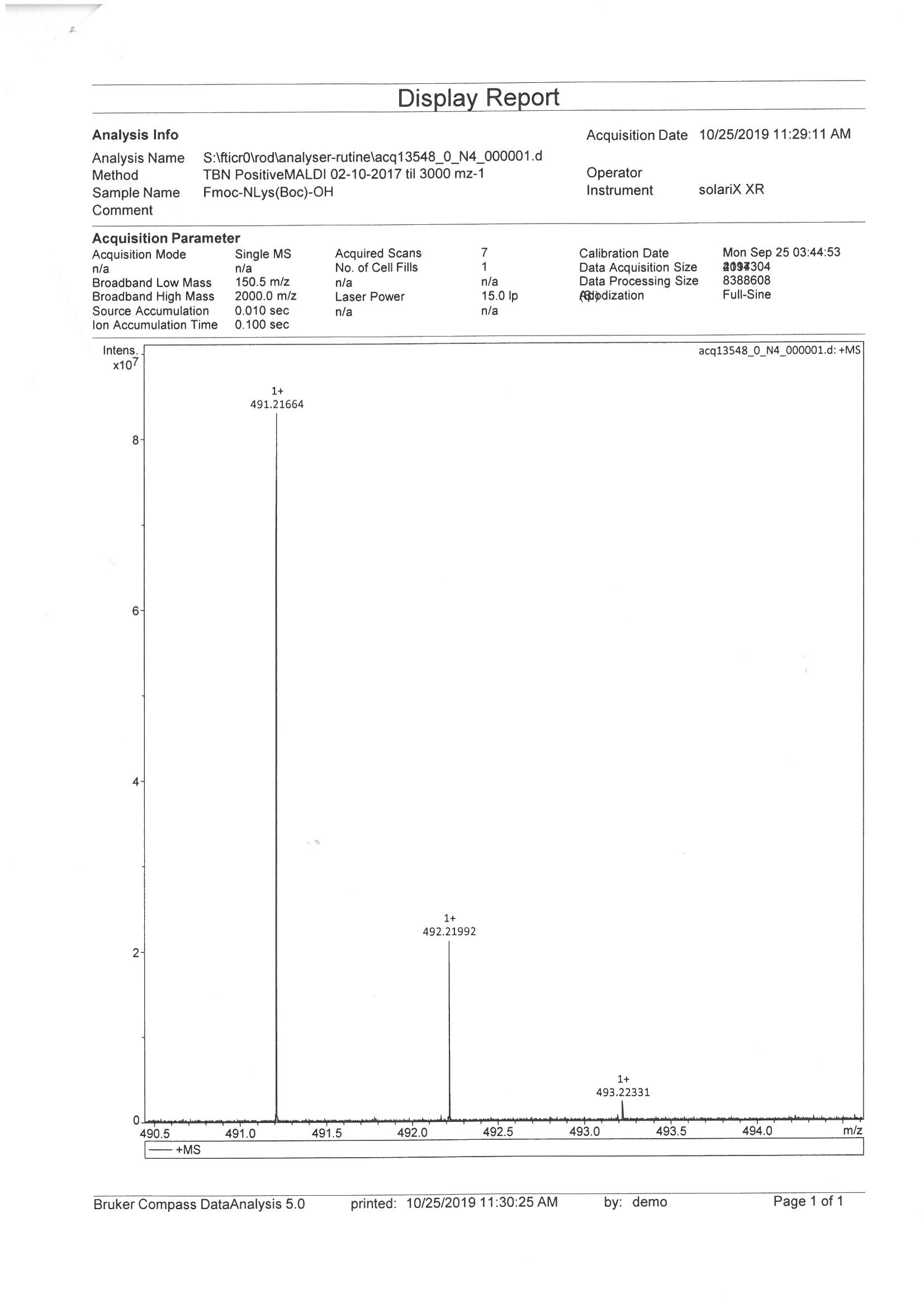


**HPLC:** t_R_ = 6.94 min, purity 100.00%. Gradient: 30-100% B during 10 min. B = 95% MeCN + 0.1% TFA.
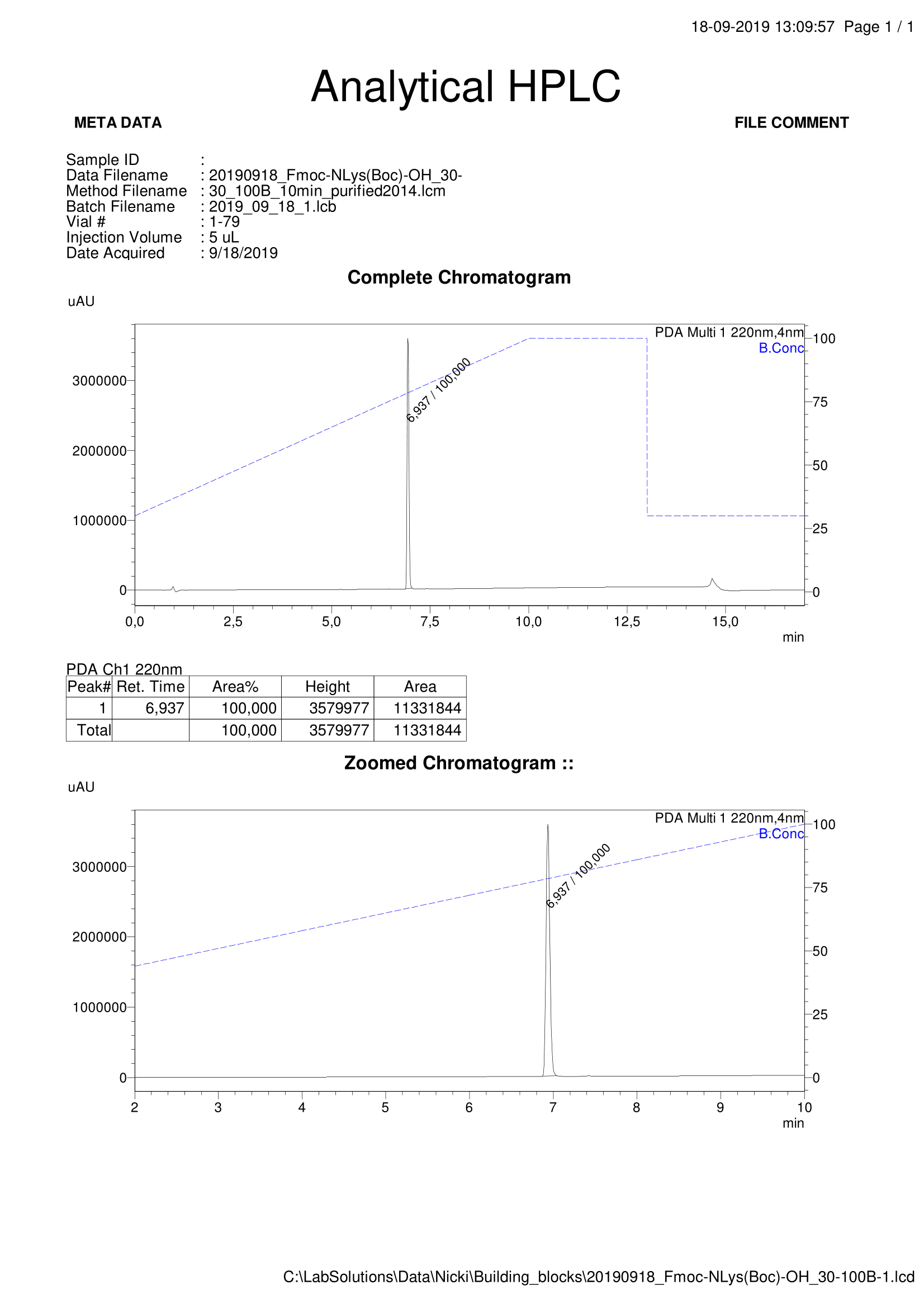


**^13^C-NMR**


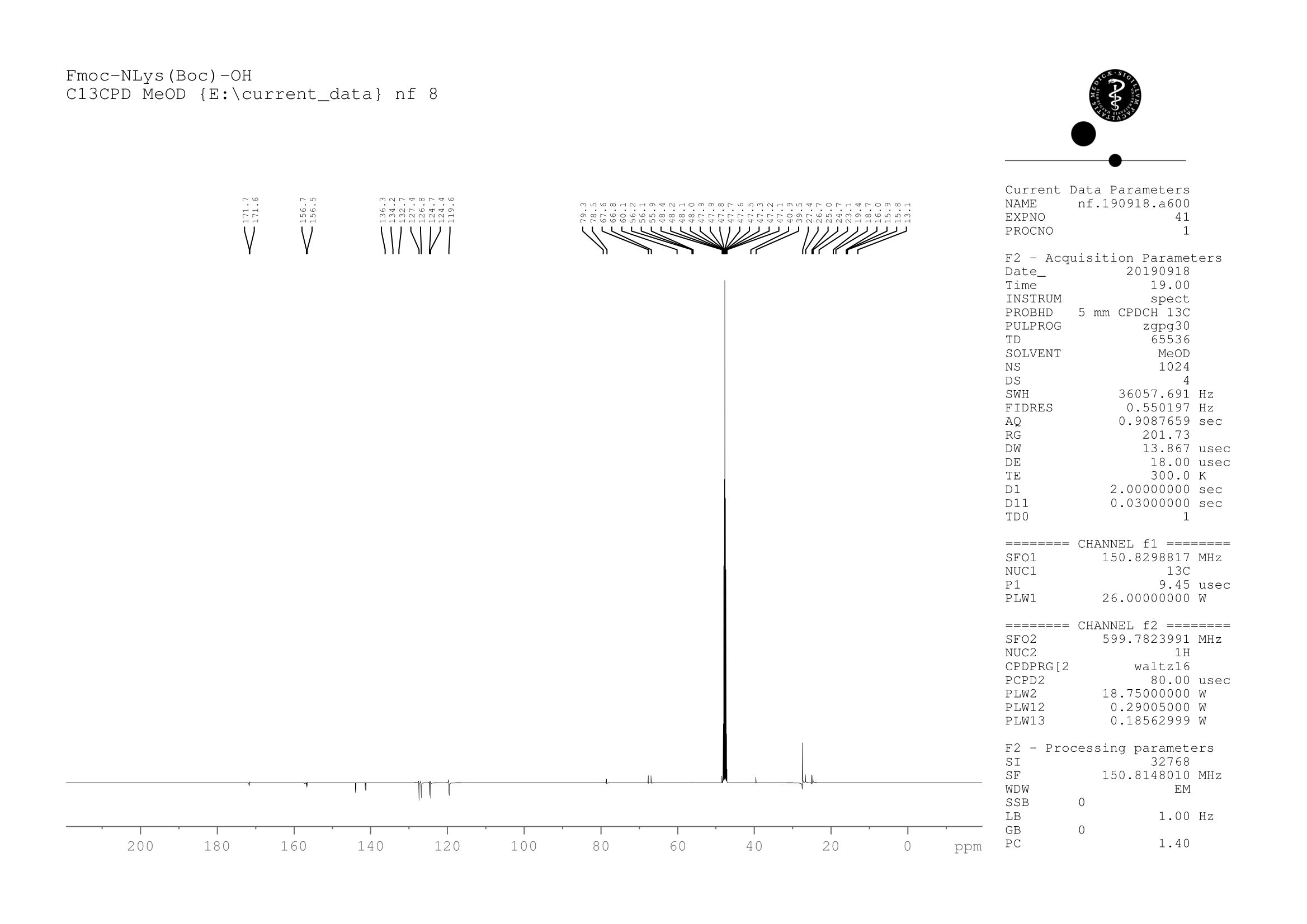


**Peptoid building block 20:** Fmoc-NhLys(Boc)-OH

**HRMS:** calculated for [M+1H]^1+^ 505.23091, found 505.2322; ∆M = 2.6 ppm.


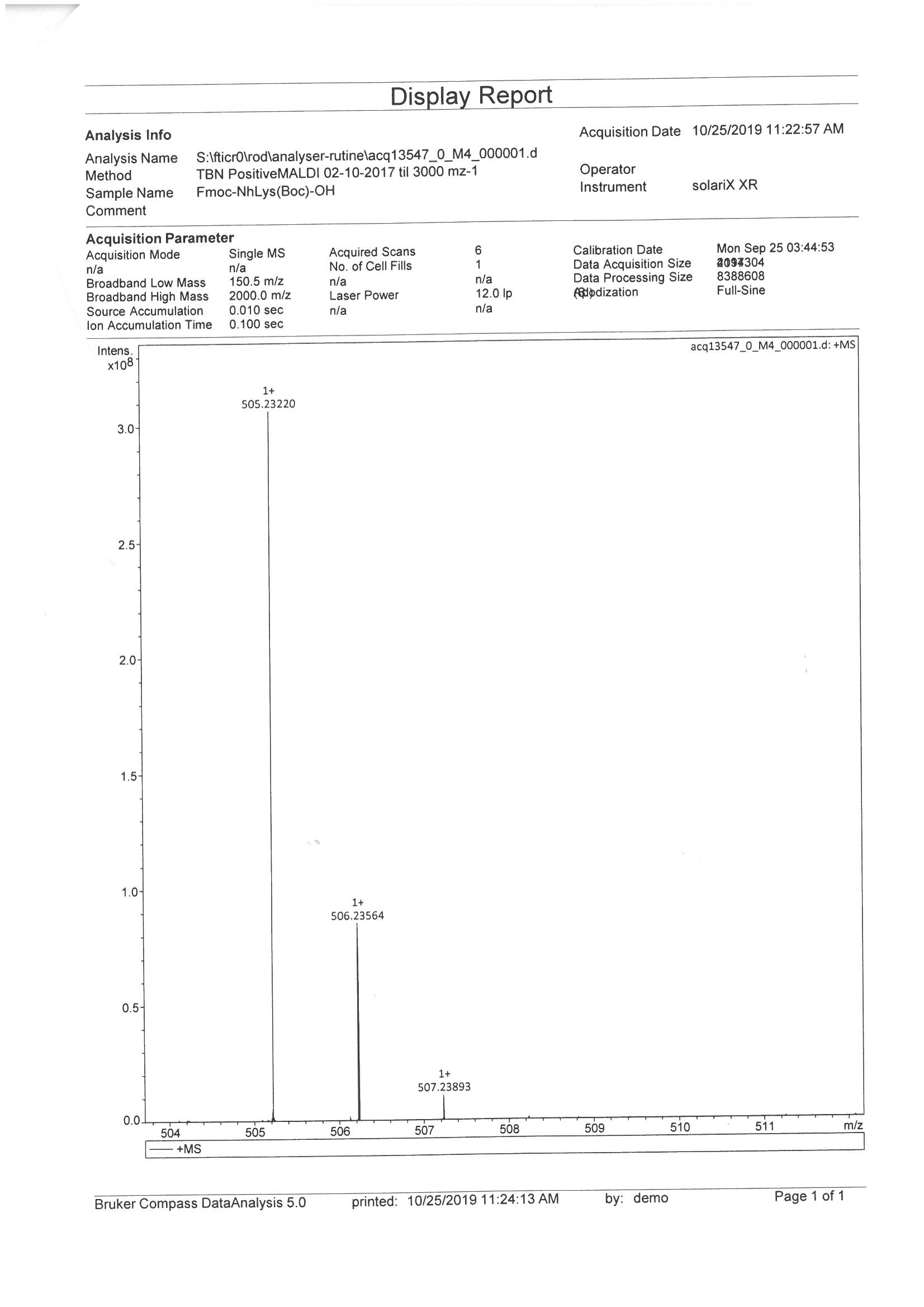


**HPLC:** t_R_ = 7.27 min, purity 100.00%. Gradient: 30-100% B during 10 min. B = 95% MeCN + 0.1% TFA.
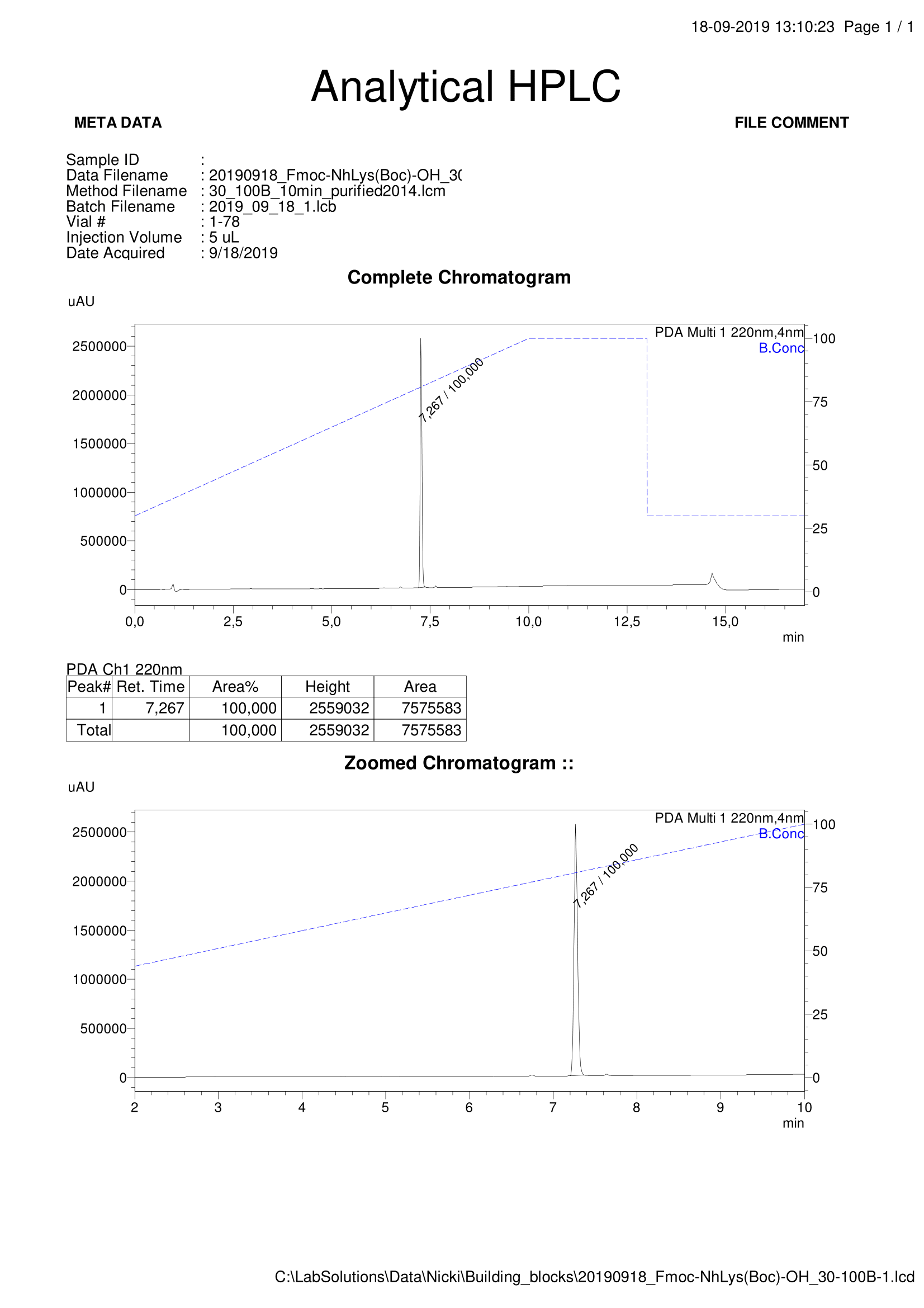


**^13^C-NMR**


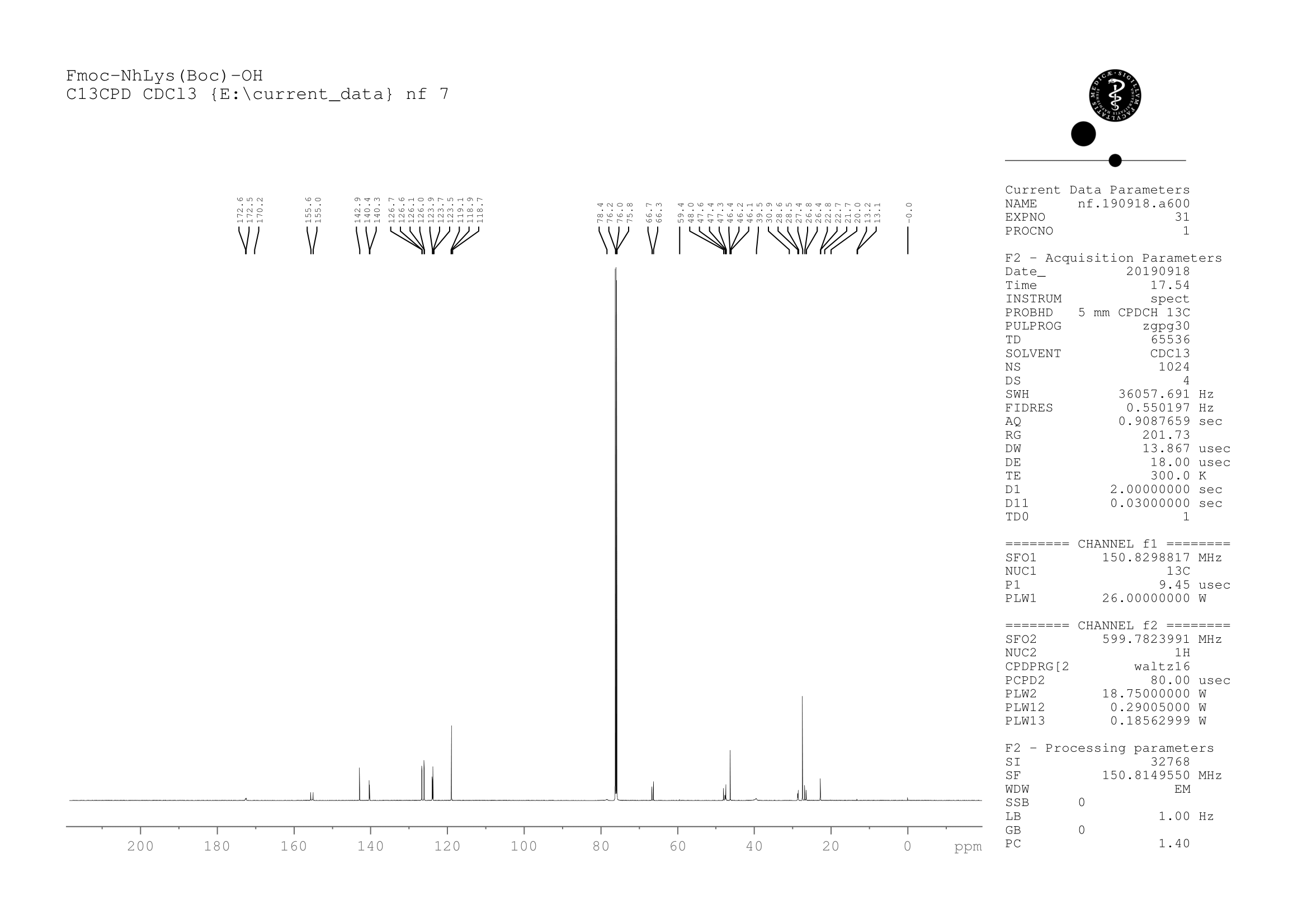


**Peptoid building block 21:** Fmoc-NPhe-OH

**HRMS:** calculated for [M+1H]^1+^ 410.13628, found 410.13852; ∆M = 5.5 ppm.


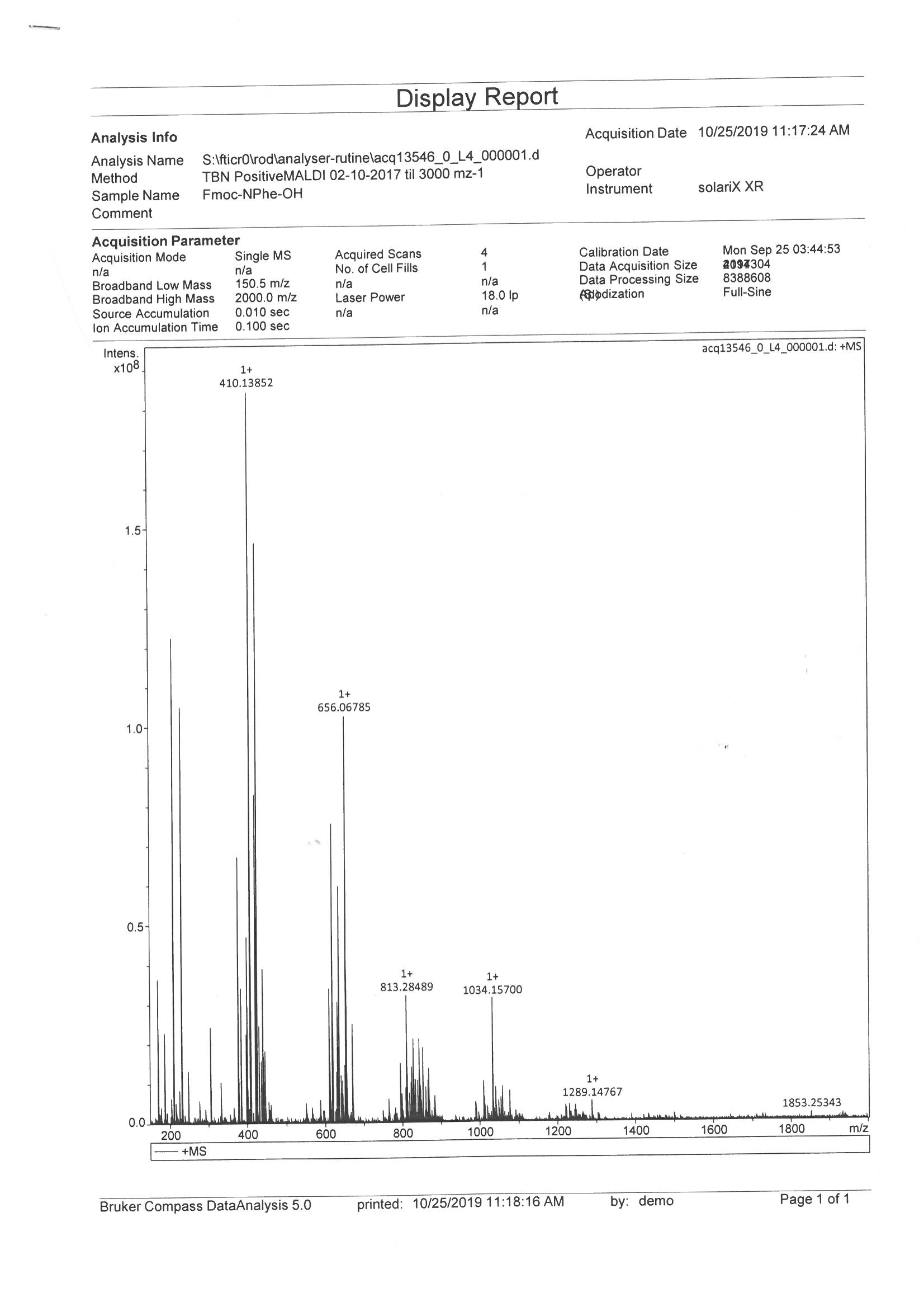


**HPLC:** t_R_ = 7.18 min, purity 100.00%. Gradient: 30-100% B during 10 min. B = 95% MeCN + 0.1% TFA.


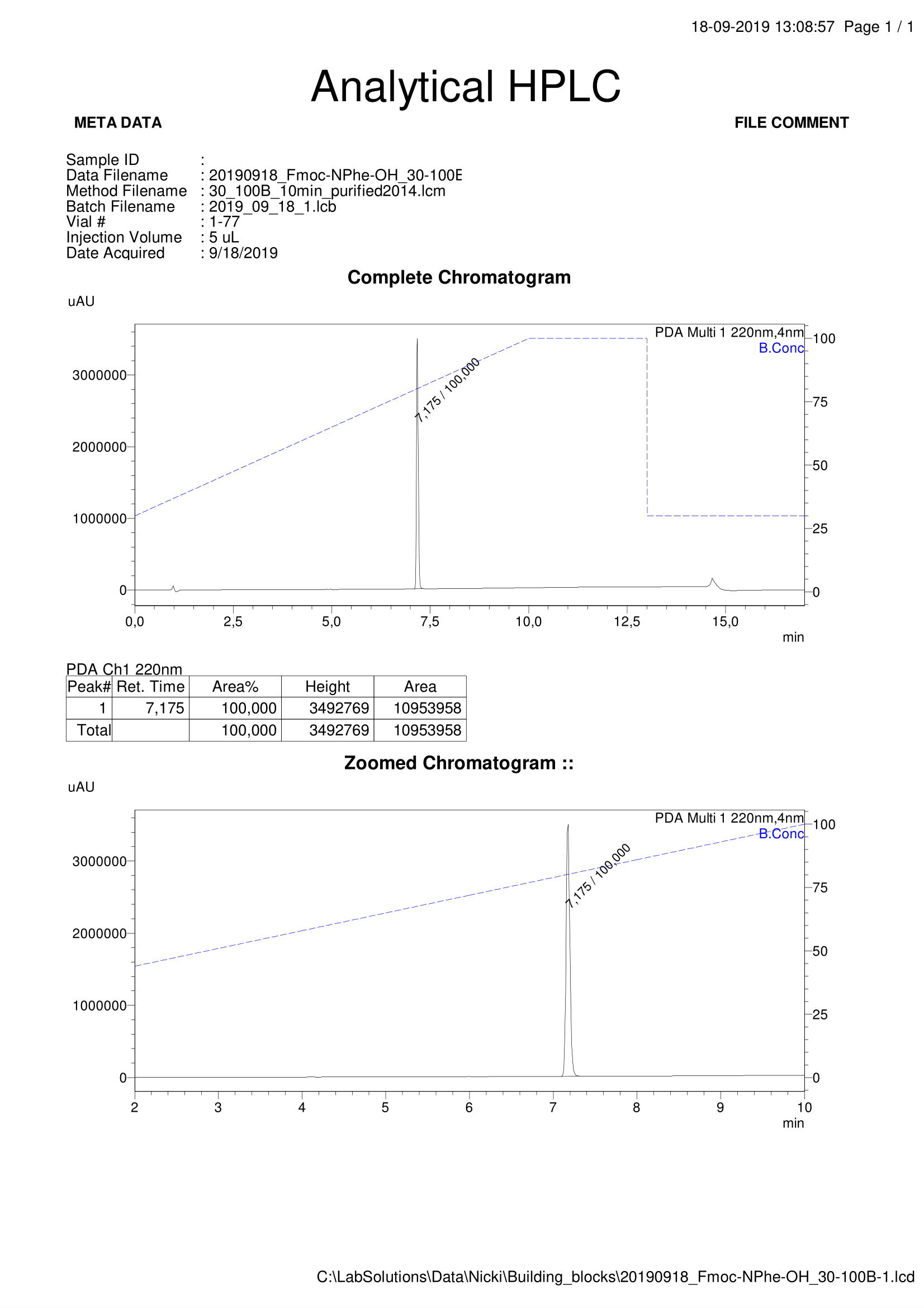


**^13^C-NMR**


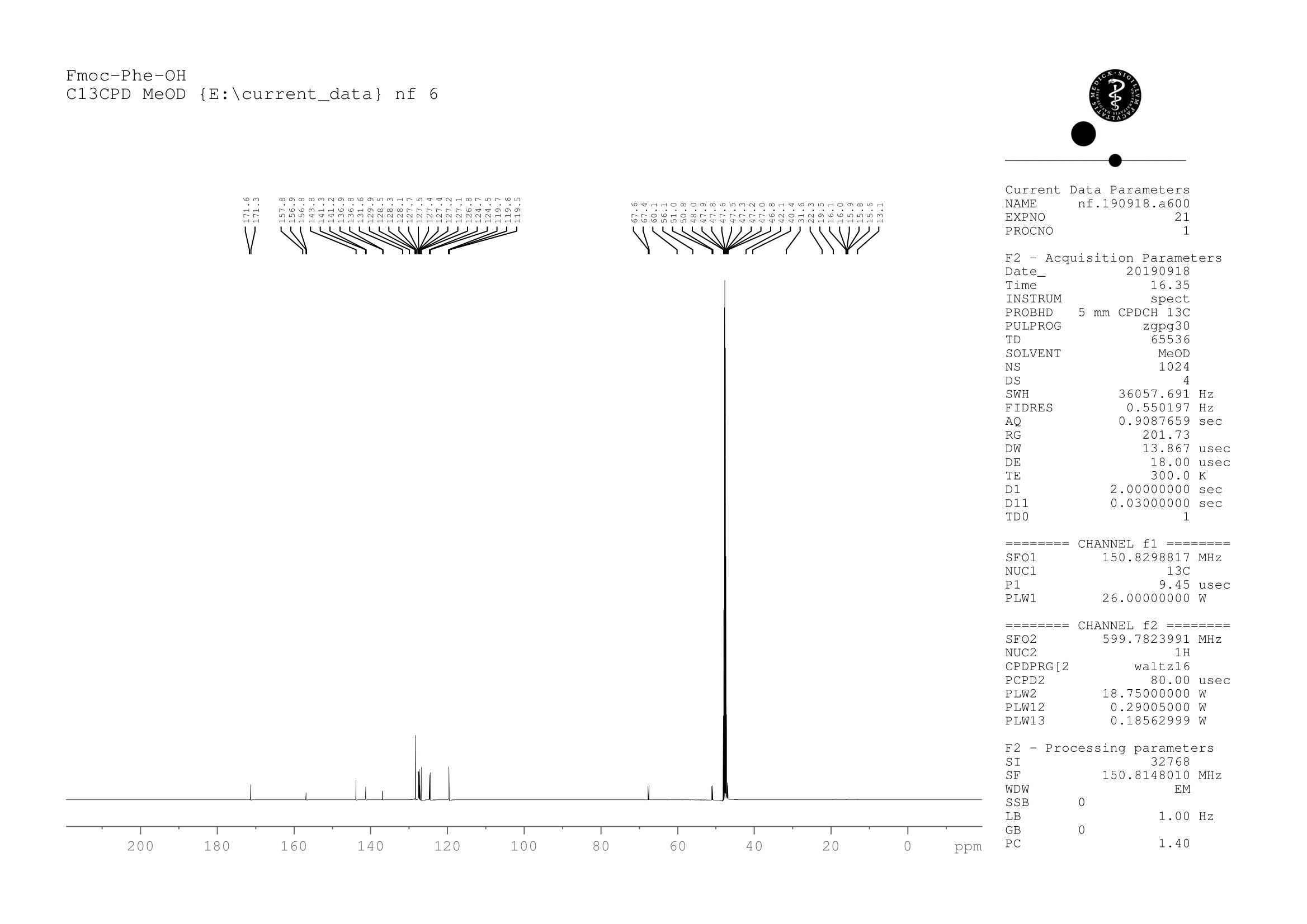


**Peptoid building block 22:** Fmoc-NhPhe-OH

**HRMS**: calculated for [M+1H]^1+^ 424.15193, found 424.15548; ∆M = 8.4 ppm.


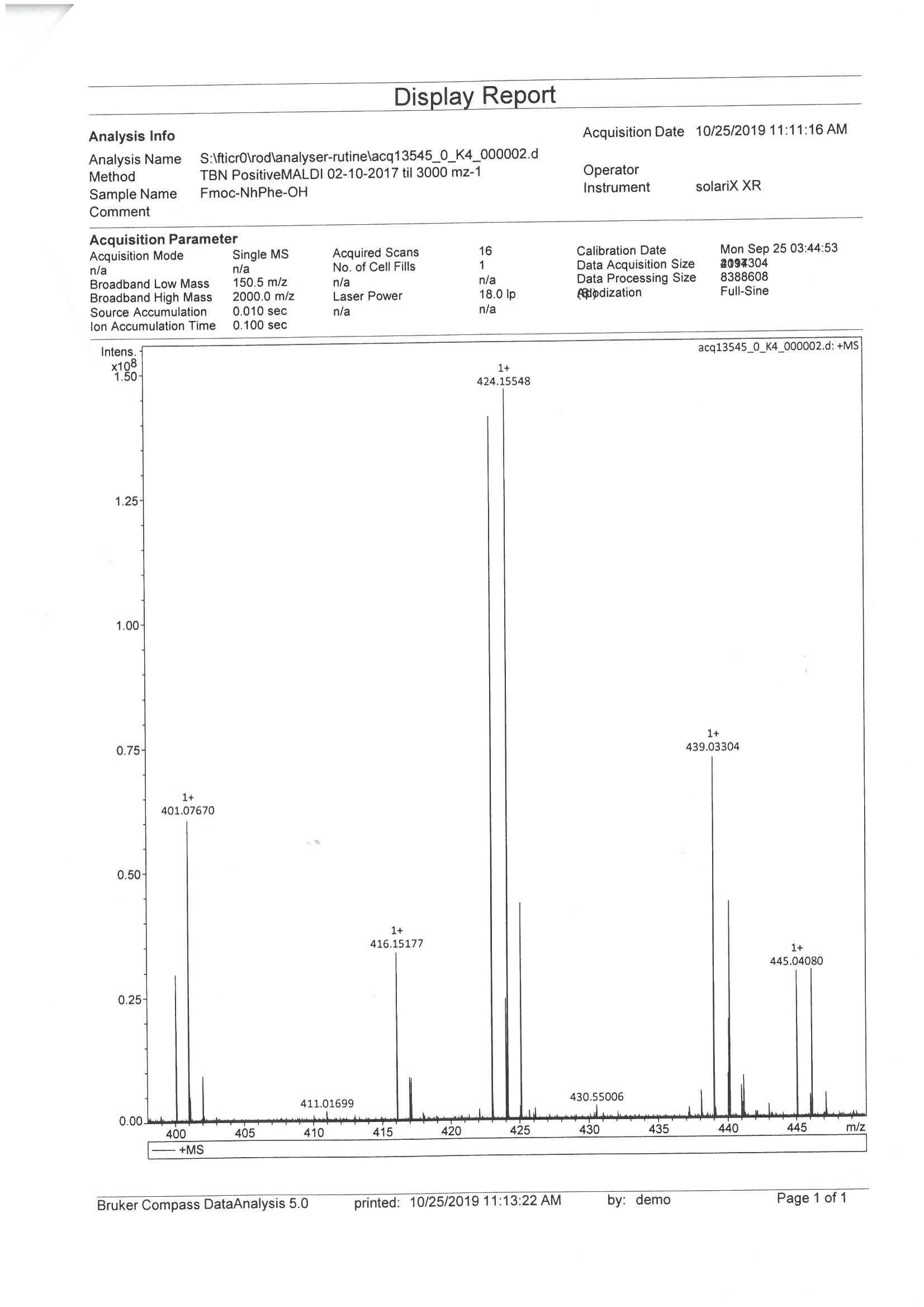


**HPLC:** t_R_ = 7.50 min, purity 100.00%. Gradient: 30-100% B during 10 min. B = 95% MeCN + 0.1% TFA.


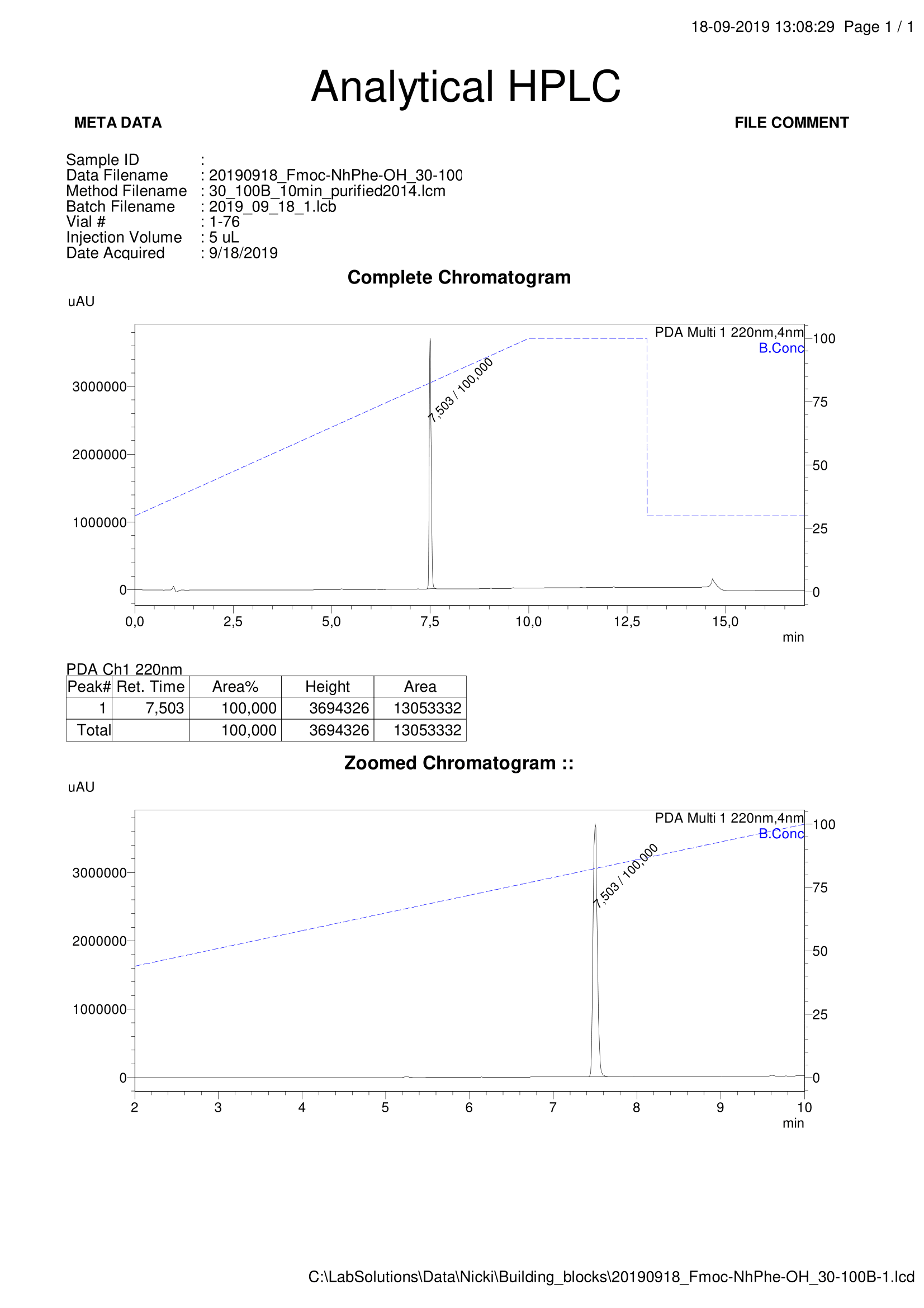


**^13^C-NMR**


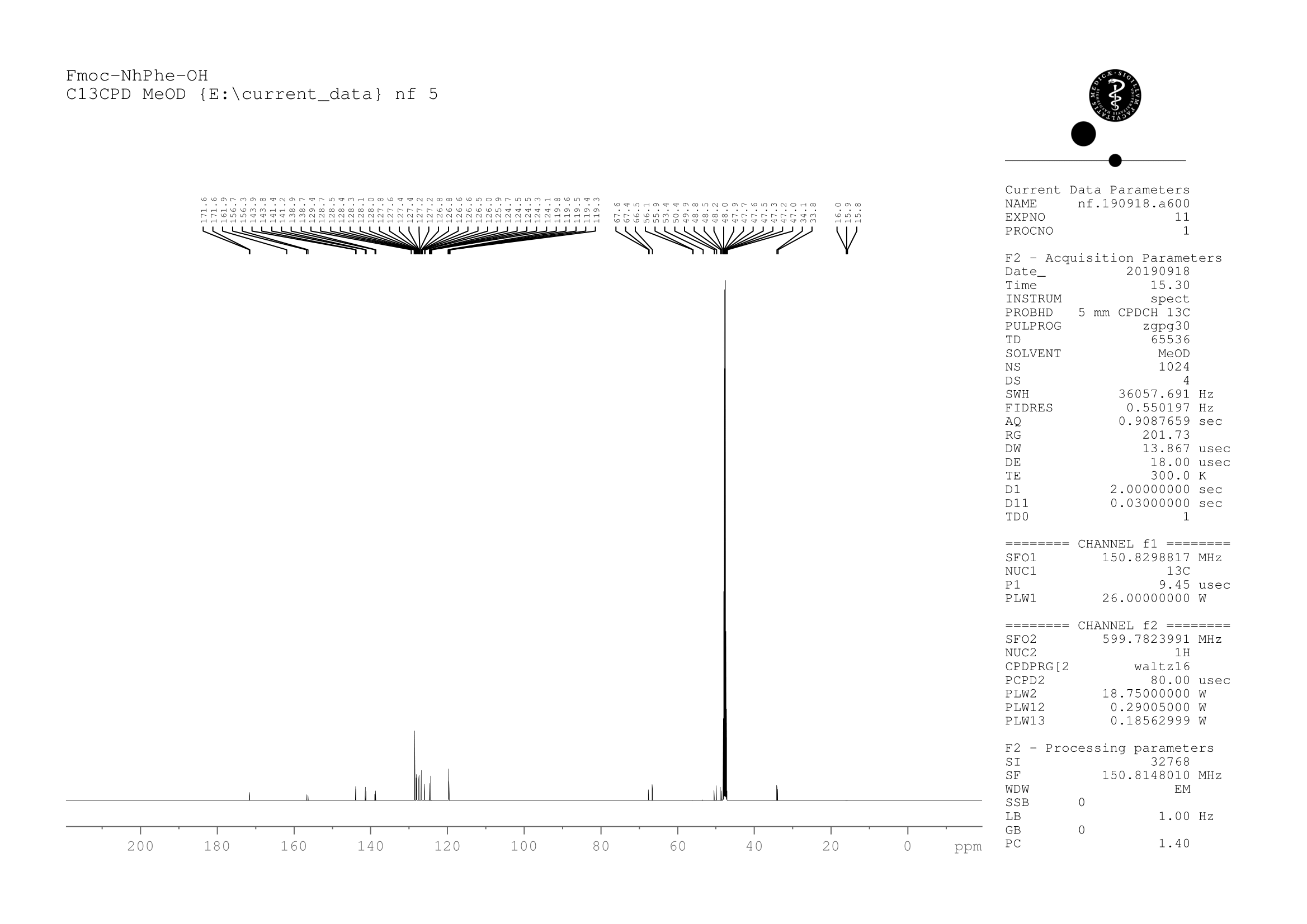

Supplement: Supplementary file 1 [file molecules-24-04429-s001.zip › Molecules_NF_HF_Suppl_Mat_20_11_2019.docx]
